# Analysis of Network Models with Neuron-Astrocyte Interactions

**DOI:** 10.1007/s12021-023-09622-w

**Published:** 2023-03-23

**Authors:** Tiina Manninen, Jugoslava Aćimović, Marja-Leena Linne

**Affiliations:** Faculty of Medicine and Health Technology, Tampere University, Korkeakoulunkatu 3, FI-33720 Tampere, Finland

**Keywords:** astrocyte, computational model, intracellular calcium, neuron-astrocyte network, simulation, synapse

## Abstract

Neural networks, composed of many neurons and governed by complex interactions between them, are a widely accepted formalism for modeling and exploring global dynamics and emergent properties in brain systems. In the past decades, experimental evidence of computationally relevant neuron-astrocyte interactions, as well as the astrocytic modulation of global neural dynamics, have accumulated. These findings motivated advances in computational glioscience and inspired several models integrating mechanisms of neuron-astrocyte interactions into the standard neural network formalism. These models were developed to study, for example, synchronization, information transfer, synaptic plasticity, and hyperexcitability, as well as classification tasks and hardware implementations. We here focus on network models of at least two neurons interacting bidirectionally with at least two astrocytes that include explicitly modeled astrocytic calcium dynamics. In this study, we analyze the evolution of these models and the biophysical, biochemical, cellular, and network mechanisms used to construct them. Based on our analysis, we propose how to systematically describe and categorize interaction schemes between cells in neuron-astrocyte networks. We additionally study the models in view of the existing experimental data and present future perspectives. Our analysis is an important first step towards understanding astrocytic contribution to brain functions. However, more advances are needed to collect comprehensive data about astrocyte morphology and physiology in vivo and to better integrate them in data-driven computational models. Broadening the discussion about theoretical approaches and expanding the computational tools is necessary to better understand astrocytes’ roles in brain functions.

**Supplementary Information:**

The online version contains supplementary material available at 10.1007/s12021-023-09622-w.

## Introduction

Modeling astrocytic functions, often together with neuronal or vascular functions, has been the trend in recent years, and, consequently, hundreds of computational models have been developed. Different aspects of these models have been reviewed before (see, e.g., Jolivet et al., 2010; Mangia et al., 2011; De Pittà et al., 2012, 2016; Fellin et al., 2012; Min et al., 2012; Volman et al., 2012; Wade et al., 2013; Linne & Jalonen, 2014; Tewari & Parpura, 2014; Manninen et al., 2018b, 2019; Denizot et al., 2020; González et al., 2020; Covelo et al., 2022; Linne et al., 2022). However, none of the previous surveys categorized and analyzed in detail all aspects of neuron-astrocyte network-level models. These aspects are: **(1)** bioelectricity in neurons, models for excitable neuronal membranes, **(2)** calcium (Ca^2+^) and other cell biological mechanisms in astrocytes, **(3)** spatial organizations of cells, **(4)** structures of functional local interaction schemes between neurons and proximal astrocytes, **(5)** structures of global interaction schemes between each pair of modeled cell types (between neurons of different types, between neurons and astrocytes), **(6)** directions of information flow, **(7)** inputs and outputs of the models (if any) including the stimulus protocols and the recorded variables, **(8)** origins and evolutions of the utilized models, **(9)** details of modeled neural systems (brain areas, developmental stages, etc.), and **(10)** availability of the model codes. To overcome this shortcoming, we decompose network models to their building blocks, systematically analyze and compare these blocks, and categorize the interactions between them. We also discuss what is missing in these computational models to explain different brain phenomena.

The computational astrocyte and neuron-astrocyte interaction models can be divided into four categories: **(1)** models describing one or several properties of a single astrocyte (shortly single astrocyte models), **(2)** models connecting at least two astrocytes together (shortly astrocyte network models), **(3)** models describing neuron-astrocyte interactions in synapses with one to several neurons and only one astrocyte (shortly neuron-astrocyte synapse models), and **(4)** models describing neuron-astrocyte interactions in regulating neuronal networks with at least two neurons and two astrocytes (shortly neuron-astrocyte network models) (see also, Manninen et al., 2018b). In our earlier studies (Manninen et al., 2017, 2018a, b, 2019; Linne et al., 2022), we have summarized and discussed all four types of models. Here, we focus in detail on the last listed category, the neuron-astrocyte network models. Models of this category are required to have at least two astrocytes and two neurons, and bidirectional interactions between the astrocytes and neurons. However, models with bidirectional neuron-astrocyte interactions that have only one modeled astrocyte are here considered to be neuron-astrocyte synapse models, although these models might have the single astrocyte connected to multiple synapses between the neuronal population (Manninen et al., 2018b). Thus, the models, where an astrocyte receives inputs from neurons but does not exert a feedback modulation on neuronal activity, could be listed into category 1 or 2, but not into 3 or 4.

In the present analysis, we are interested in neuron-astrocyte network models that include explicitly modeled astrocytic Ca^2+^ dynamics, because it is widely accepted that Ca^2+^ elevations are one of the key signaling mechanisms contributing to neuron-astrocyte interactions and linking bioelectrical phenomena with intracellular phenomena (see, e.g., Nimmerjahn, 2009; Volterra et al., 2014; Bazargani & Attwell, 2016). Modeling astrocytic Ca^2+^ dynamics is supported by accumulating electrophysiological (see, e.g., Schramm et al., 2014) and Ca^2+^ imaging (see, e.g., Poskanzer & Yuste, 2016; Agarwal et al., 2017; Arizono et al., 2020; King et al., 2020) data. Abnormalities in astrocytic Ca^2+^ signaling have been shown in neurodevelopmental disorders and neurodegenerative diseases (Allen, 2013; Finsterwald et al., 2015). Some astrocyte models that are more abstract or that consider ions other than Ca^2+^ as the putative signal-carrying ion also exist. These models are, however, excluded from our analysis because of our focus on astrocytic intracellular Ca^2+^ signaling and the large number of these Ca^2+^-oriented models. Examples of synapse- and network-level studies modeling potassium (K^+^) and sodium (Na^+^) ions include the models by Conte et al. (2018), Cui et al. (2018), Du et al. (2018), and Sætra et al. (2021).

The astrocyte and neuron-astrocyte interaction models use either biophysical or phenomenological modeling or combining both. Biophysically detailed modeling can consider several mechanisms, for example Ca^2+^ dynamics, detailed neuron-astrocyte interactions, vascular events, and K^+^ buffering, while phenomenological modeling uses simple mathematical equations describing the dynamics with fewer mechanisms, for example, to model the interactions between the neurons and astrocytes. However, multicompartmental modeling of astrocytic functions where one models the astrocytic soma, the different main-stem branches, and the extensive arborizations similarly to neuronal compartmental modeling is still in its infancy. The reason for this is that not all the morphological, biophysical, and biochemical details of astrocytes are known and that we also need standardized computational tools.

Previously, we have categorized and analyzed in detail the modeled mechanisms of astrocytes (Manninen et al., 2018b) and addressed briefly the modeled mechanisms of neurons and astrocytes (Manninen et al., 2019) of the published single astrocyte, astrocyte network, neuron-astrocyte synapse, and neuron-astrocyte network models that describe astrocytic Ca^2+^ signaling according to the criteria set by us. Here we analyze and categorize in detail the modeled mechanisms of both neurons and astrocytes and the mechanisms representing the interactions between these cells as well as the structure of the models, including the details of the spatial organization of the cells and the interaction schemes. We also analyze the evolution of all the cell models and their interactions. Our analysis presents the state of the art in modeling neuron-astrocyte networks. We emphasize, especially, the integration of experimental data about astrocyte morphology and physiology as well as the network structure when building neuron-astrocyte models, the need for standardized simulation, data-analysis, and sensitivity-analysis tools specialized in neuron-astrocyte network models, and the need for making the model implementations available in online repositories so that the modeling results are reproducible.

## Methods and Results

**Fig. 1:**
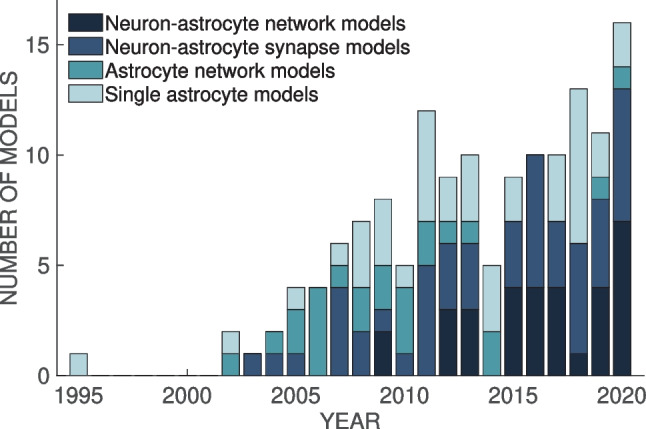
Number of astrocyte models published per year with explicitly modeled astrocytic Ca^2+^ dynamics. Numbers given for single astrocyte models, astrocyte network models, neuron-astrocyte synapse models, and neuron-astrocyte network models

Early models that include astrocytic Ca^2+^ dynamics belong to either single astrocyte, astrocyte network, or neuron-astrocyte synapse category. The first single astrocyte model was published in 1995 (Roth et al., 1995), while the first astrocyte network and neuron-astrocyte synapse models appeared in 2002 (Höfer et al., 2002) and 2003 (Nadkarni & Jung, 2003), respectively (Fig. 1). It is not surprising that neuron-astrocyte network models integrating astrocytic and neuronal dynamics with mechanisms of neuron-astrocyte and often also astrocyte-astrocyte exchange started appearing later. The first neuron-astrocyte network models were published in 2009 (Allegrini et al., 2009; Postnov et al., 2009) and became more frequent in recent years – almost half of the models published in 2020 belonged to this category. Overall, the interest in computational models that incorporate astrocytic mechanisms is steadily increasing, as can be seen in Fig. 1. During the last years, the number of published models increased for each category except for the astrocyte network models.

In the following sections, we explain the criteria of choosing the models into our study and the characteristics of these models, we summarize the neuronal and astrocytic mechanisms and models used in the studies, we describe the mechanisms and models used to implement the functional interactions between cells, and we analyze and systematize the presentation for the structure of the models, including the spatial organization of the modeled cells and the interaction scheme between modeled cells. Lastly, we explain which neural functions were studied with the models.

### Selection of Models

As in our previous studies (Manninen et al., 2018b, 2019), we here limited our evaluation to models which had at least one astrocytic intracellular Ca^2+^ mechanisms modeled and the astrocytic intracellular Ca^2+^ signaling was described by a differential equation that was a function of time, Ca^2+^ itself, and at least one of the other astrocytic variables which could be, for example, inositol trisphosphate (IP_3_). In addition, astrocytic Ca^2+^ had to have an impact on some signaling variable or other intracellular signal in the astrocytes. Neuron models had to include at least one differential equation for some variable, for example for membrane potential. Furthermore, the models had to include at least two neurons and two astrocytes as well as bidirectional neuron-astrocyte interactions to form neuron-astrocyte network models. Based on these criteria, we ended up having 32 neuron-astrocyte network models published by the end of 2020.

### Characteristics of Models

We categorized and characterized the differences of these 32 neuron-astrocyte network models in all aspects, including neuronal and astrocytic cellular mechanisms and models, all types of functional interactions between modeled neurons and astrocytes in synaptic and non-synaptic communications, and details of structural organization of interactions between all modeled cell types. See the full biological description of astrocytes and neuron-astrocyte interactions, for example, in the reviews by Kettenmann and Ransom (2013), Volterra et al. (2014), Bazargani and Attwell (2016), and Verkhratsky and Nedergaard (2018).

Table 1 lists several characteristics of the neuron-astrocyte network models. For each model, we indicated whether the model was built for certain brain area in mind, whether experimental data was used to validate simulation results, how many neurons of each type were modeled (excitatory neuron (E), inhibitory neuron (I), interneuron (IN), pyramidal neuron (PY), thalamocortical neuron (TC), and reticular thalamic neuron (RE)), how many astrocytes were modeled, and which experimentally shown neural function was studied with the model, for example Ca^2+^ dynamics (Ca^2+^), excitatory-inhibitory balance (E-I balance), synchronization (Sync.), signal or information transfer (Sgn./Inf.), synaptic plasticity (Plast.), or hyperexcitability (Hyper.), or was the model built to solve a classification task (Classif.) or to support hardware implementation (HW). We also listed which programming language or simulation tool was used to implement the models and whether the model codes were available in open-access online repositories. Only details that were clearly given in the model publications are presented in Table 1. Out of the 32 models, seven named the simulation tool or programming language used and only for two of the models the model codes were available online (Aleksin et al., 2017; Stimberg et al., 2019). Aleksin et al. (2017) developed and used Arachne (C++, MATLAB^®^) to implement their model and made their model code available both as a supplementary to their article and in GitHub (https://github.com/LeonidSavtchenko/Arachne/tree/master/ExamplePLOS). Stimberg et al. (2019) implemented their model with Brian 2 (Goodman & Brette, 2008) and made their model code available in GitHub (https://github.com/mdepitta/comp-glia-book/tree/master/Ch18.Stimberg). Nine models were specialized to cerebral cortex, eight to hippocampus, one to spinal cord, and two to thalamocortical networks, while 12 models were generic models not developed for any specific brain area. Only two of the studies compared the simulation results to experimental data either qualitatively or quantitatively (Amiri et al., 2013a; Chan et al., 2017). Amiri et al. (2013a) compared their model to local field potential (LFP) recordings from rat hippocampal cornu ammonis 1 (CA1) brain slices in vitro. Chan et al. (2017) compared their model to multi-electrode array (MEA) recordings from dissociated cortical cultures of Wistar rat embryos at day 18. In addition, bifurcation analysis was done with a couple of models (see, e.g., Amiri et al., 2012b; Hayati et al., 2016; Li et al., 2016; Tang et al., 2017; Makovkin et al., 2020) which, in general, helps in understanding the dynamical behavior of the models.

### Cell Models

The choice of modeled cells depended on the study and might include, for example, the whole neurons, the whole astrocytes, neuronal or astrocytic compartments, and pre- and postsynaptic compartments. Most of the analyzed studies relied on the single-compartmental models for both neurons and astrocytes, however, some studies also explored multicompartmental models for astrocytes with either simple or more detailed morphologies (see, e.g., Postnov et al., 2009; Liu & Li, 2013a, b; Mesiti et al., 2015; Gordleeva et al., 2019). The number of neuronal cell types in the analyzed models ranged between 1–4 (most often two types), while astrocytes always belonged to a single type. Within the same cell type, the number of cells was from one or a few (see, e.g., Liu & Li, 2013b; Makovkin et al., 2020) up to several thousand (see, e.g., Chan et al., 2017; Nazari et al., 2020). The numbers and types of cells are summarized in Table 1.

#### Modeled Neuronal Mechanisms

**Fig. 2:**
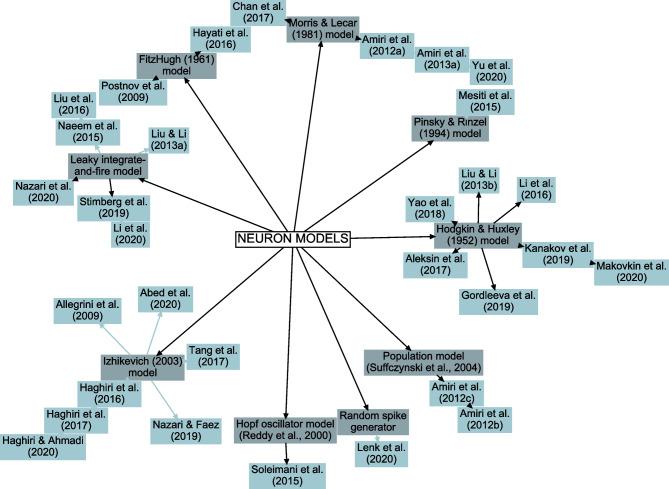
Evolution of neuron models in the neuron-astrocyte network models. The dark (gray) colored models represent a set of models that were utilized when building the neuron models of the light (blue) colored neuron-astrocyte network models. The light (blue) colored arrows mean that the two neuron models have the same general structure. The black arrows mean that the two models are partly the same. However, note that we did not classify the similarity between different dark (gray) colored models, but only for the rest of the connections. The complete picture of the models used to construct the neuron models is given in Supplementary Table S1. In addition, there was one model that did not explain the details of the model and is excluded from this evolutionary presentation

Various modeling strategies were used to represent the neurons (Fig. 2 and Supplementary Table S1). Seven studies utilized Hodgkin-Huxley models (Hodgkin & Huxley, 1952) and one utilized Pinsky-Rinzel model (Pinsky & Rinzel, 1994) derived from the model by Traub et al. (1991). Four studies used Morris-Lecar model (Morris & Lecar, 1981), two studies used FitzHugh-Nagumo model (FitzHugh, 1961), six studies used leaky integrate-and-fire (LIF) models, and seven studies used Izhikevich model (Izhikevich, 2003). Less common choices were the use of Hopf oscillator by Reddy et al. (2000) in one model, random spike generator in one model, and the population model by Suffczynski et al. (2004), which is an extension of LIF neurons, in two models.

Supplementary Table S2 lists for each model the inputs used for the modeled neurons, such as applied, synaptic, and astrocytic currents, neuronal variables and other variables representing, for example, molecules released from neurons described by differential equations, as well as neuronal ionic currents, and the outputs of the neurons. Almost half of the studies explicitly modeled neuronal ion channel kinetics using Hodgkin-Huxley formalism or simplifications of it. Among the modeled channels were T-type low-threshold Ca^2+^ (Ca_T_) channels, transient K^+^ (K_A_) channels, Ca^2+^-activated K^+^ (K_Ca_) channels, delayed rectifier K^+^ (K_DR_) channels, afterhyperpolarization (AHP) channels, persistent Na^+^ (Na_P_) channels, and fast transient Na^+^ (Na_T_) channels. Two models also included differential equations to explicitly represent Ca^2+^ concentration (Mesiti et al., 2015) or K^+^ and Na^+^ concentrations (Yao et al., 2018). Two models included endocannabinoid 2-arachidonoylglycerol (2-AG) in their postsynaptic neurons (Naeem et al., 2015; Liu et al., 2016). The outputs of neuron models were mostly synaptic currents and neurotransmitters. Neurotransmitters were listed as neuronal variables if modeled with differential equations, and in many models, neurotransmitters were used as inputs to the other cells. For example, glutamate in the synaptic cleft ([Glu]_syn_) or neurotransmitter (NT) was used as the input to activate the astrocytes.

#### Modeled Astrocytic Mechanisms

Astrocytes express all major types of ion channels, such as K^+^, Na^+^, and Ca^2+^ channels, as well as various types of anion and chloride (Cl^−^) channels, aquaporins, transient receptor potential channels, and non-selective channels. In addition to different channels, astrocytes also express adenosine and adenosine triphosphate (ATP)-dependent transporters on the plasma membrane, such as the Na^+^/K^+^-ATPase and plasma membrane Ca^2+^-ATPase (PMCA), and sarco/endoplasmic reticulum (ER) Ca^2+^-ATPase (SERCA) on the ER membrane which are important to the Ca^2+^ excitability of astrocytes. Other so-called secondary transporters include glutamate transporters, such as excitatory amino acid transporters, as well as, for example, gamma-aminobutyric acid (GABA) transporters, glycine transporters, Na^+^/Ca^2+^ exchangers, and Na^+^/K^+^/Cl^−^ cotransporters. Astrocytes have been shown to express various ionotropic and metabotropic receptors, such as glutamate, GABA, glycine, acetylcholine, adrenergic, serotonin, histamine, cannabinoid, and neuropeptide receptors, and purinoceptors for adenosine and ATP. Even though astrocytes are not able to fire regenerative action potentials, the activation of their membrane mechanisms results in, for example, intracellular Ca^2+^ oscillations that depend on the Ca^2+^-induced Ca^2+^ release (CICR) via IP_3_ receptors (IP_3_Rs), mechanisms related to mitochondria, and Ca^2+^ influx, for example, via voltage-gated Ca^2+^ channels (Aguado et al., 2002; Agulhon et al., 2008; Agarwal et al., 2017; Arizono et al., 2020). Actually, Ca^2+^-mediated signals are thought to be the main communication mechanisms between astrocytes and other cells (Nimmerjahn, 2009; Volterra et al., 2014; Bazargani & Attwell, 2016). However, none of the studied neuron-astrocyte models took all these biological details into account because of computational burden. The lack of experimental data and detailed data-based models also pose an obstacle to more realistic modeling of astrocytic mechanisms.

**Fig. 3:**
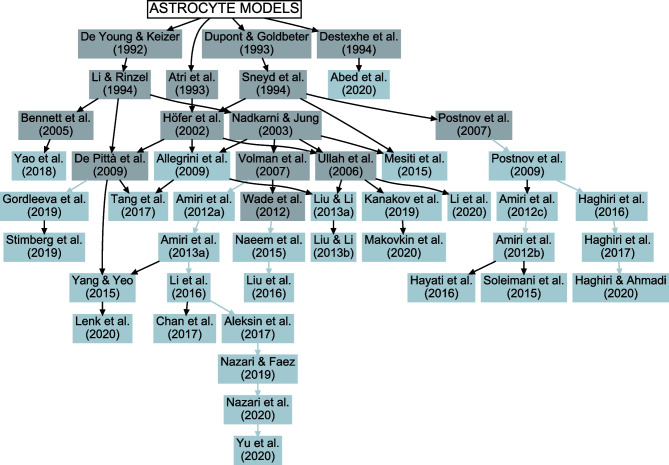
Evolution of astrocyte models in the neuron-astrocyte network models. The dark (gray) colored models represent a set of models that were utilized when building the astrocyte models of the light (blue) colored neuron-astrocyte network models. The light (blue) colored arrows mean that the astrocyte model above and the model below the arrow have the same Ca^2+^, IP_3_, and IP_3_R equations. The black arrows mean that the model above is partly or completely used by the model below, but the equations are not the same. We only considered the general structure of the used astrocyte models here, so from where the Ca^2+^, IP_3_, and IP_3_R equations were taken from. However, note that we did not classify the similarity between different dark (gray) colored models, but only for the rest of the connections. The complete picture of the models used to construct the astrocyte models is given in Supplementary Table S1. Some of the studies presented several modifications of their models, here we only present the details of one version per study

Most of the astrocyte models resemble closely the Ca^2+^ dynamics models originally developed for other cells, such as neurons, oocytes, or epithelial cells (Fig. 3 and Supplementary Table S1; De Young & Keizer, 1992; Atri et al., 1993; Dupont & Goldbeter, 1993; Destexhe et al., 1994; Li & Rinzel, 1994; Sneyd et al., 1994). In addition, the astrocyte models by Höfer et al. (2002), Nadkarni and Jung (2003), Bennett et al. (2005), Ullah et al. (2006), Postnov et al. (2007), Volman et al. (2007), De Pittà et al. (2009), and Wade et al. (2012), that were built based on the above models, were also used when building the network models. All these models originate from the CICR model by Bezprozvanny et al. (1991). Thus, in the end, almost all astrocytic Ca^2+^ dynamics models have evolved from the same mathematical equations with little or no tuning of the parameter values.

Supplementary Table S3 lists for each model the inputs used to activate the astrocytes, astrocytic variables and other variables representing, for example, molecules released from astrocytes described by differential equations, as well as astrocytic Ca^2+^ mechanisms related to cytosolic Ca^2+^, astrocytic IP_3_ mechanisms, diffusion of astrocytic variables either in the cytosol or ER, and outputs of the astrocytes. The astrocytic Ca^2+^ dynamics models mostly had the same general mathematical structure, with some models adding a few additional mechanisms on top of the commonly modeled IP_3_Rs (CICR), SERCA pumps, and the leak flux from the ER to the cytosol. Examples of the additional plasma membrane mechanisms are PMCA pumps (Yao et al., 2018), capacitive Ca^2+^ entry (CCE) (Kanakov et al., 2019; Makovkin et al., 2020), and K^+^ and Na^+^ channels (Yao et al., 2018). About half of the models had influx of Ca^2+^ from extracellular space or efflux of Ca^2+^ to extracellular space. Intracellular diffusion of Ca^2+^ and IP_3_ was included in six models (Allegrini et al., 2009; Postnov et al., 2009; Liu & Li, 2013a, b; Mesiti et al., 2015; Gordleeva et al., 2019). Gliotransmitters were itemized as astrocytic model variables if they were modeled with differential equations, and in many models they were used as inputs to activate other cells. We did not list the transport of molecules or ions through a membrane under diffusion, but we listed, for example, different Ca^2+^ fluxes over the plasma membrane and Ca^2+^ movement via gap junctions under the attribute ‘Ca^2+^ mechanisms’ in Supplementary Table S3. We also categorized gap junctions under ‘IP_3_ mechanisms’ in Supplementary Table S3 if IP_3_ was passed via gap junctions between astrocytes.

### Interactions between Cells

In the network models, neurons and astrocytes can interact through various synaptic mechanisms, including the interactions from pre- to postsynaptic neurons, uni- or bidirectional interactions between presynaptic terminals and astrocytes, interactions from astrocytes to postsynaptic terminals, as well as bidirectional gap junctions between astrocytes. These mechanisms are provided in detail in Table 2 and references in Supplementary Table S1.

#### Mechanisms of Functional Interaction between Modeled Neurons and Astrocytes

**Fig. 4:**
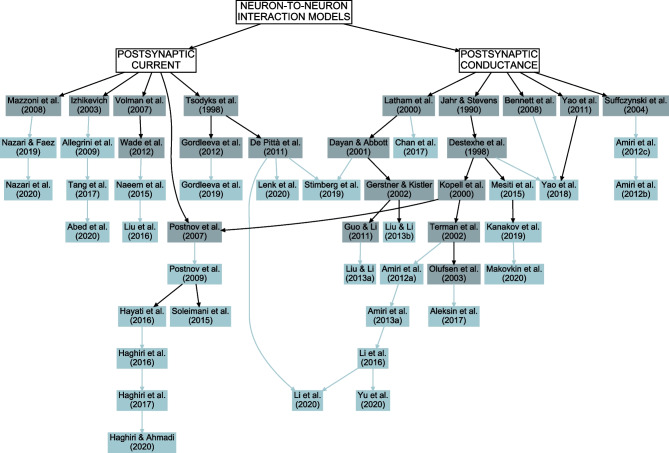
Evolution of neuron-to-neuron interactions in the neuron-astrocyte network models. The models established the interactions through change in postsynaptic current or through postsynaptic conductance. The dark (gray) colored models represent a set of models that were utilized when building the neuron-to-neuron interaction models of the light (blue) colored neuron-astrocyte network models. The light (blue) colored arrows mean that the neuron-to-neuron interaction model above and the model below the arrow have equations that are the same. The black arrows mean that the model above is partly or completely used by the model below, but the equations are not the same. We grouped the dark (gray) colored models based on the similarity. However, note that we did not classify the similarity between different dark (gray) colored models with different colored arrows, but only for the rest of the connections. The complete picture of the models used to construct the neuron-to-neuron interaction models is given in Supplementary Table S1. In addition, there was one model that did not explain the details of the model and is excluded from this evolutionary presentation

To decrease computational burden in neuron-astrocyte networks, cellular interactions were described phenomenologically, without detailed representation of involved molecular species and cellular mechanisms. Table 2 categorizes details of the mechanisms modeled between different cells, so how neurons activated other neurons and astrocytes and how astrocytes activated other astrocytes and neurons. In the case of neuron-to-neuron interactions, about half of the models established the interactions through changes in postsynaptic conductances, while the other half used postsynaptic currents (Fig. 4, Table 2, and Supplementary Tables S1 and S2). Model neurons that interacted through changes in the postsynaptic conductances, the conductance-based models, are common in the computational literature (Jahr & Stevens, 1990; Destexhe et al., 1998; Kopell et al., 2000; Latham et al., 2000; Dayan & Abbott, 2001; Gerstner & Kistler, 2002; Terman et al., 2002; Olufsen et al., 2003; Suffczynski et al., 2004; Guo & Li, 2011; Yao et al., 2011). In addition, also some earlier neuron-astrocyte synapse and network models were used to define components of the here analyzed models (see, e.g., De Pittà et al., 2011). These earlier neuron-astrocyte interaction models were not included in our study as their astrocyte models did not fulfill our criteria defined in “Selection of Models”. Models that described neuron-to-neuron interactions as postsynaptic currents were also based on neuronal network models from the literature (Tsodyks et al., 1998; Izhikevich, 2003; Mazzoni et al., 2008) and on earlier neuron-astrocyte synapse and network models that did not make into our study (Postnov et al., 2007; Volman et al., 2007; De Pittà et al., 2011; Gordleeva et al., 2012; Wade et al., 2012). As an example, some of the current-based models used the synaptic activation variables (*z*) developed by Postnov et al. (2007, 2009) based on the model by Kopell et al. (2000). Half of the studies explicitly modeled the released neurotransmitter, among them Stimberg et al. (2019), Lenk et al. (2020), and Li et al. (2020) used the well-known computational model introduced by Tsodyks et al. (1998). Aleksin et al. (2017), Gordleeva et al. (2019), and Makovkin et al. (2020) used simplified versions of the model by Tsodyks et al. (1998).

**Fig. 5:**
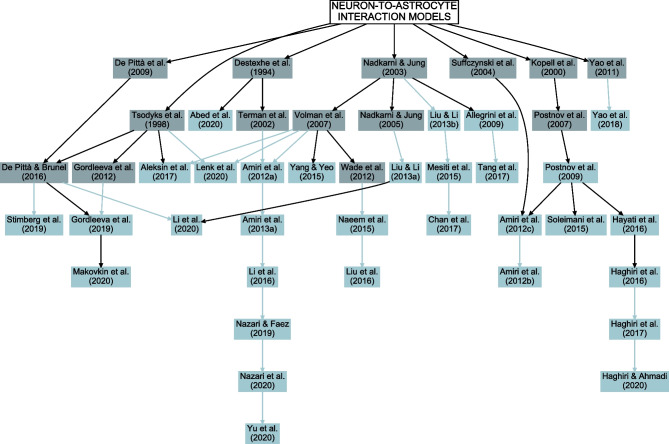
Evolution of neuron-to-astrocyte interactions in the neuron-astrocyte network models. The dark (gray) colored models represent a set of models that were utilized when building the neuron-to-astrocyte interaction models of the light (blue) colored neuron-astrocyte network models. The light (blue) colored arrows mean that the neuron-to-astrocyte interaction model above and the model below the arrow have equations that are the same. The black arrows mean that the model above is partly or completely used by the model below, but the equations are not the same. However, note that we did not classify the similarity between different dark (gray) colored models, but only for the rest of the connections. The complete picture of the models used to construct the neuron-to-astrocyte interaction models is given in Supplementary Table S1. In addition, there was one model that did not explain the details of the model and is excluded from this evolutionary presentation

Astrocytes sense with their membrane mechanisms local and even distant environments, shown in in vitro cell cultures, ex vivo brain slices, and in vivo (Glaum et al., 1990; Dani et al., 1992; Porter & McCarthy, 1996; Hirase et al., 2004). Astrocytes have been shown to convert the signals they receive from neurons in local and more distant environments into Ca^2+^ excitability. In about half of the computational models, astrocytes were activated by released neurotransmitters; the neurotransmitter release was modeled according to several studies (see, e.g., Destexhe et al., 1994; Tsodyks et al., 1998; Terman et al., 2002) and the neurotransmitter’s impact on astrocytes was described similarly as in several studies (see, e.g., Nadkarni & Jung, 2005; Volman et al., 2007; De Pittà et al., 2009; Gordleeva et al., 2012; De Pittà & Brunel, 2016). The other half utilized different kinds of phenomenological transfer functions in neuron-to-astrocyte interactions. For example, presynaptic membrane potential ($V_{\textrm{m}}$) or postsynaptic 2-AG directly affected the astrocytic IP_3_ concentration ($V_{\textrm{m,pre}} \rightarrow \mathrm {[IP_{3}]_{ast}}$ or $\mathrm {[2\text {-}AG]_{post}} \rightarrow \mathrm {[IP_{3}]_{ast}}$) (Nadkarni & Jung, 2003; Wade et al., 2012) or other functions based on several previous studies were used (Kopell et al., 2000; Suffczynski et al., 2004; Postnov et al., 2007; Yao et al., 2011) (Fig. 5, Table 2, and Supplementary Tables S1–S3).

**Fig. 6:**
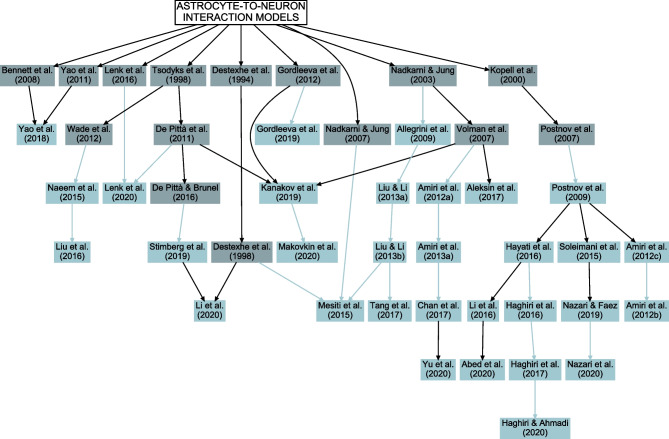
Evolution of astrocyte-to-neuron interactions in the neuron-astrocyte network models. The dark (gray) colored models represent a set of models that were utilized when building the astrocyte-to-neuron interaction models of the light (blue) colored neuron-astrocyte network models. The light (blue) colored arrows mean that the astrocyte-to-neuron interaction model above and the model below the arrow have equations that are the same. The black arrows mean that the model above is partly or completely used by the model below, but the equations are not the same. However, note that we did not classify the similarity between different dark (gray) colored models, but only for the rest of the connections. The complete picture of the models used to construct the astrocyte-to-neuron interaction models is given in Supplementary Table S1. In addition, there was one model that did not explain the details of the model and is excluded from this evolutionary presentation

Since astrocytes have been shown to release signaling molecules to the vascular and neuronal systems, they are now considered to have a more active role in different brain functions than previously thought. The Ca^2+^-dependent astrocytic release of gliotransmitters, such as glutamate, D-serine, and ATP, and different modulators is generally called gliotransmission (Parpura et al., 1994; Araque et al., 1999; Bezzi & Volterra, 2001; Parri et al., 2001). However, it is not yet known what the exact release mechanisms are in different astrocytic functions. It has been proposed that the release could occur through several different mechanisms, such as exocytotic release, diffusional release through membrane pores, transporter-mediated release, or vesicular release. Indeed, vesicle-type structures have been detected in astrocytes in vitro, but the exact molecular machinery for packing gliotransmitters into vesicles and releasing them has not yet been shown in vivo (see discussions in Fujita et al., 2014; Sloan & Barres, 2014). None of the neuron-astrocyte network models used a detailed astrocytic vesicle release model (Fig. 6, Table 2, and Supplementary Tables S1–S3) mainly because the exact mechanisms of gliotransmitter release is not yet known. However, about one third of the models included gliotransmitter (GT) release by modeling mostly extracellular glutamate ([Glu]_ext_), but Postnov et al. (2009), Yang and Yeo (2015), Li et al. (2016), Haghiri et al. (2017), Yao et al. (2018), and Gordleeva et al. (2019) included also extracellular ATP ([ATP]_ext_ or ATP_ext_) or D-serine (D-serine_ext_). Stimberg et al. (2019) and Li et al. (2020) used the extension of the model by Tsodyks et al. (1998) for astrocytic release of gliotransmitters based on previous studies (De Pittà et al., 2011; De Pittà & Brunel, 2016), and, in addition, Li et al. (2020) also used the previous studies by Destexhe et al. (1994, 1998). Rest of the models utilized different kinds of gliotransmitter functions based on previous studies (Bennett et al., 2008; Gordleeva et al., 2012; Wade et al., 2012), phenomenological transfer functions to mimic the effect of gliotransmission or exocytotic mechanisms to synaptic terminals, such as different currents depending on astrocytic Ca^2+^ (see, e.g., $I_{\textrm{astro}}$ developed in Nadkarni & Jung, 2003), astrocytic mediator abbreviated as $G_{\textrm{m}}$ ($I_{\textrm{ast}} = cG_{\textrm{m}}$ and $I_{\textrm{syn}} = (k-cG_{\textrm{m}})(z-z_0)$) developed by Postnov et al. (2007, 2009) and originating from the study by Kopell et al. (2000), phenomenological gating variable abbreviated as *f* ($I_{\textrm{ast}} = cf$) developed by Volman et al. (2007) and Amiri et al. (2012a), and other functions based on previous studies (Nadkarni & Jung, 2007; Yao et al., 2011; Amiri et al., 2013b; Lenk et al., 2016).

**Fig. 7:**
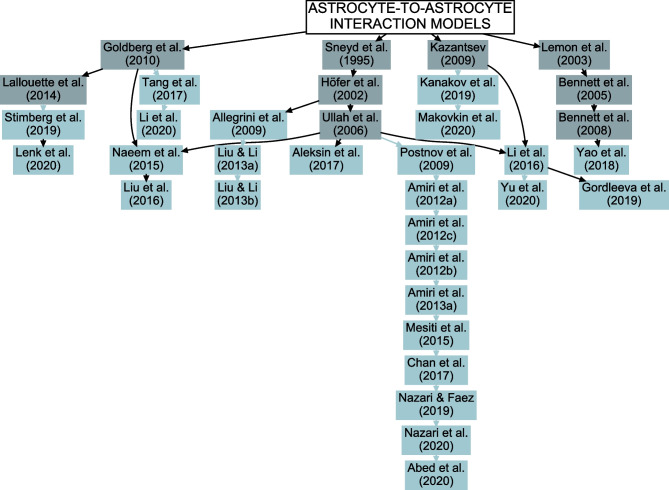
Evolution of astrocyte-to-astrocyte interactions in the neuron-astrocyte network models. The dark (gray) colored models represent a set of models that were utilized when building the astrocyte-to-astrocyte interaction models of the light (blue) colored neuron-astrocyte network models. The light (blue) colored arrows mean that the astrocyte-to-astrocyte interaction model above and the model below the arrow have equations that are the same. The black arrows mean that the model above is partly or completely used by the model below, but the equations are not the same. However, note that we did not classify the similarity between different dark (gray) colored models, but only for the rest of the connections. The complete picture of the models used to construct the astrocyte-to-astrocyte interaction models is given in Supplementary Table S1. In addition, there were six models that did not explain the details or did not model astrocyte-to-astrocyte interactions and are excluded from this evolutionary presentation

Astrocytes can be connected to each other through gap junctions that are composed of mainly connexin 43 hemichannels. The gap junction connections allow exchange of ions and molecules between astrocytes and can contribute to many astrocytic functions, such as Ca^2+^ waves, water transport, K^+^ buffering, control of vascular system, and even synaptic plasticity (Pannasch et al., 2011). In the neuron-astrocyte network models, astrocyte-to-astrocyte interactions were mostly implemented by gap junctions based on the earlier studies (Sneyd et al., 1995; Höfer et al., 2002; Ullah et al., 2006; Kazantsev, 2009; Goldberg et al., 2010; Lallouette et al., 2014), but also by diffusion in extracellular space (Lemon et al., 2003; Bennett et al., 2005, 2008) (Fig. 7, Table 2, and Supplementary Tables S1 and S3). Most models included gap junction signaling between astrocytes for IP_3_, and some also for Ca^2+^. A few models utilized extracellular diffusion of gliotransmitters or ions to activate either neurons or astrocytes (Postnov et al., 2009; Yang & Yeo, 2015; Li et al., 2016; Yao et al., 2018).

#### Spatial Organization and Structure of Interactions between Cells

Typically, when modeling populations of cells using network formalism, the number of modeled cells is large and statistical rules are used to decide which cells are allowed to interact. The resulting interaction scheme has a non-trivial structure, and this structure constrains the global dynamics and the functions of the model. In the previous section, we described all kinds of interactions considered in the analyzed models, within and between various neuronal types, between neurons and astrocytes, and within populations of astrocytes. Here we analyze the rules used to determine which cells are allowed to interact and how such rules constrain information flow in the models. Furthermore, we derive criteria for classification of interaction schemes and the steps to improve description of interaction schemes in this category of models.

As usual, we mapped interactions between any two populations of cells, $P_1$ and $P_2$, into a binary matrix $\Gamma (P_1, P_2) = \{\gamma _{ij} \}_{i \in P_1, j \in P_2}$, where each matrix entry $\gamma _{ij}$ maps the presence ($\gamma _{ij}=1$) or absence ($\gamma _{ij} =0$) of a directed interaction from the cell $i \in P_1$ to the cell $j \in P_2$. In a system containing *M* different cell types, the complete model is described by $$\left( {\begin{array}{c}M+1\\ 2\end{array}}\right)$$ binary matrices. In neuronal network models, where neurons interact through synaptic connections, this notation is called the *connectivity scheme*. Here, we considered diverse mechanisms of interactions between neurons and astrocytes, including the interactions through synaptic connections, extrasynaptic receptors, and gap junctions, so we opted to use a less constraining terminology *interaction scheme*. Interactions can be constrained by spatial location of the cells; thus, we also analyzed spatial arrangement of cells.

**Fig. 8:**
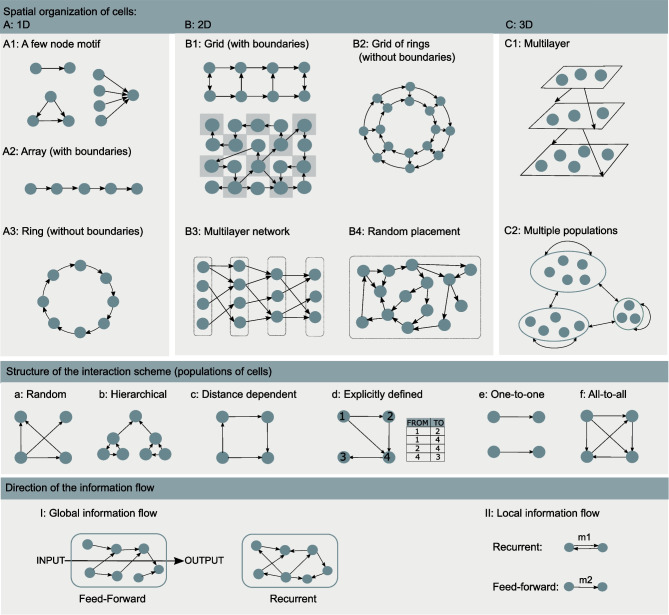
Illustration of the criteria developed to categorize network-level properties in the neuron-astrocyte network models. **Spatial organization of cells** can be **A)** 1D, **B)** 2D, or **C)** 3D. **1D** models can be **A1)** a few node motifs, **A2)** arrays (with boundary conditions), or **A3)** rings (arrays without boundary conditions). **2D** models can be **B1)** grids that impose rigid and regular spatial organizations and represent a 2D extension of 1D arrays, **B2)** grids of rings that remove boundary conditions from the grid structure and represent a 2D extension of 1D rings, **B3)** multilayer networks where cells are arranged into distinct 1D layers and interactions are defined within and across the layers, or **B4)** random placements where coordinates of the cells are randomly selected within the 2D space. **3D** models consist of two categories: **C1)** multilayer 3D models and **C2)** models of multiple populations that imply 3D structure as they represent different brain areas. **Structure of the interaction scheme** can belong to one of six categories: **a)** random interaction scheme where each pair of cells interacts with equal (fixed) probability, **b)** hierarchical interaction scheme where cells are divided into hierarchy levels and interactions are defined within and across hierarchies, **c)** distance-dependent interaction scheme where coordinates are assigned to each cell, and probability of interaction increases with the proximity of cells, **d)** explicitly defined scheme where interactions are established deterministically between the selected cells, **e)** one-to-one interaction scheme where each cell can interact with only one other cell, or **f)** all-to-all interaction scheme where interactions are possible between each pair of cells. **Direction of the information flow** is defined **I)** globally for all cells in the model or **II)** locally between pairs of interacting cells. Interactions can be feed-forward, if the input and output of the interaction scheme are well defined, or recurrent, if the interaction scheme allows loops and feedback between cells

We developed the following criteria to categorize the models: **(i)** spatial organization of cells in the model (illustrated in Fig. 8A–C), **(ii)** structure of the interaction scheme (Fig. 8a–f), and **(iii)** direction of the information flow in the scheme (Fig. 8I–II).

Cells can be distributed in one-dimensional (1D), 2D, or 3D space. In the most constrained case, in **1D** models, we identified three different arrangements: few node motifs (Fig. 8A1), an array (Fig. 8A2), and a ring structure (Fig. 8A3) that represents an array without boundaries. The few node motifs found in the analyzed studies were the minimal scheme of two cells with a single interaction, the three node motifs, and convergent inputs from several cells to a single cell. **2D** spatial arrangements were the most numerous in the studies and we divided them into four categories: regular grids with boundaries (Fig. 8B1) or grids of rings without boundaries (Fig. 8B2), 2D multilayer networks (Fig. 8B3), and models with randomly distributed cells in the 2D space (Fig. 8B4). We identified two types of **3D** models, 3D multilayer networks (Fig. 8C1) and models of connected populations (Fig. 8C2), where each population might belong to a different brain region. Classifying these latter models as 3D is somewhat debatable. These models did not explicitly incorporate the notion of space, but because they aimed at representing intrinsically 3D brain structures, we opted to classify them as 3D.

We distinguished six categories of models according to the structure of the interaction scheme which are arranged from the most irregular (Fig. 8a) to the most regular (Fig. 8f) in Fig. 8a–f. The random (Erdős-Rényi) interaction scheme allows each pair of cells to interact with equal probability, regardless of the spatial location of the cells (Fig. 8a). Thus, the number of inputs to each cell is a binary distributed random variable, and the uncertainty about interactions is maximal. In this case, the spatial organization of cells does not affect the interaction scheme, so some of the studies omitted this information which we needed to categorize models according to our criteria (i). On the other hand, Li et al. (2020) explicitly specified 2D random placement of cells. We opted to categorize all models for which spatial organization was not defined by the authors as 2D random placement (Fig. 8B4). Random distribution indicates that precise location of cells is not important in this interaction scheme.

Some of the analyzed studies used the hierarchical interaction scheme (Fig. 8b) with cells organized into hierarchical levels and interactions defined within and between those levels. In the distance-dependent interaction scheme, physical location is defined for each cell and physical distance between cells determines the probability of interaction (Fig. 8c). Examples of such studies are, for example, the models by Allegrini et al. (2009), Postnov et al. (2009), and Lenk et al. (2020). Some studies defined explicitly which cells were allowed to interact (Fig. 8d), these included models with 2D grid organization where cells interacted with some or all their closest neighbors (for astrocytes see, e.g., Amiri et al., 2012a; Kanakov et al., 2019). We included two categories that are special cases of the explicitly defined interaction scheme: one-to-one interactions (Fig. 8e) and all-to-all interactions (Fig. 8f). The all-to-all interaction scheme, where each cell from the population $P_1$ interacts with each cell from the population $P_2$, removes every uncertainty about the interaction scheme.

We distinguished two types of models according to ‘direction of the information flow’. In feed-forward interaction schemes, the input and output of a model can be easily identified, and the information propagates from the input to the output. In recurrent interaction schemes, the input and output are not obvious, and loops are allowed between cells. We used this ‘global’ definition to characterize the overall information flow in the entire network model when all cell types and all interaction types were included (see ‘Global’ in Table 3 and for illustration in Fig. 8I). Here we ignored local recurrence, for example between astrocytes and neurons, and categorized models according to the overall information propagation through the model. If it was possible to clearly identify sources and sinks of the information, the model was classified as feed-forward (see, e.g., Amiri et al., 2012a; Liu & Li, 2013a; Haghiri et al., 2016; Nazari et al., 2020), otherwise, the model was classified as recurrent (see, e.g., Tang et al., 2017; Stimberg et al., 2019; Li et al., 2020).

We also examined local recurrence in interactions between pairs of cells. If there was a finite probability that a pair of cells formed a loop (the motif m1 illustrated in Fig. 8II), we considered their interaction to be recurrent. Otherwise, if only motif m2 was possible, we categorized this as a feed-forward interaction. The interaction between two populations $P_1$ and $P_2$ was feed-forward if all cells from $P_1$ formed feed-forward interactions with all cells from $P_2$, otherwise the interaction was recurrent. In most of the considered studies, neurons were coupled through a synapse that allowed exchange strictly from the presynaptic to the postsynaptic neuron (but see, e.g., Naeem et al. (2015), as an example of a recurrent synapse). However, if a pair of neurons had a finite probability of forming a loop m1 through multiple synapses, for example in a random interaction scheme, recurrent information flow was possible. Astrocytes may interact through gap junctions, which is by definition a recurrent interaction (but see, e.g., Yao et al. (2018), as an example of interactions mediated by ion channels and extracellular ionic concentrations). Interactions between neurons and astrocytes are somewhat more complex. An example of a recurrent neuron-astrocyte interaction is the case where the presynaptic neuron releases neurotransmitters that affect astrocytic Ca^2+^, and in return, the astrocyte modulates release from the presynaptic terminal. An example of a feed-forward interaction is a commonly adopted model where the presynaptically released glutamate induces Ca^2+^ transients in astrocytes, which in response affects the excitability of a postsynaptic cell.

**Fig. 9:**
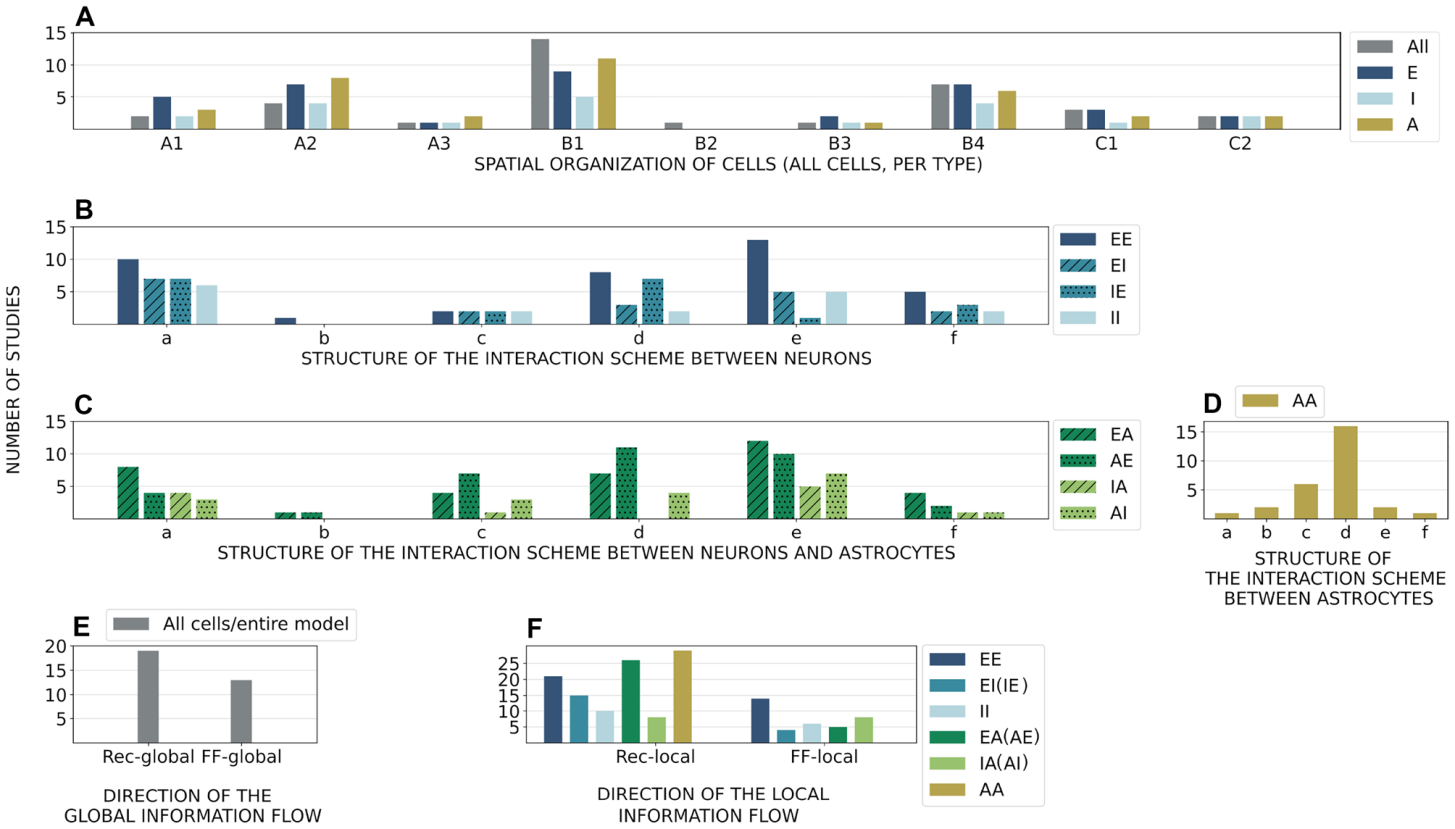
Prevalence of the different categories, described in Fig. 8, in the analyzed neuron-astrocyte network models. **A) Spatial organization of cells** across studies when analyzing all cells together (gray) and separately for excitatory neurons (dark blue), inhibitory neurons (light blue), and astrocytes (yellow). Naming of the categories: a few node motif (A1), 1D array with boundaries (A2), 1D ring without boundaries (A3), 2D grid with boundaries (B1), 2D grid of rings without boundaries (B2), 2D multilayer network (B3), random placement of cells in 2D (B4), 3D multilayer network (C1), and multiple populations possibly in different brain regions (C2). **B) Structure of the interaction scheme** between neuronal types, between excitatory neurons (EE, dark blue), from excitatory to inhibitory neurons (EI, blue with hatch pattern), from inhibitory to excitatory neurons (IE, blue with dotted pattern), and between inhibitory neurons (II, light blue). **C)** Structure of the interaction scheme between neurons and astrocytes, from excitatory neurons to astrocytes (EA, dark green with hatch pattern), from astrocytes to excitatory neurons (AE, dark green with dotted pattern), from inhibitory neurons to astrocytes (IA, light green with hatched pattern), and from astrocytes to inhibitory neurons (AI, light green with dotted pattern). **D)** Structure of the interaction scheme between astrocytes (yellow). Categories: random interaction scheme (a), hierarchical interaction scheme (b), distance-dependent interaction scheme (c), explicitly defined interaction scheme (d), one-to-one interaction scheme (e), and all-to-all interaction scheme (f). **E) Direction of the global information flow** considers the entire model, its inputs and outputs. Categories: globally recurrent model (Rec-global) and globally feed-forward model (FF-global). **F) Direction of the local information flow** characterizes interactions between pairs of cells; both interacting cells are excitatory neurons (EE, dark blue), both cells are inhibitory neurons (II, light blue), interaction between one excitatory and one inhibitory neuron (both directions EI(IE) considered, blue), interaction between excitatory neuron and astrocyte (EA(AE), dark green), interaction between inhibitory neuron and astrocyte (IA(AI), light green), and interaction between pairs of astrocytes (AA, yellow)

Using these criteria, we characterized interactions in each of the analyzed studies and present the results in Table 3 and Fig. 9. We first considered interactions between all modeled cells (see ‘All cells’ in Table 3 and Fig. 9), and then interactions between each pair of modeled cellular populations $P_1$ and $P_2$, including the case where $P_1 = P_2$ (see, e.g., EE as an interaction between excitatory cells, EA as an interaction from excitatory neurons to astrocytes, and EA(AE) as an interaction between excitatory neurons and astrocytes where both directions are considered). Table 3 presents detailed characterization of each analyzed study for each cell and interaction type according to the criteria (i)-(iii), and Fig. 9 summarizes these results. In addition, Table 2 presents the number of interactions per cell and interaction type, for example the number of interactions from an excitatory neuron to the rest of excitatory population, from an astrocyte to the excitatory neurons, and from an astrocyte to the inhibitory neurons; these interactions can substantially differ within the same model.

We first characterized spatial organization of all cells taken together; we counted how many times each category (Fig. 8A1–C2) appeared in the considered studies and under ‘All cells’ in the second column of Table 3. If a study presented two models organized according to two distinct categories, then the number of occurrences of both categories was increased by one. If a study presented two models falling into the same category, then occurrence of the category was increased only once. Such counting of categories is also done when considering cell types separately. The results are shown in Fig. 9A. The most represented spatial organizations were B1 (2D grid with boundaries) and B4 (2D random placement of cells). The categories B1 in 2D and A2 (1D array with boundaries) in 1D included examples of regular interaction schemes constructed to support feed-forward information transfer (see, e.g., Amiri et al., 2012a; Haghiri et al., 2017). As such, they are useful for theoretical studies of information propagation and synchronization, and to examine how astrocytes support these functions. The B1 scheme was commonly used for astrocytes, to represent (in a reduced way) how astrocytes parcellate 2D space into non-overlapping domains. The category B4, random placement of cells, was frequently used to model neural populations, particularly when the details of their spatial organization were not known or not relevant for the study.

Next, we examined the structure of the interaction scheme and counted the occurrence of the six categories, illustrated in Fig. 8a–f, for different interaction types (EE, EI, EA, etc.). The results are shown in Fig. 9B for neuronal interactions, in Fig. 9C for the interactions between neurons and astrocytes, and in Fig. 9D for the interactions between astrocytes. Random interaction schemes (category a) were a relatively common choice when modeling neuronal interactions, which reflects common use of this interaction scheme in neuronal models in general. One-to-one interaction schemes (category e) were particularly frequent between excitatory neurons, and they often appeared in models with 2D grid organization. Interactions between excitatory neurons and astrocytes were often explicitly defined (d) or one-to-one schemes (e). One-to-one schemes were used when astrocytes were modeled as a single compartment that affected a specific, explicitly defined, single synapse and either the presynaptic or the postsynaptic neuron. When the astrocytic compartment interacted with both pre- and postsynaptic neurons, or with more than one explicitly determined synapse, we categorized these schemes as explicitly defined. Interactions between astrocytes and inhibitory neurons were less common in the considered models (in general, the underlying biological mechanisms are less understood) which results in lower counts for IA and AI interactions, compared to EA and AE interactions, in all six categories in Fig. 9C. Finally, astrocytic interactions were mainly between physically close cells, given either as explicitly defined interaction schemes (d) or as distance-dependent interaction schemes (c).

Recurrent interaction schemes prevailed when considering (global) direction of the information flow in the entire model, between all model cells (Fig. 9E). Local recurrent schemes were also more common for all types of interactions except between inhibitory neurons and astrocytes (Fig. 9F). Inhibitory presynaptic neurons rarely affected astrocytes in the studied models; their interactions were mostly feed-forward from astrocytes to neurons. Astrocytes were allowed to interact with other astrocytes mainly through gap junctions (or extracellular space, see, e.g., Yao et al., 2018) which were always recurrent interactions.

### Neural Functions Studied with Models

Experimental evidence has been accumulating on the roles of astrocytes in different brain functions, such as neuronal excitability, synaptic transmission and plasticity, as well as in higher cognitive functions related to initiation, maintenance, and consolidation of memories (Volterra et al., 2014; Bazargani & Attwell, 2016; Magistretti & Allaman, 2018). Understanding glial mechanisms and their contributions to various brain functions can benefit from computational modeling and in silico experiments. Here, we summarize different brain functions that were addressed in the considered computational studies using neuron-astrocyte network models (Table 1).

As described earlier, our analysis included only network models, thus models including some form of signal or information transfer, with sufficiently detailed astrocytic Ca^2+^ dynamics. Among the analyzed models, several of them entirely focused on explaining astrocytic Ca^2+^ dynamics (we categorized them as ‘Ca^2+^’ in Table 1) or signal or information transfer (Sgn./Inf.). Others described and analyzed various additional properties and functions of neuron-astrocyte circuits including excitatory-inhibitory balance (E-I balance), synchronization (Sync.), synaptic plasticity (Plast.), or hyperexcitability (Hyper.). Finally, some models were built with engineering goals in mind, to test the capacity of neuron-astrocyte systems as classifiers (Classif.) or to develop neuro- and gliomorphic hardware (HW).

First, we will address the studies that modeled Ca^2+^ dynamics. Synaptically released glutamate can activate astrocytes by increasing Ca^2+^ concentration locally and by inducing Ca^2+^ wave propagation between astrocytes in vitro (Cornell-Bell et al., 1990; Charles et al., 1991; Dani et al., 1992; Newman & Zahs, 1997). Recent studies have also observed astrocytic Ca^2+^ oscillations and signaling in vivo (see, e.g., Nimmerjahn et al., 2009; Ding et al., 2013; Paukert et al., 2014; Srinivasan et al., 2015; Poskanzer & Yuste, 2016; Agarwal et al., 2017; Stobart et al., 2018a; Lines et al., 2020). Specifically, it has been shown that the astrocytic Ca^2+^ oscillations in the soma are different than in the processes (Otsu et al., 2015; Stobart et al., 2018b), and, interestingly, Ca^2+^ oscillations have been shown to be diverse also in distinct regions of the processes (Arizono et al., 2020). Various mechanisms have been suggested to contribute to these intracellular Ca^2+^ oscillations, such as G-protein coupled receptors (see, e.g., Savtchouk & Volterra, 2018), transient receptor potential channels (see, e.g., Shigetomi et al., 2013), Na^+^/Ca^2+^ exchangers (see, e.g., Rojas et al., 2007), IP_3_Rs on the ER membrane (see, e.g., Srinivasan et al., 2015; Sherwood et al., 2017), and mechanisms related to mitochondria (see, e.g., Agarwal et al., 2017). Possible mechanisms responsible for Ca^2+^ wave propagation between astrocytes are gap junctions and extracellular diffusion of ATP (Fujii et al., 2017). Most of the neuron-astrocyte network models focused on the mechanisms related to ER, thus IP_3_Rs and SERCA pumps, as well as on gap junctions. Four studies included more detailed models of astrocytic Ca^2+^ dynamics (Postnov et al., 2009; Liu & Li, 2013b; Mesiti et al., 2015; Gordleeva et al., 2019). For example, Gordleeva et al. (2019) presented multicompartmental models for two astrocytes, each composed of a somatic compartment and 14 processes consisting of, in total, 52 compartments. In their model, neuron-activated astrocyte processes exhibited Ca^2+^ signals that propagated to the soma, and backwards from the soma to the processes. Distal processes had more frequent Ca^2+^ signals than the proximal processes and the soma. The study demonstrated astrocytic role in modulation of presynaptic release and in coordinating activity of multiple synapses. In the light of new evidence of diverse astrocytic Ca^2+^ mechanisms, the field needs to develop more detailed data-based models of astrocytic Ca^2+^ signaling that include also other mechanisms in addition to the ER, for the neuron-astrocyte network models.

Neuronal and astroglial cells interact through release and uptake of various ions and molecules that are mediated by complex cellular mechanisms. These molecular and ionic mechanisms further facilitate and modulate the action potential -mediated signal and information transfer between neuronal cells. Signal transfer between neurons is realized via neurotransmission or synaptic transmission, while astrocytes exchange signals with neurons via gliotransmission. Astroglial cells have been shown to actively modulate signal transmission between neurons. In the developing central nervous systems, astrocytes support neuronal interaction by contributing to formation of excitatory synapses and synaptic connectivity (Allen & Eroglu, 2017). Astrocytes have also been shown to modulate information processing in mature brain circuits and influence animal behavior (Pannasch & Rouach, 2013; Oliveira et al., 2015; Chever et al., 2016; Poskanzer & Yuste, 2016; Lines et al., 2020). One basic, well-known mechanism is the transformation of excessive glutamate to glutamine: after presynaptic terminal releases glutamate, astrocytes can take up the excess glutamate, transform glutamate into glutamine, and release glutamine into the extracellular space which is followed by the presynaptic terminal metabolizing glutamine back to glutamate (Danbolt, 2001). None of the analyzed neuron-astrocyte network models, however, studied this phenomenon. Nevertheless, eight of the models, equipped by diverse mechanisms and interaction schemes related to neurotransmission and gliotransmission, were primarily focused on studying signal or information transfer in neuron-astrocyte networks (Liu & Li, 2013a, b; Yang & Yeo, 2015; Li et al., 2016; Kanakov et al., 2019; Nazari & Faez, 2019; Abed et al., 2020; Nazari et al., 2020).

Next, we explain the additional neural functions that the models addressed. Astrocytes possess molecular machinery that allows them to modulate both glutamatergic and GABAergic transmission (Losi et al., 2014; Bazargani & Attwell, 2016; Perea et al., 2016; Mederos & Perea, 2019), and thus potentially affect the excitatory-inhibitory balance in brain circuits. Two of the models studied excitatory-inhibitory balance (Postnov et al., 2009; Li et al., 2020). Li et al. (2020) explored how the mechanisms of neuron-astrocyte interactions affect excitation-inhibition balance. Li et al. (2020) showed with their model that the higher the exogenous GABA stimulus, the lower the synaptically released glutamate and the earlier and higher the release of glutamate from astrocytes. Moreover, the release of glutamate from astrocytes had an excitatory impact on synaptic release of glutamate, thus counteracting the inhibitory effect of GABA on synaptic release of glutamate.

Synchronization, an emergence of coordinated activity in a group of interacting units (e.g., cells and brain areas), plays an important role in information transfer and brain computations. It has been studied at all levels of brain organization, including micro-, meso-, and macroscale levels. Earlier studies explored the impact of neuronal excitability, inhibitory and excitatory synaptic transmission, as well as the structure of neuronal network connectivity on synchronization (see, e.g., Mäki-Marttunen et al., 2013). Recently, the astrocytic contribution to cortical network synchronization in vivo has also been shown (Takata et al., 2011; Chen et al., 2012; Paukert et al., 2014; Perea et al., 2014). More than half of the models (18/32; see categorization in Table 1) included in this study addressed the role of astrocytic mechanisms in emergence of global synchronization. However, experimental literature on astrocytic mechanisms that contribute to network synchronization is somewhat scarce. Computational in silico experiments may help steer the future exploration of putative mechanisms both in vitro and in vivo.

Synaptic plasticity refers to an activity-dependent modification of the strength or efficiency of synaptic transmission that has been suggested to play an important role in the brain’s ability to incorporate transient experiences into long-lasting memories. Synaptic plasticity is also shown to play a key role in the early development of neural circuits (Allen & Eroglu, 2017), and there is evidence that impaired synaptic plasticity mechanisms contribute to neuropsychiatric disorders. To date, multiple forms, functions, and mechanisms have been presented for synaptic plasticity. There is growing evidence that astrocytes may be involved not only in short-term plasticity (Araque et al., 2001; Haydon, 2001), but also in long-term plasticity (Perea & Araque, 2007; Min & Nevian, 2012; Sherwood et al., 2017). Modeling-wise synaptic plasticity has been studied mainly with neuron-astrocyte synapse models and these studies indicate that complex cellular- and molecular-level mechanisms are involved (see, e.g., Tewari & Majumdar, 2012; Manninen et al., 2020). There exist fewer studies addressing the role of astrocytes in synaptic plasticity in networks and brain circuits. However, five studies using neuron-astrocyte network models demonstrated some form of synaptic plasticity (Mesiti et al., 2015; Naeem et al., 2015; Hayati et al., 2016; Aleksin et al., 2017; Gordleeva et al., 2019).

Hyperexcitability is a state of the brain activity where firing of neurons is disturbed, and neuronal networks become excessively excitable. Pathophysiological hyperexcitability is observed in many neurological disorders, including epilepsy, migraine, tinnitus, neurodegeneration, and neurodevelopmental disorders. The mechanisms underlying hyperexcitability are not fully understood. Several molecular and cellular mechanisms, including defects in expression or functional regulation of ion channels and changes in excitatory and inhibitory synaptic activity, have been commonly attributed to hyperexcitability. Glial cells have also been linked with hyperexcitability and, as an example, astrocytes from epileptic brain show abnormal patterns of intracellular Ca^2+^ signals (see for a review, e.g., Carmignoto & Haydon, 2012; Shigetomi et al., 2019). In addition, astrocytes can help in preventing neuronal networks from becoming over-excited by clearing excess extracellular K^+^ and other ions from the extracellular space in the central nervous system (Orkand et al., 1966). Three models in our analysis addressed the role of astrocytes in neuronal network hyperexcitability (Amiri et al., 2012b; Tang et al., 2017; Yao et al., 2018). Many of the models, studying either synchronization or hyperexcitability, also addressed epilepsy (see, e.g., Amiri et al., 2012a, b, c; Yu et al., 2020) and formation of seizures (see, e.g., Tang et al., 2017).

In addition, two of the models applied biologically inspired models to visual classification problems (Nazari & Faez, 2019; Nazari et al., 2020) and five models were built for testing hardware implementation (Soleimani et al., 2015; Haghiri et al., 2016, 2017; Hayati et al., 2016; Liu et al., 2016).

## Discussion

We analyzed altogether 32 neuron-astrocyte network models published by 2020 that fulfilled the following conditions: **(1)** the models represent astrocytic Ca^2+^ dynamics, an assumed key messaging system of astrocytes, explicitly, **(2)** the models are considered networks; thus they include at least two neurons and two astrocytes, and **(3)** the interactions between neurons and astrocytes are bidirectional. We first carefully screened all model equations and derived evolutionary trees of neuronal and astrocytic cell models as well as of cellular interaction models, thus the representations of exchanges between different neurons and astrocytes in the model, used in the 32 considered publications. We then focused on network interactions, performed a detailed comparative analysis of network structure and interaction schemes in the models, defined categories of models according to interaction schemes, and computed frequency of each category in the considered studies. To the best of our knowledge, this is the first time such a detailed analysis of the computational network models involving neuron-astrocyte interactions has been done. The aim of our study is to conceptualize modeling of neuron-astrocyte networks and facilitate development of models, methods, and tools necessary to advance this category of computational models.

In recent years, the interest in computational modeling of neuron-astrocyte networks started to surpass the focus on single astrocytes or populations of astrocytes (see the trend in Fig. 1). These neuron-astrocyte network models were often constructed to study typical network properties such as synchronization and signal or information transfer (Table 1), while some were developed to test potential for engineering applications such as classification algorithms or hardware implementations. About one third of the analyzed models were not specialized for any brain area but were constructed as generic population-level models. The size of the constructed network models ranged from a few cell models to thousands of cells. The largest among the network models managed the computational burden by adopting low-dimensional and computationally light single neuron models, whereas the smaller network models allowed more detailed neuron models (Hodgkin & Huxley, 1952; Pinsky & Rinzel, 1994) and astrocyte models (see, e.g., Gordleeva et al., 2019) (Figs. 2 and 3). The interactions between neurons were represented as conventional synaptic models, that might include the presynaptic short-term dynamics, dynamics of postsynaptic receptors, and in a few cases also the long-term plasticity. Modeling synaptic inputs as excitatory or inhibitory currents to the cell membrane (models with postsynaptic current in Fig. 4 based on, e.g., Izhikevich, 2003; Volman et al., 2007) or as conductivity changes in response to presynaptic release of neurotransmitters (models with postsynaptic conductance in Fig. 4 based on, e.g., Jahr & Stevens, 1990; Destexhe et al., 1998; Latham et al., 2000; Dayan & Abbott, 2001; Gerstner & Kistler, 2002) were equally represented. When studying the interactions between neurons and astrocytes, we realized that half of all models explicitly represented neurotransmitters and their impact on astrocytes, while third of the models explicitly represented gliotransmitters and their impact on neurons (Table 2). The rest of the models used various kinds of phenomenological transfer functions between neurons and astrocytes (see Figs. 5 and 6, Table 2, and, e.g., Kopell et al., 2000; Nadkarni & Jung, 2003; Postnov et al., 2007; Volman et al., 2007; Wade et al., 2012). The interactions between astrocytes were mainly implemented by mathematical equations representing the functions of gap junctions and diffusion in extracellular space (Table 2 and Fig. 7). We found out that many of the models included similar cellular, synaptic, and non-synaptic mechanisms; however, several different spatial organizations of cells and structures of interaction schemes were implemented as shown in Figs. 8 and 9.

Lack of experimental data affects modeling of cellular-level details of astrocytic Ca^2+^ dynamics. The functions of astrocytes have been studied in three phases (Bazargani & Attwell, 2016), first investigating the functional properties and the mechanisms behind them in cell cultures, then in brain slices, and now also in vivo. Ca^2+^ signaling is assumed to be one of the key mechanisms mediating signaling and information transfer in astrocytes and it is represented in all models considered here. All these models rely on earlier studies that reconstructed Ca^2+^ dynamics from in vitro cell cultures, that did not always include astrocytes, or from isolated oocytes. Recent studies have found that Ca^2+^ behaves differently in the soma of an astrocyte compared to astrocytic perisynaptic processes and the mechanisms involved are complex (Otsu et al., 2015; Srinivasan et al., 2015; Sherwood et al., 2017; Stobart et al., 2018b; Arizono et al., 2020). However, it is not yet fully understood which of the astrocytic mechanisms (e.g., cell membrane, ER, and mitochondrial mechanisms) contribute to the Ca^2+^ data measured from different astrocytic regions and how these astrocytic mechanisms interact with neurons. Recent evidence has shown that transient opening of mitochondrial pores induces Ca^2+^ transients in astrocyte processes (Agarwal et al., 2017) and astroglial ER-mitochondria Ca^2+^ transfer mediates synaptic integration (Serrat et al., 2021). In our previous studies, we have systematically categorized computational astrocyte models based on the mechanisms modeled (Manninen et al., 2018b, 2019). The most recently published single astrocyte models mostly consider the Ca^2+^ mechanisms of the ER and cell membrane (Taheri et al., 2017; Cresswell-Clay et al., 2018; Savtchenko et al., 2018; Denizot et al., 2019, 2022; Wu et al., 2019), but there are studies in which mechanisms related to, for example, mitochondria have been modeled (Diekman et al., 2013; Komin et al., 2015). Many of these recent single astrocyte models are multicompartmental representing the whole-cell morphology either as a simple (Cresswell-Clay et al., 2018) or detailed (Savtchenko et al., 2018) way or representing a part of the cell, such as a branchlet (Denizot et al., 2022). The neural network models analyzed in this study generally used only single-compartmental astrocyte models with ER- and cell membrane-induced Ca^2+^ signaling. In addition, only two of the network models actively used new experimental data when building and validating their models (Amiri et al., 2013a; Chan et al., 2017) and the data was measured only from neurons. Future large-scale astrocyte projects will hopefully bring better understanding of which Ca^2+^ mechanisms are important in different regions of astrocytes and in different brain areas. With this information, we can build accurate data-based single-cell models of astrocytes from which we can develop biophysically informed, computationally light models of astrocytes for neuron-astrocyte network simulations.

Reconstructions of network-level properties are also impaired by lack of experimental data. Definition of a network model requires specification of dynamics for each cell type as well as specifications of external inputs, spatial organization of cells, and interaction schemes that determine which cells in the model can interact. Based on our analysis, two common strategies were used to circumvent the obstacle of not having experimental data for defining the spatial organization of cells and the interaction schemes between cells in the analyzed network models – some of the studies adopted purely theoretical, well-defined interaction schemes, such as 1D and 2D rings and grids, that supported analysis of specific functions like information transfer or synchronization, while others opted for random interaction schemes and random placement of cells that minimized the assumptions and free parameters needed to construct the model (Figs. 8 and 9A-D). Most of the studies incorporated some knowledge about astrocytic domain organization in vivo. Astrocytes parcellate tissue into non-overlapping domains (see, e.g., Oberheim et al., 2009) which was often modeled as 2D grid of astrocytes (Fig. 9A). Interactions between astrocytes can happen at the border of their domains, so in the models, astrocytes often interacted only with the closest neighbors through recurrent gap junctions (Fig. 9D). According to anatomical studies, astrocytes are situated close to synapses (see, e.g., Oberheim et al., 2009) and have been shown to modulate synapses within their domains during development (Perea & Araque, 2007; Takata et al., 2011; Min & Nevian, 2012; Navarrete et al., 2012; Petrelli et al., 2020). Due to this, neuron-astrocyte interactions in the models were mostly local, categorized as distance dependent, explicitly defined, or one-to-one in our study (Fig. 9C). In fact, many studies modeled individual astrocytic compartments rather than the entire cells, often a single compartment per one or two synapses which resulted in high occurrence of one-to-one or few-to-few interactions between neurons and astrocytes (Fig. 9C). Recently, studies are starting to provide more detailed morphometric data on neuron-astrocyte circuits (see, e.g., Calì et al., 2019; Kikuchi et al., 2020) and reconstructions based on this data are starting to be published (Zisis et al., 2021). Future studies in this direction will provide new information on neuron-astrocyte network structures and interaction schemes, increase statistical significance of data extracted from experimental measurements, provide better characterization of number and type of different cells and their synaptic and other interactions, quantify their spatial organization within domains and layers, and highlight differences between brain regions. This will lead to constructing more biologically realistic large-scale computational neuron-astrocyte network models and facilitate exploring the role of astrocytes in brain functions using computational tools together with experimental methods.

Replicating the previously published simulation results with existing model implementations can be time-consuming because of, for example, changes in the simulation tool versions or needed software packages, but even more tedious is trying to implement published models based on the information in the original articles (Manninen et al., 2017, 2018a, 2019; Rougier et al., 2017). This is a big challenge in all areas of computational sciences (Baker, 2016; Munafò et al., 2017) and among the neuron-astrocyte network models. Of the 32 models, implementations of only two models were easily found online (Aleksin et al., 2017; Stimberg et al., 2019) and only seven named the programming language or simulation tool used (Table 1). Thus, a complete reimplementation using the provided mathematical equations and other model details is required to further study and analyze most of the presented models. However, incomplete specification of the model details and interaction schemes, that we sometimes found among the studied models, leads to difficulties in interpretation of the results, undermines their reproducibility, and complicates their development further. For example, a study could present the interaction scheme as a textual description leaving the ambiguity which cells can interact. Other studies may incorporate interaction schemes into equations, by specifying indices of the interacting cells; however, the description might not be consistent, and ambiguities might remain. In addition, how these models evolved from each other was often difficult to interpret because usually a maximum of one reference was given for every equation or parameter value which was often different from the original publication presenting that equation or parameter value. The interoperability between simulation tools also poses a possible challenge. Not all simulation tools have the same functionality, so mechanisms implemented in one tool do not always guarantee that they can be implemented the same way in another tool.

The considered computational modeling efforts represent the first steps towards building more biologically realistic neuron-astrocyte network models. While advances in collecting experimental data, integrating these data into computational models, additional specialized simulators, model analysis and model fitting tools, as well as new models based on in vivo recordings in different brain regions are needed, the usefulness of these early models is evident and their analysis important. Reduced models can guide intuition about network interactions, global dynamics, and network functions. They can also aid in developing and testing model components, such as neuronal and glial cellular-level models and interaction mechanisms including synaptic mechanisms, that are later used in larger and more biologically realistic models. Reduced models help to define benchmarks for developing standardized, open-access tools for implementation, simulation, and analysis of computational models. Finally, they motivate and facilitate development of new technologies.

Advances in understanding astrocytic mechanisms, their interactions with neuronal cells, and their contributions to behaviorally relevant brain functions have inspired a new class of neuromorphic solutions. These solutions can be divided into two categories – efficient hardware implementations of neuron-astrocyte circuits, and neuro-glio-inspired algorithms for artificial intelligence and robotics. New hardware implementations were proposed by Soleimani et al. (2015), Haghiri et al. (2016, 2017), Hayati et al. (2016), and Liu et al. (2016), while the studies by Nazari and Faez (2019) and Nazari et al. (2020) employed a recurrent neuron-astrocyte network to solve a classification task. Several other studies, that were not included in our analysis, contributed important neuromorphic solutions. The study by Irizarry-Valle and Parker (2015) proposed one of the first specialized hardware implementations of neuron-astrocyte circuits with astrocytes that sense synaptically released glutamate and in response modulate neuronal excitability. Tang et al. (2019) implemented neuron-astrocyte circuits in a general-purpose neuromorphic system, the Loihi chip (Davies et al., 2018), explored the astrocyte-mediated plasticity mechanisms, namely the astrocyte-induced heterosynaptic plasticity and the bidirectional homeostatic plasticity, and demonstrated how these mechanisms contribute to maintaining the optimal population activity regime. An extensive review of neuromorphic hardware by Schuman et al. (2017) also presented a summary of astrocyte-inspired hardware solutions. Porto-Pazos et al. (2011), Mesejo et al. (2015), and Rastogi et al. (2021) demonstrated how various mechanisms of neuron-astrocyte interaction can be used to improve performance in classification tasks. Finally, neuron-astrocyte circuits for robotic control were proposed by Liu et al. (2019) and Polykretis et al. (2020). The here discussed neuromorphic solutions based on neuron-astrocyte circuits demonstrated efficient hardware implementations and potential for engineering applications. Further advances in understanding astrocytic functions through experimental work and computational modeling can inspire new tools and algorithms (see, e.g., computational modeling of astrocytic contribution to working memory in Gordleeva et al., 2021; Tsybina et al., 2022).

To further advance the research field, we formulate a list of guidelines that need to be considered when developing neuron-astrocyte network models. First, collecting extensive experimental imaging data in vivo for public databases related to neuron and astrocyte morphologies, neuronal, astrocytic, and vascular tissue structures, astrocytic and neuronal Ca^2+^ dynamics, in addition to neuronal electrophysiological recordings, will facilitate data-driven modeling approaches. Second, we need to better understand the differences between in vitro and in vivo data and which mechanisms are involved in astrocytic interactions with its environment and proximal cells in different brain areas and across different astrocytic regions, including soma, main processes, perisynaptic processes, and perivascular endfeet. Third, all model details, including the network structure, number of cells, interaction scheme, and all equations, initial values, and parameter values should be clearly given. Fourth, description of the interaction schemes should be fully integrated into model equations and the authors should verify that the interaction schemes can be reconstructed from the equations alone. Fifth, the use of a pre-defined format for description of model components and interaction schemes is highly recommended and serves as a reminder of all model components that need to be specified; for this, the formats proposed for description of networks of neurons (Nordlie et al., 2009) and for connectivity schemes between neurons (Senk et al., 2022) can be extended for neuron-astrocyte networks as formulated in our study. Sixth, hypotheses tested in the simulations should be clearly stated (or, if the modeling work is purely data-based, it should be stated, see, e.g., Eriksson et al., 2022). Seventh, model implementations should be openly available in model databases with well-documented codes and explanations on how the models evolved from earlier publications and how new components were derived. Scientific journals should encourage authors to submit their astrocyte models, data, and cell morphologies into public databases. Eight, utility and applicability of the models, in comparison to other similar models developed, should be assessed. Ninth, standardized data-analysis, sensitivity-analysis, and simulation methods and tools are clearly needed for neuron-astrocyte network modeling. Neuroinformatics tools, such as tools for model description, simulation, sensitivity analysis, simulated data analysis, and model fitting to data, are mainly focused on neuronal description and modeling and need to be extended and validated with astrocytic data as well.

Advancing the model development workflows and extending the neuroinformatics tools are important steps towards better reproducibility, standardization, and easier sharing of astrocyte models. These are necessary for accelerating model development, for incorporating more biological complexity into data-driven models, and for integrating astrocytic mechanisms into the large-scale realistic models of brain systems. The guidelines developed in our study will be significant for facilitating our understanding of the brain and mental activities such as learning, memory, perception, and attention (Grillner et al., 2016; Amunts et al., 2022). We believe that our present study supports further development of standardized tools focused on astrocyte models, by conceptualizing the existing modeled cells, interaction mechanisms, and interaction schemes, by studying the differences and similarities of approaches and models, and by critically contrasting the properties of biophysical models with the properties of phenomenological models.

## Conclusion

During the past three decades, we have witnessed an increasing interest in glioscience research that resulted in exciting new knowledge about complex molecular- and cellular-level machinery in astrocytes and their multiple contributions to the functions of brain circuits, first in cell culture conditions and, later, mostly in brain slices (Bazargani & Attwell, 2016). As the knowledge of the existence, importance, and roles of astrocytes in the in vivo brain studies has expanded, new computational models of astrocytic functions have been increasingly published. Also, the interest in simulating astrocytic functions in larger neural systems, such as generic neuronal networks and brain circuits, is increasing. We critically evaluated 32 selected models of neuron-astrocyte networks, characterized model components by deriving evolutionary trees, classified spatial organization of cells and structure of interaction schemes used in the models, discussed impact of these models, identified elements in these models that would particularly benefit from new advanced data and tools, and prepared a list of guidelines for development of future large-scale and more biologically realistic models. Shortly, **(1)** the modeling community should carefully look at the newly accumulating experimental data when planning the future multi-level large-scale modeling projects and clearly explaining the justification of the biological (morphological, physiological, cell and molecular biological) choices made, **(2)** accurate and understandable modeling workflows should be used during the actual modeling, simulation, and publication process (see recommendations in Eriksson et al., 2022), **(3)** each new model should be tested and validated based on experimental data and contrasted, at least qualitatively, with other published models for consistent and reproducible behavior, and **(4)** the models should be properly documented and implemented in community supported open-access simulation tools. It is highly important that these aspects are assessed during the review process of scientific publications. As the evidence of astrocytic roles in diverse brain functions and dysfunctions accumulates, it is of increasing importance to develop reproducible, data-driven computational models at a sufficient level of biological detail and accelerate research towards understanding astrocytic contributions in health and disease.

**Table 1:** Characteristics of neuron-astrocyte network models. This table lists several details for each study: the tool or programming language used, code availability online, brain area, experimental data used, number of neurons and astrocytes, and experimentally shown neural function or other function that the model was finetuned to capture

**Study**	**Tool/ availability**	**Brain area/data**	**Neurons**	**Astrocytes**	**Function**
Abed et al. (2020)	n/a	Generic	10,000 E & I	n/a	Sgn./Inf.
Aleksin et al. (2017)	Arachne/GitHub	Hippocampal CA1	100 PY & 100 IN	100	Sync., Plast.
Allegrini et al. (2009)	n/a	Cortex	39 E & 10 I	400	Sync.
Amiri et al. (2012a)	n/a	Hippocampal CA1	5 PY & 5 IN	5	Sync.
Amiri et al. (2012b)	Simulink^®^	Thalamocortical	Lumps of PY, IN, TC & RE	Lumps	Hyper.
Amiri et al. (2012c)	n/a	Thalamocortical	Lumps of PY & IN	Lumps	Sync.
Amiri et al. (2013a)	Simulink^®^	Hippocampal CA1/in vitro LFP	50 PY & 50 IN	50	Sync.
Chan et al. (2017)	C++	Cortex/MEA cultures	8,000 E & 2,000 I	10,000	Sync.
Gordleeva et al. (2019)	n/a	Hippocampal CA1-CA3	2, 4, 36, 100 E	1–2	Ca^2+^, Sync., Plast.
Haghiri et al. (2016)	HW	Generic	2–100 E	1–90	Sync., HW
Haghiri et al. (2017)	HW	Generic	2–1,000 E	1–500	Sync., HW
Haghiri and Ahmadi (2020)	n/a	Generic	1,000 E	500	Sync.
Hayati et al. (2016)	HW	Generic	n/a E	n/a	Sync., Plast., HW
Kanakov et al. (2019)	n/a	Hippocampus	5 E & 1 I / 6 E	6	Sgn./Inf.
Lenk et al. (2020)	INEXA	Generic	200 E & 50 I	28, 63, 107	Sync.
Li et al. (2016)	n/a	Hippocampus	50 PY & 50 IN	50	Sgn./Inf.
Li et al. (2020)	Brian 2	Cortex	400 E & 100 I	400	Sync., E-I balance
Liu and Li (2013a)	n/a	Cortex	800 E & 200 I / 1,000 E	4,221	Sgn./Inf., Sync.
Liu and Li (2013b)	n/a	Generic	3 E & I	6	Ca^2+^, Sgn./Inf.
Liu et al. (2016)	HW	Generic	10–250,000 E	1–25,000	HW
Makovkin et al. (2020)	n/a	Generic	2 E / 2 I	2	Sync.
Mesiti et al. (2015)	n/a	Hippocampal CA3	2 PY	1, 20	Ca^2+^, Plast.
Naeem et al. (2015)	n/a	Generic	22–110 E	1–5	Plast.
Nazari and Faez (2019)	n/a	Cortex	2,500 PY & 2,500 IN	2,500	Sgn./Inf., Classif.
Nazari et al. (2020)	n/a	Cortex	4,010 PY & 1,000 IN	1,501,674	Sgn./Inf., Classif.
Postnov et al. (2009)	n/a	Generic	2–3 E	1–10	Ca^2+^, E-I balance
Soleimani et al. (2015)	HW	Generic	n/a E	1–24	Sync., HW
Stimberg et al. (2019)	Brian 2/GitHub	Neocortex	3,200 E & 800 I	3,200	Sync.
Tang et al. (2017)	n/a	Cortex	100 E	100	Hyper.
Yang and Yeo (2015)	n/a	Spinal cord	107 E	28	Sgn./Inf.
Yao et al. (2018)	n/a	Cortex	1–6 E	1–6	Hyper.
Yu et al. (2020)	n/a	Hippocampal CA3	50 PY & 50 IN	50	Sync.

**Table 2:** Characteristics of cellular interactions in the neuron-astrocyte network models. This table lists for each study, the number of connections and mechanisms in neuron-to-neuron (NN), neuron-to-astrocyte (NA), astrocyte-to-neuron (AN), and astrocyte-to-astrocyte (AA) interactions. Only those model details are shown that were clearly given in the model publications. Neurons are marked with N, excitatory neurons with E or PY, inhibitory neurons with I or IN, postsynaptic neurons with post, presynaptic neurons with pre, astrocytes with A or ast, astrocytic with astro, synaptic with syn, and extrasynaptic and extracellular with ext. Each type of interaction is characterized separately: interactions between excitatory neurons (EE), from excitatory to inhibitory neurons (EI), from inhibitory to excitatory neurons (IE), between inhibitory neurons (II), from excitatory neurons to astrocytes (EA), from astrocytes to excitatory neurons (AE), from inhibitory neurons to astrocytes (IA), from astrocytes to inhibitory neurons (AI), and between astrocytes (AA). If there is no number after a connection, for example after AA, then we were not sure how many connections between astrocytes there were. We used notation N in the mechanisms if the model publication did not specify if the mechanism was related to the presynaptic or postsynaptic neuron or both. Amounts modeled in concentrations are given inside square brackets. Note that synaptic conductance can be written as $g_{\textrm{syn}} = \bar{g}_{\textrm{syn}} s_{\textrm{syn}}$, where synaptic gating variable $s_{\textrm{syn}}$ can be, for example, $s_{\textrm{EE}}$. $I_{\textrm{astro}}=2.11\mathcal {H}(\ln (\Delta \textrm{Ca}))\ln (\Delta \textrm{Ca})$, where $\mathcal {H}$ is the heaviside function and $\Delta \textrm{Ca} = \mathrm {[Ca^{2+}]_{ast}} - 196.69 (\textrm{nM})$ (Nadkarni & Jung, 2003). In the models by Mesiti et al. (2015) and Li et al. (2020), $I_{\textrm{ast,AMPAR}} = \bar{g}_{\textrm{AMPAR}} s_{\textrm{AMPAR}} (V_{\textrm{m,post}} - V_{\textrm{AMPARpost}})$ and $I_{\textrm{ast,NMDAR}} = \bar{g}_{\textrm{NMDAR}} B_{\textrm{Mg}} s_{\textrm{NMDAR}} (V_{\textrm{m,post}} - V_{\textrm{NMDARpost}})$

**Study**	**NN interactions**	**NA interactions**	**AN interactions**	**AA interactions**
Abed et al. (2020)	EE/EI/IE/II: $V_{\textrm{m,pre}} \rightarrow S \rightarrow V_{\textrm{m,post}}$	EA/IA: $V_{\textrm{m,pre}} \rightarrow \textrm{NT} \rightarrow \mathrm {IP_{3ast}}$	AE/AI: $I_{\textrm{ast}} \rightarrow V_{\textrm{m,post}}$, $I_{\textrm{ast}}=c\mathrm {Glu_{ext}}$	AA: IP_3_ via GJs
Aleksin et al. (2017)	EE/II(2), EI/IE(0–1): $V_{\textrm{m,pre}} \rightarrow g_{\textrm{syn}} \rightarrow I_{\textrm{syn}} \rightarrow V_{\textrm{m,post}}$	EA/IA(0–1): $V_{\textrm{m,pre}} \rightarrow \mathrm {[NT]} \rightarrow \mathrm {[IP_3]_{ast}}$	AE/AI(0–1): $\mathrm {[Ca^{2+}]_{ast}} \rightarrow p_{\textrm{syn,rel}}$	AA(2): Ca^2+^ via GJs
Allegrini et al. (2009)	EE/EI/IE/II: $V_{\textrm{m,pre}} \rightarrow S \rightarrow V_{\textrm{m,post}}$	EA(1): $V_{\textrm{m,pre}} \rightarrow \mathrm {[IP_3]_{ast}}$	AE/AI: $I_{\textrm{astro}} \rightarrow V_{\textrm{m,post}}$	AA(2–4): Ca^2+^ and IP_3_ via GJs
Amiri et al. (2012a)	EE/II(0–1), EI(1), IE(1–2): $V_{\textrm{m,pre}} \rightarrow \textrm{NT} \rightarrow g_{\textrm{syn}} \rightarrow I_{\textrm{syn}} \rightarrow V_{\textrm{m,post}}$	EA(1): $V_{\textrm{m,pre}} \rightarrow \textrm{NT} \rightarrow \mathrm {[IP_3]_{ast}}$	AE/AI(1): $I_{\textrm{ast}} \rightarrow V_{\mathrm {m,PY/IN}}$, $I_{\textrm{ast}}=cf$	AA(1–2): IP_3_ via GJs
Amiri et al. (2012b)	EE/EI/IE: $V_{\textrm{m,pre}} \rightarrow g_{\mathrm{syn},\mathrm{AMPAR}/\mathrm{GABAAR}/\mathrm{GABABR}} \rightarrow$ $I_{\mathrm{syn},\mathrm{AMPAR}/\mathrm{GABAAR}/\mathrm{GABABR}} \rightarrow V_{\textrm{m,post}}$, $C_{\textrm{gain}}$	EA: $F_{\textrm{PY}} \rightarrow S_{\textrm{m}}$	AE/AI: $G_{\textrm{m}} \rightarrow C_{\textrm{gain}}$	AA: IP_3_ via GJs
Amiri et al. (2012c)	EE/EI/IE: $V_{\textrm{m,pre}} \rightarrow g_{\mathrm{syn},\mathrm{AMPAR}/\mathrm{GABAAR}/\mathrm{GABABR}} \rightarrow$ $I_{\mathrm{syn},\mathrm{AMPAR}/\mathrm{GABAAR}/\mathrm{GABABR}} \rightarrow V_{\textrm{m,post}}$, $C_{\textrm{gain}}$	EA: $F_{\textrm{PY}} \rightarrow S_{\textrm{m}}$	AE/AI: $G_{\textrm{m}} \rightarrow C_{\textrm{gain}}$	AA: IP_3_ via GJs
Amiri et al. (2013a)	EE/II(0–1), EI(1), IE(1–2): $V_{\textrm{m,pre}} \rightarrow \textrm{NT} \rightarrow g_{\textrm{syn}} \rightarrow I_{\textrm{syn}} \rightarrow V_{\textrm{m,post}}$	EA/IA(1): $V_{\textrm{m,pre}} \rightarrow \textrm{NT} \rightarrow \mathrm {[IP_3]_{ast}}$	AE/AI(1): $I_{\textrm{ast}} \rightarrow V_{\mathrm {m,PY/IN}}$, $I_{\textrm{ast}}=cf$	AA(1–2): IP_3_ via GJs
Chan et al. (2017)	Network 1 & 2: EE($p=0.19$), EI($p=0.23$), IE($p=0.21$), II($p=0.17$): $V_{\textrm{m,pre}} \rightarrow g_{\textrm{syn}} \rightarrow I_{\textrm{syn}} \rightarrow V_{\textrm{m,post}}$	Network 1 & 2: EA/IA: $V_{\textrm{m,N}} \rightarrow \mathrm {[IP_3]_{ast}}$	Network 1 & 2: AE/AI: $I_{\textrm{ast}} \rightarrow V_{\mathrm {m,E/I}}$, $I_{\textrm{ast}}=cf$	Network 2: AA: IP_3_ via GJs
Gordleeva et al. (2019)	EE($p=0.2$): $V_{\textrm{m,pre}} \rightarrow \mathrm {Glu_{syn}} \rightarrow$ $I_{\textrm{syn,NMDAR}} \rightarrow V_{\textrm{m,post}}$	EA(1–2): $V_{\textrm{m,pre}} \rightarrow \mathrm {Glu_{syn}} \rightarrow \mathrm {[IP_3]_{ast}}$	AE(1, 14, 28): $\mathrm {[Ca^{2+}]_{ast}} \rightarrow \mathrm {D\text {-}serine_{ext}}\rightarrow I_{\textrm{syn,NMDAR}} \rightarrow V_{\textrm{m,post}}$, $\mathrm {[Ca^{2+}]_{ast}} \rightarrow \mathrm {Glu_{ext}} \rightarrow\mathrm {Glu_{syn}} \rightarrow I_{\mathrm{syn,NMDAR}}\rightarrow V_{\textrm{m,post}}$	AA(1): Ca^2+^ and IP_3_ via GJs
Haghiri et al. (2016)	Network 1 & 2: EE(0–1): $V_{\textrm{m,pre}} \rightarrow z \rightarrow I_{\textrm{syn}} \rightarrow V_{\textrm{m,post}}$	Network 1: EA(1–4); Network 2: EA(1–2): $u_{\textrm{post}} \rightarrow \mathrm {{Ca^{2+}_{ast}}}$, $V_{\textrm{m,pre}} \rightarrow z \rightarrow S_{\textrm{m}}$	Network 1 & 2: AE(1): $G_{\textrm{m}} \rightarrow I_{\textrm{syn}} \rightarrow V_{\textrm{m,post}}$, $I_{\textrm{ast}} \rightarrow V_{\textrm{m,post}}$, $I_{\textrm{ast}}=cG_{\textrm{m}}$, $I_{\textrm{syn}} = (k-cG_{\textrm{m}})(z-z_0)$	Network 2: AA(1–2): n/a
Haghiri et al. (2017)	Network 1: EE(0–1); Network 2: EE: $V_{\textrm{m,pre}} \rightarrow z \rightarrow I_{\textrm{syn}} \rightarrow V_{\textrm{m,post}}$	Network 1: EA(1–2); Network 2: EA(0–2): $u_{\textrm{post}} \rightarrow \mathrm {{Ca^{2+}_{ast}}}$, $V_{\textrm{m,pre}} \rightarrow z \rightarrow S_{\textrm{m}}$	Network 1 & 2: AE(1): $G_{\textrm{m}} \rightarrow I_{\textrm{syn}} \rightarrow V_{\textrm{m,post}}$, $I_{\mathrm {ast,ATP/Glu}} \rightarrow V_{\textrm{m,post}}$, $I_{\textrm{ast,ATP}}=cG_{\textrm{a}}$, $I_{\textrm{ast,Glu}}=cG_{\textrm{m}}$, $I_{\textrm{syn}} = (k-cG_{\textrm{m}})(z-z_0)$	None
Haghiri and Ahmadi (2020)	EE: $V_{\textrm{m,pre}} \rightarrow z \rightarrow I_{\textrm{syn}} \rightarrow V_{\textrm{m,post}}$	EA(1): $u_{\textrm{post}} \rightarrow \mathrm {{Ca^{2+}_{ast}}}$, $V_{\textrm{m,pre}} \rightarrow z \rightarrow S_{\textrm{m}}$	AE(1): $G_{\textrm{m}} \rightarrow I_{\textrm{syn}} \rightarrow V_{\textrm{m,post}}$, $I_{\textrm{ast}} \rightarrow V_{\textrm{m,post}}$, $I_{\textrm{ast}}=cG_{\textrm{m}}$, $I_{\textrm{syn}} = (k-cG_{\textrm{m}})(z-z_0)$	None
Hayati et al. (2016)	EE(0–1): $V_{\textrm{m,pre}} \rightarrow z \rightarrow I_{\textrm{syn}} \rightarrow V_{\textrm{m,post}}$	EA(1–4): $V_{\textrm{m,pre}} \rightarrow z \rightarrow S_{\textrm{m}}$, $w_{\textrm{post}} \rightarrow \mathrm {{Ca^{2+}_{ast}}}$	AE(2): $G_{\textrm{m}} \rightarrow I_{\textrm{syn}} \rightarrow V_{\textrm{m,post}}$, $I_{\textrm{ast}} \rightarrow V_{\textrm{m,post}}$, $I_{\textrm{ast}}=cG_{\textrm{m}}$, $I_{\textrm{syn}} = (k-cG_{\textrm{m}})(z-z_0)$	AA: n/a
Kanakov et al. (2019)	Network 1: EE/EI/IE($p=0.33$); Network 2: EE(5): $V_{\textrm{m,pre}} \rightarrow g_{\textrm{syn}} \rightarrow I_{\textrm{syn}} \rightarrow V_{\textrm{m,post}}$	Network 1: EA/IA(1); Network 2: EA(1): n/a	Network 1: AE/AI(1); Network 2: AE(1): $\mathrm {[Ca^{2+}]_{ast}} \rightarrow g_{\textrm{syn}}$ $\rightarrow I_{\textrm{syn}} \rightarrow V_{\textrm{m,post}}$	Network 1 & 2: AA(2–3): Ca^2+^ and IP_3_ via GJs
Lenk et al. (2020)	EE/EI/IE/II($p=0.29$): $F_{\textrm{pre}} \rightarrow p_{\textrm{spike}} \rightarrow p_{\textrm{syn,rel}}$ $\rightarrow \textrm{NT} \rightarrow I_{\textrm{syn}} \rightarrow F_{\textrm{post}}$	EA(0–1): $F_{\textrm{pre}} \rightarrow p_{\textrm{spike}} \rightarrow$ $p_{\textrm{syn,rel}} \rightarrow \textrm{NT} \rightarrow \mathrm {[IP_3]_{ast}}$	AE/AI(130–250): $\mathrm {[Ca^{2+}]_{ast}} \rightarrow s_{\textrm{Rpre}} \rightarrow p_{\textrm{syn,rel}}$, $S_{\textrm{ast}} \rightarrow F_{\textrm{post}}$	AA(1–5): IP_3_ via GJs
Li et al. (2016)	EE/II(0–1), EI(1), IE(1–2): $V_{\textrm{m,pre}} \rightarrow \textrm{NT} \rightarrow g_{\textrm{syn}}$ $\rightarrow I_{\textrm{syn}} \rightarrow V_{\textrm{m,post}}$	EA/IA(1): $V_{\textrm{m,pre}} \rightarrow \textrm{NT} \rightarrow \mathrm {[IP_3]_{ast}}$	AE/AI(1): D_ext_: [ATP]_ext_ and [Glu]_ext_, $I_{\textrm{ast,ATP}} \rightarrow V_{\textrm{m,PY}}$, $I_{\textrm{ast,Glu}} \rightarrow V_{\textrm{m,IN}}$, $I_{\textrm{ast,ATP}}=c\mathrm {[ATP]_{ext}}$, $I_{\textrm{ast,Glu}}=c\mathrm {[Glu]_{ext}}$	AA(1–2): IP_3_ via GJs
Li et al. (2020)	EE/EI/IE/II($p=0.2$): $V_{\textrm{m,pre}} \rightarrow p_{\textrm{syn,rel}} \rightarrow \mathrm {[Glu]_{syn}} \rightarrow g_{\mathrm {syn,AMPAR/NMDAR}} \rightarrow$ $I_{\mathrm {syn,AMPAR/NMDAR}} \rightarrow V_{\textrm{m,post}}$, $V_{\textrm{m,pre}} \rightarrow p_{\textrm{syn,rel}} \rightarrow \mathrm {[GABA]_{syn}} \rightarrow g_{\textrm{syn,GABAAR}} \rightarrow$ $I_{\textrm{syn,GABAAR}} \rightarrow V_{\textrm{m,post}}$	EA($\approx$ 100): $V_{\textrm{m,pre}} \rightarrow p_{\textrm{syn,rel}} \rightarrow$ $\mathrm {[Glu]_{syn}} \rightarrow \mathrm {[IP_3]_{ast}}$	AE($\approx$ 100): $\mathrm {[Ca^{2+}]_{ast}} \rightarrow p_{\textrm{ast,rel}} \rightarrow \mathrm {[Glu]_{ext}} \rightarrow$ $s_{\textrm{mGluRpre}} \rightarrow p_{\textrm{syn,rel}}$, $\mathrm {[Ca^{2+}]_{ast}} \rightarrow p_{\textrm{ast,rel}} \rightarrow$ $\mathrm {[Glu]_{ext}} \rightarrow g_{\mathrm {syn,AMPAR/NMDAR}} \rightarrow$ $I_{\mathrm {ast,AMPAR/NMDAR}} \rightarrow V_{\textrm{m,post}}$	AA($\approx$ 4): IP_3_ via GJs
Liu and Li (2013a)	Network 1: EE/IE(80), EI/II(20); Network 2: EE(100): $V_{\textrm{m,pre}} \rightarrow g_{\textrm{syn}} \rightarrow I_{\textrm{syn}} \rightarrow V_{\textrm{m,post}}$	Network 1 & 2: EA: $V_{\textrm{m,pre}} \rightarrow \mathrm {[Glu]_{syn}} \rightarrow \mathrm {[IP_3]_{ast}}$	Network 1: AE/AI; Network 2: AE: $I_{\textrm{astro}} \rightarrow V_{\textrm{m,N}}$	Network 1 & 2: AA(2–4): Ca^2+^ and IP_3_ via GJs
Liu and Li (2013b)	Network 1: EE(0–2); Network 2: EE/EI(0–1), IE(1); Network 3: EE(0–1), EI(1); Network 4: EI(2), II(0–1): $V_{\textrm{m,pre}} \rightarrow g_{\textrm{syn}} \rightarrow I_{\textrm{syn}} \rightarrow V_{\textrm{m,post}}$	Network 1, 2, 3 & 4: EA(1): $V_{\textrm{m,pre}} \rightarrow \mathrm {[IP_3]_{ast}}$	Network 1: AE(0–1); Network 2 & 3: AE/AI(0–1); Network 4: AI(0–1): $I_{\textrm{astro}} \rightarrow V_{\textrm{m,N}}$	Network 1, 2, 3 & 4: AA(2–3): Ca^2+^ and IP_3_ via GJs
Liu et al. (2016)	EE: $p_{\textrm{syn,rel}} \rightarrow I_{\textrm{syn}} \rightarrow V_{\textrm{m,post}}$, $V_{\textrm{m,post}} \rightarrow \mathrm {[2\text {-}AG]_{post}}$ $\rightarrow \textrm{DSE} \rightarrow p_{\textrm{syn,rel}}$	EA: $V_{\textrm{m,post}} \rightarrow \mathrm {[2\text {-}AG]_{post}} \rightarrow \mathrm {[IP_3]_{ast}}$	AE: $\mathrm {[Ca^{2+}]_{ast}} \rightarrow \mathrm {[Glu]_{ext}} \rightarrow \mathrm {e\text {-}SP} \rightarrow p_{\textrm{syn,rel}}$	AA: IP_3_ via GJs
Makovkin et al. (2020)	Network 1: EE(0–1); Network 2: II(0–1): $V_{\textrm{m,pre}} \rightarrow g_{\textrm{syn}} \rightarrow I_{\textrm{syn}} \rightarrow V_{\textrm{m,post}}$	Network 1: EA(1); Network 2: IA(1): $V_{\mathrm {m,pre/post}} \rightarrow \textrm{NT} \rightarrow \mathrm {[IP_3]_{ast}}$	Network 1: AE(0–1); Network 2: AI(0–1): $\mathrm {[Ca^{2+}]_{ast}} \rightarrow g_{\textrm{syn}} \rightarrow I_{\textrm{syn}} \rightarrow V_{\textrm{m,post}}$	Network 1 & 2: AA(1): Ca^2+^ and IP_3_ via GJs
Mesiti et al. (2015)	EE(0–1): $V_{\textrm{m,presoma}} \rightarrow g_{\mathrm {syn,AMPAR/NMDAR}} \rightarrow$ $I_{\mathrm {syn,AMPAR/NMDAR}} \rightarrow V_{\textrm{m,postdent}}$	EA(0–1): $V_{\textrm{m,presoma}} \rightarrow \mathrm {[IP_3]_{ast}}$	AE(0–2): $\mathrm {[Ca^{2+}]_{ast}} \rightarrow \mathrm {[Ca^{2+}]_{pre}}$, $\mathrm {[Ca^{2+}]_{ast}} \rightarrow g_{\mathrm {syn,AMPAR/NMDAR}} \rightarrow$ $I_{\mathrm {ast,AMPAR/NMDAR}} \rightarrow V_{\textrm{m,postdent}}$, $I_{\textrm{astro}} \rightarrow V_{\textrm{m,presoma}}$	AA(1–2): IP_3_ via GJs
Naeem et al. (2015)	EE(1): $p_{\textrm{syn,rel}} \rightarrow I_{\textrm{syn}} \rightarrow V_{\textrm{m,post}}$, $V_{\textrm{m,post}} \rightarrow \mathrm {[2\text {-}AG]_{post}} \rightarrow \textrm{DSE} \rightarrow p_{\textrm{syn,rel}}$	EA(0–1): $V_{\textrm{m,post}} \rightarrow \mathrm {[2\text {-}AG]_{post}} \rightarrow \mathrm {[IP_3]_{ast}}$	AE(1): $\mathrm {[Ca^{2+}]_{ast}} \rightarrow \mathrm {[Glu]_{ext}} \rightarrow \mathrm {e\text {-}SP} \rightarrow p_{\textrm{syn,rel}}$	AA(2): IP_3_ via GJs
Nazari and Faez (2019)	EE/EI/IE/II($p=0.08$): $V_{\textrm{m,pre}} \rightarrow x_{\mathrm {AMPAR/GABAR}} \rightarrow$ $I_{\mathrm {syn,AMPAR/GABAR}} \rightarrow V_{\textrm{m,post}}$	EA/IA(1): $V_{\mathrm {m,pre/post}} \rightarrow \textrm{NT} \rightarrow \mathrm {[IP_3]_{ast}}$	AE/AI(1): $I_{\textrm{ast}} \rightarrow x_{\mathrm {AMPAR/GABAR}} \rightarrow$ $I_{\mathrm {syn,AMPAR/GABAR}}$ $\rightarrow V_{\mathrm {m,PY/IN}}$, $I_{\textrm{ast}}=c\mathrm {[Ca^{2+}]_{ast}}$	AA($p=0.1$): IP_3_ via GJs
Nazari et al. (2020)	L2: EE/EI/IE/II($p=0.2$); From L2 to output layer: EE/IE(10): $V_{\textrm{m,pre}} \rightarrow x_{\mathrm {AMPAR/GABAR}} \rightarrow$ $I_{\mathrm {syn,AMPAR/GABAR}} \rightarrow V_{\textrm{m,post}}$	L2: EA/IA(0–1): $V_{\mathrm {m,pre/post}} \rightarrow \textrm{NT} \rightarrow \mathrm {[IP_3]_{ast}}$	L2: AE/AI(1): $I_{\textrm{ast}} \rightarrow x_{\mathrm {AMPAR/GABAR}} \rightarrow$ $I_{\mathrm {syn,AMPAR/GABAR}} \rightarrow$ $V_{\mathrm {m,PY/IN}}$, $I_{\textrm{ast}}=c\mathrm {[Ca^{2+}]_{ast}}$	L2: AA(1,501,673): IP_3_ via GJs
Postnov et al. (2009)	EE(1): $V_{\textrm{m,pre}} \rightarrow z \rightarrow I_{\textrm{syn}} \rightarrow w_{\textrm{post}}$	EA(1): $V_{\textrm{m,pre}} \rightarrow z \rightarrow S_{\textrm{m}}$, $w_{\textrm{post}} \rightarrow \mathrm {{Ca^{2+}_{ast}}}$	AE: $G_{\textrm{m}} \rightarrow I_{\textrm{syn}} \rightarrow w_{\textrm{post}}$, $I_{\mathrm {ast,ATP/Glu}} \rightarrow w_{\textrm{post}}$, $I_{\textrm{ast,ATP}}=cG_{\textrm{a}}$, $I_{\textrm{ast,Glu}}=cG_{\textrm{m}}$, $I_{\textrm{syn}} = (k-cG_{\textrm{m}})(z-z_0)$	AA: Ca^2+^ and IP_3_ via GJs, D_ext_: ATP_ext_ ($G_{\textrm{a}}$) and Glu_ext_ ($G_{\textrm{m}}$)
Soleimani et al. (2015)	Network 1: EE(2–4); Network 2: EE: $X_{\textrm{pre}} \rightarrow I_{\textrm{syn}} \rightarrow X_{\textrm{post}}$, $Y_{\textrm{pre}} \rightarrow I_{\textrm{syn}} \rightarrow Y_{\textrm{post}}$	Network 1: EA(2–4); Network 2: EA: $X_{\textrm{N}} \rightarrow Z \rightarrow S_{\textrm{m}}$, $Y_{\textrm{N}} \rightarrow Z \rightarrow S_{\textrm{m}}$	Network 1 & 2: AE(1): $I_{\textrm{ast}} \rightarrow X_{\textrm{N}}$, $I_{\textrm{ast}} \rightarrow Y_{\textrm{N}}$, $I_{\textrm{ast}} = c \mathrm {Ca^{2+}}$	None
Stimberg et al. (2019)	EE/IE($p=0.05$), EI/II($p=0.2$):$V_{\textrm{m,pre}} \rightarrow p_{\textrm{syn,rel}} \rightarrow$ $g_{\textrm{syn}} \rightarrow I_{\textrm{syn}} \rightarrow V_{\textrm{m,post}}$	EA(1): $V_{\textrm{m,pre}} \rightarrow p_{\textrm{syn,rel}} \rightarrow \mathrm {[NT]} \rightarrow$ $s_{\textrm{mGluRast}} \rightarrow \mathrm {[IP_3]_{ast}}$	AE(1): $\mathrm {[Ca^{2+}]_{ast}} \rightarrow p_{\textrm{ast,rel}} \rightarrow$ $\mathrm {[GT]} \rightarrow s_{\textrm{Rpre}} \rightarrow p_{\textrm{syn,rel}}$	AA: IP_3_ via GJs
Tang et al. (2017)	EE(2–4): $V_{\textrm{m,pre}} \rightarrow S \rightarrow V_{\textrm{m,post}}$	EA(1): $V_{\textrm{m,N}} \rightarrow \mathrm {[IP_3]_{ast}}$	AE(1): $I_{\textrm{astro}} \rightarrow V_{\textrm{m,N}}$	AA(1–2): IP_3_ via GJs
Yang and Yeo (2015)	EE: $\mathrm {[Glu]_{syn}}$	EA: $\mathrm {[Glu]_{syn}} \rightarrow \mathrm {[IP_3]_{ast}}$	AE: $\mathrm {[ATP]_{ext}}\rightarrow \mathrm {NMDAR_{post}}$, $\mathrm {[Glu]_{ext}} \rightarrow \mathrm {NMDAR_{post}}$	AA: IP_3_ via GJs, D_ext_: [ATP]_ext_ and [Glu]_ext_
Yao et al. (2018)	EE(0–1): [K^+^]_ext_, [Na^+^]_ext_,$V_{\textrm{m,pre}} \rightarrow \mathrm {[Glu]_{ext}} \rightarrow$ $I_{\mathrm {syn,K/NaNMDAR}} \rightarrow V_{\textrm{m,post}}$	EA(1): [K^+^]_ext_, [Na^+^]_ext_	AE(1): [K^+^]_ext_, [Na^+^]_ext_, $\mathrm {[ATP]_{ext}} \rightarrow \mathrm {[Glu]_{ext}} \rightarrow$ $I_{\mathrm {syn,K/NaNMDAR}} \rightarrow V_{\textrm{m,post}}$	AA: [K^+^]_ext_, [Na^+^]_ext_, $\mathrm {[ATP]_{ext}} \rightarrow \mathrm {G_{ast}} \rightarrow \mathrm {[IP_3]_{ast}}$, $\mathrm {[ATP]_{ext}} \rightarrow \mathrm {[Glu]_{ext}}$, D_ext_: [ATP]_ext_, [K^+^]_ext_, and [Na^+^]_ext_
Yu et al. (2020)	EE/II(0–1), EI(1), IE(1–2): $V_{\textrm{m,pre}} \rightarrow \textrm{NT} \rightarrow g_{\textrm{syn}} \rightarrow I_{\textrm{syn}} \rightarrow V_{\textrm{m,post}}$	EA/IA(50): $V_{\textrm{m,pre}} \rightarrow \textrm{NT} \rightarrow \mathrm {[IP_3]_{ast}}$	AE/AI(50): $I_{\textrm{ast}} \rightarrow V_{\mathrm {m,PY/IN}}$, $I_{\textrm{ast}}=c \sum f$	AA(1–2): IP_3_ via GJs

**Table 3:** Characteristics of spatial organization and structure of interaction scheme between modeled cells. For each study, **the second column** lists the categorization of the entire model (‘All cells’) and of each separate cell type (E/I/A) according to the spatial organization of cells. The categories are illustrated in Fig. 8A1–C2. **The third column** lists the structure of the interaction scheme for each model according to the categories in Fig. 8a–f. Each type of interaction is characterized separately: interactions between excitatory neurons (EE), from excitatory to inhibitory neurons (EI), from inhibitory to excitatory neurons (IE), between inhibitory neurons (II), from excitatory neurons to astrocytes (EA), from astrocytes to excitatory neurons (AE), from inhibitory neurons to astrocytes (IA), from astrocytes to inhibitory neurons (AI), and between astrocytes (AA). **The fourth column** shows direction of the information flow for each model. We characterized the global information flow (Fig. 8I), when considering the entire model, under ‘Global’. Local interaction flow (Fig. 8II) is characterized for each interaction type: between excitatory neurons (EE), between excitatory and inhibitory neurons (EI(IE)), between inhibitory neurons (II), between excitatory neurons and astrocytes (EA(AE)), between inhibitory neurons and astrocytes (IA(AI)), and between astrocytes (AA). Note that in this case, unlike in analysis of structure of the interaction scheme in the third column, we had to consider interactions in both directions together. Thus, we used different notation in the third column compared to the fourth column, for example EA(AE) means that we considered both, the interaction from E to A and from A to E. If both interactions existed between the same two cells, the model was categorized as (locally) recurrent, if one of them did not exist, the model was (locally) feed-forward

**Study**	**Spatial organization of cells**	**Structure of interaction scheme**	**Direction of information flow**
Abed et al. (2020)	All cells/E/I/A: 2D, random placement	EE/EI/IE/II: all-to-all; EA/AE/IA/AI: one-to-one; AA: n/a	Global: recurrent. Local: EE/EI(IE)/II/AA: recurrent; EA(AE)/IA(AI): feed-forward
Aleksin et al. (2017)	All cells: 2D, grid of rings; E/I/A: 1D, ring	EE/II/AA: explicitly defined; EI/IE/EA/AE/IA/AI: one-to-one	Global: recurrent. Local: EE/II/EA(AE)/IA(AI)/AA: recurrent; EI(IE): feed-forward
Allegrini et al. (2009)	All cells/E/I/A: 2D, grid	EE/EI/IE/II/EA: random; AE/AI/AA: distance dependent; IA: none	Global: recurrent. Local: EE/EI(IE)/II/AA: recurrent; EA(AE)/IA(AI): feed-forward
Amiri et al. (2012a)	All cells: 2D, grid; E/I/A: 1D, array	EE/EI/II/EA/AE/AI: one-to-one; IE/AA: explicitly defined; IA: none	Global: feed-forward. Local: EE/II/IA(AI): feed-forward; EI(IE)/EA(AE)/AA: recurrent
Amiri et al. (2012b)	All cells/E/I/A: 3D, multiple populations; E = PY, TC; I = IN, RE	EE/EI/IE/EA/AE/AI/AA: explicitly defined; II/IA: none	Global: recurrent. Local: EE/EI(IE)/EA(AE)/AA: recurrent; II: none; IA(AI): feed-forward
Amiri et al. (2012c)	All cells/E/I/A: 3D, multiple populations	EE/EI/IE/EA/AE/AI/AA: explicitly defined; II/IA: none	Global: recurrent. Local: EE/EI(IE)/EA(AE)/AA: recurrent; II: none; IA(AI): feed-forward
Amiri et al. (2013a)	All cells: 2D, grid; E/I/A: 1D, array	EE/EI/II/EA/AE/IA/AI: one-to-one; IE/AA: explicitly defined	Global: feed-forward. Local: EE/II: feed-forward; EI(IE)/EA(AE)/IA(AI)/AA: recurrent
Chan et al. (2017)	Network 1: All cells/E/I/A: 2D, random placement; Network 2: All cells/E/I/A: 2D, grid	Network 1: EE/EI/IE/II/EA/AE/IA/AI: random; AA:none; Network 2: EE/EI/IE/II/EA/AE/IA/AI/AA: distance dependent	Global: recurrent. Local: EE/EI(IE)/II/EA(AE)/IA(AI): recurrent; Network 1: AA: none; Network 2: AA: recurrent
Gordleeva et al. (2019)	All cells/E: 2D, random placement; A: 1D, two-node motif	EE/EA: random; AE: distance dependent; AA: one-to-one	Global: recurrent. Local: EE/EA(AE)/AA: recurrent
Haghiri et al. (2016)	Network 1: All cells/E/A: 1D, few node motif; E: 1D, motif with convergent inputs; E and A: 1D, three-node motif; Network 2: All cells/E/A: 2D, grid	EE: one-to-one; EA/AE: explicitly defined; Network 1: AA: none; Network 2: AA: explicitly defined	Global: feed-forward. Local: EE: feed-forward; EA(AE): recurrent; Network 1: AA: none; Network 2: AA: recurrent
Haghiri et al. (2017)	Network 1: All cells/E/A: 1D, array; Network 2: All cells/E/A: 2D, random placement	AA: none; Network 1: EE: one-to-one; EA/AE: explicitly defined; Network 2: EE/EA/AE: random	Global: Network 1: feed-forward; Network 2: recurrent. Local: EA(AE): recurrent; AA: none; Network 1: EE: feed-forward; Network 2: EE: recurrent
Haghiri and Ahmadi (2020)	All cells/E/A: 2D, multilayer	AA:none; Between layers: EE: random; Within layers: EE/EA: one-to-one; AE: explicitly defined	Global: feed-forward. Local: EE: feed-forward; EA(AE): recurrent; AA: none
Hayati et al. (2016)	All cells/E/A: 2D, grid	EE: one-to-one; EA/AE/AA: explicitly defined	Global: feed-forward. Local: EE: feed-forward; EA(AE)/AA: recurrent
Kanakov et al. (2019)	All cells/E/I/A: 2D, grid	AE: one-to-one; AA: explicitly defined; Network 1: EE/EI/IE/EA/IA: random; II: none; AI: one-to-one; Network 2: EE/EA: all-to-all	Global: recurrent. Local: EE/EI(IE)/AA: recurrent; II: none; EA(AE)/IA(AI): n/a; Network 1: both E and I; Network 2: only E
Lenk et al. (2020)	All cells/E/I/A: 2D, random placement	EE/EI/IE/II/EA/AE/AI/AA: distance dependent; IA: none	Global: recurrent. Local: EE/EI(IE)/II/EA(AE)/AA: recurrent; IA(AI): feed-forward
Li et al. (2016)	All cells: 2D, grid; E/I/A: 1D, array	EE/EI/II/EA/AE/IA/AI: one-to-one; IE/AA: explicitly defined	Global: feed-forward. Local: EE/II: feed-forward; EI(IE)/EA(AE)/IA(AI)/AA: recurrent
Li et al. (2020)	All cells/E/I/A: 2D, random placement	EE/EI/IE/II: random; EA/AE/AA: distance dependent; IA/AI: none	Global: recurrent. Local: EE/EI(IE)/II/EA(AE)/AA: recurrent; IA(AI): none
Liu and Li (2013a)	All cells/A: 2D, grid; Network 1: E/I: 2D, multilayer; Network 2: E: 2D, multilayer	AE/AI/AA: explicitly defined; IA: none; Between neuron-defined layers: EE/EI/IE/II/EA: all-to-all; Within layers: EE/EI/IE/II: none; Network 1: both E and I; Network 2: only E	Global: feed-forward. Local: EE/EI(IE)/II/IA(AI): feed-forward; EA(AE)/AA: recurrent; Network 1: both E and I; Network 2: only E
Liu and Li (2013b)	All cells/A: 2D, grid; E/I: 1D, three-node motif	EE/EI/IE/II/EA/AE/AI/AA: explicitly defined; IA: none	Global: feed-forward. Local: EE/EI(IE)/II/IA(AI): feed-forward; EA(AE)/AA: recurrent
Liu et al. (2016)	All cells/E/A: 3D, multilayer	EE/EA/AE/AA: hierarchical	Global: model dependent. Local: EE/AA: recurrent; EA(AE): feed-forward
Makovkin et al. (2020)	All cells: 1D, four-node motif; E/I/A: 1D, two-node motif	Network 1: EE/EA/AE/AA: one-to-one; Network 2: II/IA/AI/AA: one-to-one	Global: recurrent. Local: AA: recurrent; Network 1: EE: feed-forward; EA(AE): recurrent; Network 2: II: feed-forward; IA(AI): recurrent
Mesiti et al. (2015)	All cells/A: 1D, array; E: 1D, two-node motif	EE/EA: one-to-one; AE/AA: explicitly defined	Global: recurrent. Local: EE: feed-forward; EA(AE)/AA: recurrent
Naeem et al. (2015)	All cells/A: 1D, ring; E: 1D, motif with convergent inputs	EE/EA: one-to-one; AE/AA: explicitly defined	Global: recurrent. Local: EE/AA: recurrent; EA(AE): feed-forward
Nazari and Faez (2019)	All cells/E/I/A: 2D, grid	EE/EI/IE/II/EA/AE/IA/AI/AA: random	Global: recurrent. Local: EE/EI(IE)/II/EA(AE)/IA(AI)/AA: recurrent
Nazari et al. (2020)	All cells/E/I: 3D, multilayer; A: 2D, grid	L2: EE/EI/IE/II/EA/AE/IA/AI: random; AA: all-to-all; L2 to Output layer: EE/IE: all-to-all	Global: feed-forward. Local: L2: EE/EI(IE)/II/EA(AE)/IA(AI)/AA: recurrent; L2 to Output layer: EE/EI(IE): feed-forward
Postnov et al. (2009)	All cells/E/A: 2D, random placement	EE: explicitly defined; EA/AE/AA: distance dependent	Global: recurrent. Local: EE/EA(AE)/AA: recurrent
Soleimani et al. (2015)	All cells/E/A: 2D, grid	AA: none; Network 1: EE/EA/AE: explicitly defined; Network 2: EE/EA/AE: all-to-all	Global: recurrent. Local: EE/EA(AE): recurrent; AA: none
Stimberg et al. (2019)	All cells/E/I/A: 2D, grid	EE/EI/IE/II/EA: random; AE/AA: distance dependent; IA/AI: none	Global: recurrent. Local: EE/EI(IE)/II/EA(AE)/AA: recurrent; IA(AI): none
Tang et al. (2017)	All cells/E/A: 1D, array	EE/AA: explicitly defined; EA/AE: one-to-one	Global: recurrent. Local: EE/EA(AE)/AA: recurrent
Yang and Yeo (2015)	All cells/E/A: 3D, multilayer; L1: E: 2D, grid	L1: EE: explicitly defined; Between L1 and L2: EE: one-to-one; Between L2 and L3: EA/AE: one-to-one; Between L3 and L4: AA: hierarchical	Global: feed-forward. Local: L1: EE: recurrent; Between L1 and L2: EE: feed-forward; Between L2 and L3: EA(AE): feed-forward; Between L3 and L4: AA: recurrent
Yao et al. (2018)	All cells/E/A: 1D, array	EE/EA/AE: one-to-one; AA: explicitly defined	Global: feed-forward. Local: EE: feed-forward; EA(AE)/AA: recurrent
Yu et al. (2020)	All cells: 2D, grid; E/I/A: 1D, array	EE/EI/II: one-to-one; IE/AA: explicitly defined; EA/AE/IA/AI: all-to-all	Global: feed-forward. Local: EE/EI(IE)/II/EA(AE)/IA(AI)/AA: recurrent

## Information Sharing Statement

All data analysed during the current study is available in earlier published studies and also in tables prepared by us and included in this published article and its supplementary information file.

## Supplementary Information

Below is the link to the electronic supplementary material.

Supplementary file1 (PDF 250 kb)

## Nomenclature

2-AG: 2-arachidonoylglycerol
3K: 3-kinase
5P: 5-phosphatase
$\Delta \textrm{Ca}$: Change in Ca^2+^ concentration (Nadkarni & Jung, 2003)
$\gamma$: Entry of matrix $\Gamma$
$$\Gamma$$: Binary matrix
$\chi$: Depletion of ATP stores
A: Astrocyte
AHP: Afterhyperpolarization
AMPAR: $\alpha$-amino-3-hydroxy-5-methyl-4-isoxazolepropionic acid receptor
ast: Astrocyte
astro: Astrocytic
ATP: Adenosine triphosphate
$B_{\textrm{Mg}}$: $V_{\textrm{m}}$-dependent equation related to Mg^2+^ block
*c*: Constant
$C_{\textrm{gain}}$: Gain between subpopulations
$c_{\textrm{KCa}}$: Gating variable for K_Ca_ activation
CA: Cornu ammonis
Ca^2+^: Calcium ion
Ca_T_: T-type low-threshold Ca^2+^ channel
CCE: Capacitive Ca^2+^ entry
CICR: Ca^2+^-induced Ca^2+^ release
Cl^−^: Chloride ion
Classif: Classification
cyt: Cytosol
D: Dimensional
D_cyt_: Diffusion in cyt
D_ER_: Diffusion in ER
D_ext_: Diffusion in ext
dend: Dendritic
Diff.: Diffusion
DSE: Depolarization-induced suppression of excitation
E: Excitatory
ER: Endoplasmic reticulum
e-SP: Endocannabinoid-mediated synaptic potentiation
ext: Extracellular space (can be also periastrocytic, perisynaptic, extrasynaptic, or perivascular space)
*f*: Phenomenological variable modeling events from Ca^2+^ rise to vesicle release
*F*: Firing rate
FF: Feed-forward
G: G protein
$$G_{\textrm{a}}$$: Astrocytic mediator
$\bar{g}_{\textrm{AMPAR}}$: Maximal conductance of AMPAR
$$G_{\textrm{m}}$$: Astrocytic mediator
$\bar{g}_{\textrm{NMDAR}}$: Maximal conductance of NMDAR
$$g_{\textrm{syn}}$$: Synaptic conductance
$\bar{g}_{\textrm{syn}}$: Maximal synaptic conductance
$g_{\textrm{tonic}}$: Gating variable of tonic current
GABA: Gamma-aminobutyric acid
GABA_A_R: A-type GABA receptor
GABA_B_R: B-type GABA receptor
GABAR: GABA reeptor
GJ: Gap junction
Glu: Glutamate
GT: Gliotransmitter
*h*: Active fraction of IP_3_Rs (Li & Rinzel, 1994)
$$\mathcal {H}$$: Heaviside function
$h_{\textrm{KA}}$: Gating variable for K_A_ inactivation
$h_{\textrm{Na}}$: Gating variable for Na^+^ inactivation
$h_{\textrm{NaP}}$: Gating variable for Na_P_ inactivation
$h_{\textrm{NaT}}$: Gating variable for Na_T_ inactivation
$h_{\textrm{phasic}}$: Gating variable for phasic channel inactivation
HW: Hardware
Hyper.: Hyperexcitability
I: Inhibitory
$$I_{\textrm{appl}}$$: Applied current
$I_{\textrm{ast}}$: Modulating current from the astrocyte to the neuron
$I_{\textrm{astro}}$: Modulating current from the astrocyte to the neuron depending on astrocytic Ca^2+^ (Nadkarni & Jung, 2003)
$I_{\textrm{Ca}}$: (Fast) Ca^2+^ current
$$I_{\textrm{CaT}}$$: Ca_T_ current
$I_{\textrm{const}}$: Applied constant current
$$I_{\textrm{coupling}}$$: Coupling current
$I_{\textrm{Gnoise}}$: Gaussian input noise current
$I_{\textrm{GWnoise}}$: Gaussian white noise current
$$I_{\textrm{K}}$$: K^+^ current
$$I_{\textrm{KA}}$$: K_A_ current
$$I_{\textrm{KCa}}$$: K_Ca_ current
$$I_{\textrm{KDR}}$$: K_DR_ current
$$I_{\textrm{Kleak}}$$: K^+^ leak current
$$I_{\textrm{Kpump}}$$: K^+^ pump current
$$I_{\textrm{leak}}$$: Leak current
$$I_{\textrm{Na}}$$: Na^+^ current
$I_{\textrm{Naleak}}$: Na^+^ leak current
$$I_{\textrm{NaP}}$$: Na_P_ current
$I_{\textrm{Napump}}$: Na^+^ pump current
$I_{\textrm{NaT}}$: Fast transient Na^+^ current
$I_{\textrm{noise}}$: Spatially incoherent exponentially correlated noise current
$$I_{\textrm{phasic}}$$: Phasic current
$I_{\textrm{Poisson}}$: Poisson input pulse current or Poisson spike train
$I_{\textrm{sAHP}}$: Slow AHP current
$I_{\textrm{slow}}$: Slowly varying current
$$I_{\textrm{syn}}$$: Synaptic current
$I_{\textrm{syn,KNMDAR}}$: K^+^ current via NMDAR
$I_{\textrm{syn,NaNMDAR}}$: Na^+^ current via NMDAR
$I_{\textrm{Tnoise}}$: Noise current from triangular distribution
$$I_{\textrm{tonic}}$$: Tonic current
$I_{\textrm{Unoise}}$: Uniformly distributed noise current
IN: Interneuron
Inf.: Information transfer
IP: Inositol polyphosphate
IP_3_: Inositol trisphosphate
IP_3_R: IP_3_ receptor
*k*: Constant
K^+^: Potassium ion
K_A_: Transient K^+^ channel
K_Ca_: Ca^2+^-activated K^+^ channel
K_DR_: Delayed rectifier K^+^ channel
L: Layer
LFP: Local field potential
LIF: Leaky integrate-and-fire
m: Motif
*M*: Number of cell types
$m_{\textrm{Na}}$: Gating variable for Na^+^ activation
$m_{\textrm{NaP}}$: Gating variable for Na_P_ activation
$m_{\textrm{NaT}}$: Gating variable for Na_T_ activation
$m_{\textrm{phasic}}$: Gating variable for phasic channel activation
MEA: Multi-electrode array
Mg^2+^: Magnesium ion
mGluR: Metabotropic Glu receptor
N: Neuron
$n_{\textrm{K}}$: Gating variable for K^+^ activation
$n_{\textrm{KA}}$: Gating variable for K_A_ activation
$n_{\textrm{KDR}}$: Gating variable for K_DR_ activation
Na^+^: Sodium ion
Na_P_: Persistent Na^+^ channel
Na_T_: Fast transient Na^+^ channel
NMDAR: N-methyl-D-aspartate receptor
NT: Neurotransmitter
*p*: Connection probability
*P*: Population of cells
$p_{\textrm{ast,rel}}$: Astrocytic release probability
$p_{\textrm{spike}}$: Probability for a spike
$p_{\textrm{syn,rel}}$: Synaptic release probability
PIP_2_: Phosphatidylinositol 4,5-bisphosphate
Plast.: Synaptic plasticity
PLC: Phospholipase C
PLC$\beta$: PLC isotype $\beta$
PLC$\delta$: PLC isotype $\delta$
PMCA: Plasma membrane Ca^2+^-ATPase
post: Postsynaptic
pre: Presynaptic
PY: Pyramidal
$q_{\textrm{sAHP}}$: Gating variable for slow AHP activation
$R_{\textrm{ac}}$: Fraction of IP_3_Rs that are not inactivated by Ca^2+^ (marked as *h* by Liu and Li (2013a) and *q* by Liu and Li (2013b), and same equation as by Allegrini et al. (2009), modified from Li and Rinzel (1994) and Höfer et al. (2002))
$R_{\textrm{in}}$: Fraction of IP_3_Rs that are not activated by Ca^2+^ (marked as *h* by Allegrini et al. (2009) and same equation as by Liu and Li (2013a, b), modified from Li and Rinzel (1994) and Höfer et al. (2002))
RE: Reticular thalamic
Rec: Recurrent
*S*: Matrix of synaptic connection weights
$s_{\textrm{AMPAR}}$: Fraction of AMPARs in open state
$S_{\textrm{ast}}$: State of astrocyte (active, inactive dormant state, or refractory)
$s_{\textrm{Ca}}$: Gating variable for Ca^2+^ activation
$s_{\textrm{E}}$: Gating variable for E synapse
$s_{\textrm{EE}}$: Gating variable for EE synapse
$s_{\textrm{EI}}$: Gating variable for EI synapse
$s_{\textrm{GABAAR}}$: Fraction of GABA_A_Rs in open state
$s_{\textrm{GABABR}}$: Fraction of GABA_B_Rs in open state
$s_{\textrm{I}}$: Gating variable for I synapse
$s_{\textrm{IE}}$: Gating variable for IE synapse
$s_{\textrm{II}}$: Gating variable for II synapse
$s_{\textrm{IN}}$: Gating variable for IN (interneuron) synapse
$$S_{\textrm{m}}$$: Secondary messenger
$s_{\textrm{mGluR}}$: Fraction of activated mGluRs
$s_{\textrm{NMDAR}}$: Fraction of NMDARs in open state
$s_{\textrm{PY}}$: Gating variable for PY synapse
$s_{\textrm{Rpre}}$: Fraction of activated presynaptic receptors
$s_{\textrm{syn}}$: Synaptic gating variable
SERCA: Sarco/ER Ca^2+^-ATPase
Sgn.: Signal transfer
soma: Somatic
syn: Synaptic cleft or synaptic
Sync.: Synchronization
TC: Thalamocortical
*u*: Membrane recovery variable (Izhikevich, 2003)
$u_{\textrm{syn}}$: Fraction of $x_{\textrm{syn}}$ docked for release
$V_{\textrm{AMPARpost}}$: Reversal potential of postsynaptic AMPAR current
$$V_{\textrm{m}}$$: Membrane potential
$V_{\textrm{NMDARpost}}$: Reversal potential of postsynaptic NMDAR current
*w*: Slow membrane recovery variable (FitzHugh, 1961)
*W*: Recovery variable showing the probability that K^+^ channel is conducting (Morris & Lecar, 1981)
$$W_{\textrm{syn}}$$: Synaptic weight
*X*: Hopf oscillator
$x_{\textrm{AMPAR}}$: Auxiliary variable for AMPAR
$x_{\textrm{ext}}$: Fraction of GT available for release
$x_{\textrm{GABAR}}$: Auxiliary variable for GABAR
$x_{\textrm{syn}}$: Fraction of NT available for release
*Y*: Hopf oscillator
*z*: Synaptic activation variable
*Z*: Synaptic activation variable
$z_0$: Reference level of *z*
