# Supplementary material for: Analysis of Network Models with Neuron-Astrocyte Interactions
Source: Neuroinformatics. 2023 Mar 23;21(2):375–406. doi: 10.1007/s12021-023-09622-w (PMC10085960; doi:10.1007/s12021-023-09622-w)
Supplement: Supplementary file 1 — Supplementary file1 (PDF 250 kb) [file 12021_2023_9622_MOESM1_ESM.pdf]

Supplementary material: Analysis of network models with neuron-astrocyte interactions

Tiina Manninen, Jugoslava Aćimović, and Marja-Leena Linne

January 24, 2023

Faculty of Medicine and Health Technology, Tampere University, Tampere, Finland
Emails: tiina.manninen@tuni.fi, marja-leena.linne@tuni.fi

We have provided three tables as supplementary material. Table S1 lists the model references used to build the neuron-astrocyte network models. Tables S2 and S3 characterize modeled neurons and astrocyte in the neuron-astrocyte network models, respectively.

Table S1: Model references used to build the neuron-astrocyte network models. This table shows the model references used to build the neurons and astrocytes as well as the interactions between neurons (NN), from neurons to astrocytes (NA), from astrocytes to neurons (AN), and between astrocytes (AA). Under Astrocytic references, we list only references related to astrocytes and, for example, the references related to gap junctions are under AA references.

**Study**	**Neuronal refs.**	**Astrocytic refs.**	**NN refs.**	**NA refs.**	**AN refs.**	**AA refs.**
Abed et al. (2020)	Izhikevich (2003)	Destexhe et al. (1994)	Izhikevich (2003)	Destexhe et al. (1994)	Postnov et al. (2007, 2009)	Ullah et al. (2006)
Aleksin et al. (2017)	Hodgkin & Huxley (1952); Kopell et al. (2010)	De Young & Keizer (1992); Li & Rinzel (1994); Nadkarni & Jung (2003); Volman et al. (2007)	Olufsen et al. (2003)	Tsodyks et al. (1998); Volman et al. (2007)	Volman et al. (2007)	Ullah et al. (2006)
Allegrini et al. (2009)	Izhikevich (2003)	De Young & Keizer (1992); Atri et al. (1993); Höfer et al. (2002); Nadkarni & Jung (2003)	Izhikevich (2003)	Nadkarni & Jung (2003)	Nadkarni & Jung (2003)	Sneyd et al. (1995); Höfer et al. (2002)
Amiri et al. (2012a)	Morris & Lecar (1981); Volman et al. (2007)	De Young & Keizer (1992); Li & Rinzel (1994); Nadkarni & Jung (2003); Volman et al. (2007)	Destexhe et al. (1994, 1998); Terman et al. (2002)	Destexhe et al. (1994); Terman et al. (2002); Volman et al. (2007)	Volman et al. (2007)	Ullah et al. (2006)
Amiri et al. (2012b)	Suffczynski et al. (2004)	Dupont & Goldbeter (1993); Sneyd et al. (1994); Kopell et al. (2000); Postnov et al. (2007, 2009)	Suffczynski et al. (2004)	Suffczynski et al. (2004); Postnov et al. (2007, 2009)	Postnov et al. (2007, 2009); Amiri et al. (2011)	Ullah et al. (2006)
Amiri et al. (2012c)	Suffczynski et al. (2004)	Dupont & Goldbeter (1993); Sneyd et al. (1994); Kopell et al. (2000); Postnov et al. (2007, 2009)	Suffczynski et al. (2004)	Suffczynski et al. (2004); Postnov et al. (2007, 2009)	Postnov et al. (2007, 2009); Amiri et al. (2011)	Ullah et al. (2006)
Amiri et al. (2013a)	Morris & Lecar (1981); Volman et al. (2007)	De Young & Keizer (1992); Li & Rinzel (1994); Nadkarni & Jung (2003); Volman et al. (2007)	Destexhe et al. (1994, 1998); Terman et al. (2002)	Destexhe et al. (1994); Terman et al. (2002); Volman et al. (2007)	Volman et al. (2007); Amiri et al. (2012a)	Ullah et al. (2006)
Chan et al. (2017)	Morris & Lecar (1981); Prescott et al. (2008)	De Young & Keizer (1992); Li & Rinzel (1994); Nadkarni & Jung (2003); Volman et al. (2007)	Latham et al. (2000)	Nadkarni & Jung (2003)	Volman et al. (2007); Amiri et al. (2012a)	Ullah et al. (2006)
Gordleeva et al. (2019)	Hodgkin & Huxley (1952); Esir et al. (2018)	De Young & Keizer (1992); Li & Rinzel (1994); Höfer et al. (2002); De Pittà et al. (2009); Gordleeva et al. (2018)	Tsodyks et al. (1998); Gordleeva et al. (2012)	Tsodyks et al. (1998); De Pittà et al. (2009); De Pittà & Brunel (2016); Gordleeva et al. (2012)	Gordleeva et al. (2012)	Ullah et al. (2006); Kazantsev (2009); Li et al. (2016); Gordleeva et al. (2018)
Haghiri et al. (2016)	Izhikevich (2003)	Dupont & Goldbeter (1993); Sneyd et al. (1994); Kopell et al. (2000); Postnov et al. (2007, 2009)	Kopell et al. (2000); Postnov et al. (2007, 2009)	Kopell et al. (2000); Postnov et al. (2007, 2009)	Kopell et al. (2000); Postnov et al. (2007, 2009)	n/a
Haghiri et al. (2017)	Izhikevich (2003)	Dupont & Goldbeter (1993); Sneyd et al. (1994); Kopell et al. (2000); Postnov et al. (2007, 2009)	Kopell et al. (2000); Postnov et al. (2007, 2009)	Kopell et al. (2000); Postnov et al. (2007, 2009)	Kopell et al. (2000); Postnov et al. (2007, 2009)	None
Haghiri & Ahmadi (2020)	Izhikevich (2003)	Dupont & Goldbeter (1993); Sneyd et al. (1994); Kopell et al. (2000); Postnov et al. (2007, 2009)	Kopell et al. (2000); Postnov et al. (2007, 2009)	Kopell et al. (2000); Postnov et al. (2007, 2009)	Kopell et al. (2000); Postnov et al. (2007, 2009)	None
Hayati et al. (2016)	FitzHugh (1961)	Dupont & Goldbeter (1993); Sneyd et al. (1994); Kopell et al. (2000); Postnov et al. (2007, 2009)	Kopell et al. (2000); Postnov et al. (2007, 2009)	Kopell et al. (2000); Postnov et al. (2007, 2009)	Kopell et al. (2000); Postnov et al. (2007, 2009)	n/a
Kanakov et al. (2019)	Hodgkin & Huxley (1952)	De Young & Keizer (1992); Dupont & Goldbeter (1993); Li & Rinzel (1994); Höfer et al. (2002); Nadkarni & Jung (2003, 2005); Ullah et al. (2006)	Destexhe et al. (1994, 1998)	n/a	Volman et al. (2007); De Pittà et al. (2011); Gordleeva et al. (2012)	Kazantsev (2009)

Continued on next page

Table S1: Model references used to build the neuron-astrocyte network models – Continued

Study	Neuronal refs.	Astrocytic refs.	NN refs.	NA refs.	AN refs.	AA refs.
Lenk et al. (2020)	Lenk et al. (2016)	De Young & Keizer (1992); Li & Rinzel (1994); Nadkarni & Jung (2003); Volman et al. (2007); De Pittà et al. (2008); Lallouette et al. (2014)	Tsodyks et al. (1998); De Pittà et al. (2011)	Tsodyks et al. (1998); Volman et al. (2007)	De Pittà et al. (2011); Lenk et al. (2016)	Lallouette et al. (2014)
Li et al. (2016)	Hodgkin & Huxley (1952); Ma et al. (2012)	De Young & Keizer (1992); Li & Rinzel (1994); Nadkarni & Jung (2003); Volman et al. (2007); Sahlender et al. (2014)	Destexhe et al. (1994, 1998); Terman et al. (2002)	Destexhe et al. (1994); Terman et al. (2002); Volman et al. (2007)	Postnov et al. (2007, 2009)	Ullah et al. (2006); Kazantsev (2009)
Li et al. (2020)	Jahr & Stevens (1990); Destexhe et al. (1994); Gerstner & Kistler (2002); Silchenko & Tass (2008)	De Young & Keizer (1992); Li & Rinzel (1994); Nadkarni & Jung (2003, 2005); Ullah et al. (2006); Li et al. (2017)	Destexhe et al. (1998); Tsodyks et al. (1998); Terman et al. (2002); De Pittà et al. (2011); De Pittà & Brunel (2016)	Tsodyks et al. (1998); Nadkarni & Jung (2005); De Pittà & Brunel (2016)	Destexhe et al. (1998); Tsodyks et al. (1998); De Pittà & Brunel (2016)	Goldberg et al. (2010)
Liu & Li (2013a)	Gerstner & Kistler (2002); Guo & Li (2011)	De Young & Keizer (1992); Atri et al. (1993); Dupont & Goldbeter (1993); Li & Rinzel (1994); Höfer et al. (2002); Nadkarni & Jung (2003, 2005); Ullah et al. (2006); Allegrini et al. (2009)	Guo & Li (2011)	Nadkarni & Jung (2005)	Nadkarni & Jung (2003)	Sneyd et al. (1995); Höfer et al. (2002); Allegrini et al. (2009)
Liu & Li (2013b)	Hodgkin & Huxley (1952); Gerstner & Kistler (2002)	De Young & Keizer (1992); Atri et al. (1993); Dupont & Goldbeter (1993); Li & Rinzel (1994); Höfer et al. (2002); Nadkarni & Jung (2003); Ullah et al. (2006); Allegrini et al. (2009)	Gerstner & Kistler (2002)	Nadkarni & Jung (2003)	Nadkarni & Jung (2003)	Sneyd et al. (1995); Höfer et al. (2002); Allegrini et al. (2009)
Liu et al. (2016)	Gerstner & Kistler (2002); Wade et al. (2012)	De Young & Keizer (1992); Li & Rinzel (1994); Nadkarni & Jung (2003); Volman et al. (2007); Wade et al. (2012); Naeem et al. (2015)	Volman et al. (2007); Wade et al. (2012)	Volman et al. (2007); Wade et al. (2012)	Tsodyks et al. (1998); Wade et al. (2012)	Ullah et al. (2006); Goldberg et al. (2010)
Makovkin et al. (2020)	Hodgkin & Huxley (1952); Mainen et al. (1995); Mainen & Sejnowski (1996)	De Young & Keizer (1992); Dupont & Goldbeter (1993); Li & Rinzel (1994); Höfer et al. (2002); Nadkarni & Jung (2003, 2005); Ullah et al. (2006)	Kanakov et al. (2019)	Tsodyks et al. (1998); Gordleeva et al. (2019)	Kanakov et al. (2019)	Kazantsev (2009)
Mesiti et al. (2015)	Pinsky & Rinzel (1994)	De Young & Keizer (1992); Sneyd et al. (1994); Nadkarni & Jung (2003); Larter & Craig (2005); Silchenko & Tass (2008)	Destexhe et al. (1994, 1998)	Nadkarni & Jung (2003)	Destexhe et al. (1994, 1998); Nadkarni & Jung (2003, 2007)	Ullah et al. (2006)
Naeem et al. (2015)	Gerstner & Kistler (2002); Wade et al. (2012)	De Young & Keizer (1992); Li & Rinzel (1994); Nadkarni & Jung (2003); Volman et al. (2007); Wade et al. (2012)	Volman et al. (2007); Wade et al. (2012)	Volman et al. (2007); Wade et al. (2012)	Tsodyks et al. (1998); Wade et al. (2012)	Ullah et al. (2006); Goldberg et al. (2010)
Nazari & Faez (2019)	Izhikevich (2003)	De Young & Keizer (1992); Li & Rinzel (1994); Nadkarni & Jung (2003); Volman et al. (2007)	Mazzoni et al. (2008)	Destexhe et al. (1994); Terman et al. (2002); Volman et al. (2007)	Postnov et al. (2007, 2009); Amiri et al. (2013b)	Ullah et al. (2006)
Nazari et al. (2020)	Gerstner & Kistler (2002); Mazzoni et al. (2008)	De Young & Keizer (1992); Li & Rinzel (1994); Nadkarni & Jung (2003); Volman et al. (2007)	Mazzoni et al. (2008)	Destexhe et al. (1994); Terman et al. (2002); Volman et al. (2007); Nazari & Faez (2019)	Postnov et al. (2007, 2009); Amiri et al. (2013b)	Ullah et al. (2006)
Postnov et al. (2009)	FitzHugh (1961)	Dupont & Goldbeter (1993); Sneyd et al. (1994); Kopell et al. (2000); Postnov et al. (2007)	Kopell et al. (2000); Postnov et al. (2007)	Kopell et al. (2000); Postnov et al. (2007)	Kopell et al. (2000); Postnov et al. (2007)	Ullah et al. (2006)

Continued on next page

Table S1: Model references used to build the neuron-astrocyte network models – Continued

**Study**	**Neuronal refs.**	**Astrocytic refs.**	**NN refs.**	**NA refs.**	**AN refs.**	**AA refs.**
Soleimani et al. (2015)	Reddy et al. (2000)	Dupont & Goldbeter (1993); Sneyd et al. (1994); Postnov et al. (2007, 2009)	Kopell et al. (2000); Postnov et al. (2007, 2009)	Postnov et al. (2007, 2009)	Postnov et al. (2007, 2009); Amiri et al. (2013b)	None
Stimberg et al. (2019)	Dayan & Abbott (2001)	De Young & Keizer (1992); Li & Rinzel (1994); Höfer et al. (2002); Shuai & Jung (2002); De Pittà et al. (2009); Goldberg et al. (2010); Wallach et al. (2014)	Tsodyks et al. (1998); Dayan & Abbott (2001); De Pittà et al. (2011)	Tsodyks et al. (1998); De Pittà et al. (2009); De Pittà & Brunel (2016)	Tsodyks et al. (1998); De Pittà et al. (2011); De Pittà & Brunel (2016)	Lallouette et al. (2014)
Tang et al. (2017)	Izhikevich (2003)	De Young & Keizer (1992); Li & Rinzel (1994); Höfer et al. (2002); Nadkarni & Jung (2003); Allegrini et al. (2009); De Pittà et al. (2009)	Izhikevich (2003)	Allegrini et al. (2009)	Nadkarni & Jung (2003)	Goldberg et al. (2010)
Yang & Yeo (2015)	n/a	De Young & Keizer (1992); Li & Rinzel (1994); Nadkarni & Jung (2003); Volman et al. (2007); De Pittà et al. (2009)	n/a	Volman et al. (2007)	n/a	n/a
Yao et al. (2018)	Hodgkin & Huxley (1952); Kager et al. (2000); Yao et al. (2011)	De Young & Keizer (1992); Li & Rinzel (1994); Fink et al. (1999); Kager et al. (2000); Lemon et al. (2003); Bennett et al. (2005); Somjen et al. (2008)	Destexhe et al. (1998); Bennett et al. (2008); Yao et al. (2011)	Yao et al. (2011)	Bennett et al. (2008); Yao et al. (2011)	Lemon et al. (2003); Bennett et al. (2005, 2008)
Yu et al. (2020)	Morris & Lecar (1981); Volman et al. (2007)	De Young & Keizer (1992); Li & Rinzel (1994); Nadkarni & Jung (2003); Volman et al. (2007)	Destexhe et al. (1994, 1998); Terman et al. (2002)	Destexhe et al. (1994); Terman et al. (2002); Volman et al. (2007)	Volman et al. (2007); Amiri et al. (2012a)	Ullah et al. (2006); Kazantsev (2009); Li et al. (2016)

Table S2: Characteristics of modeled neurons in the neuron-astrocyte network models. This table shows the inputs to the neurons, neuronal variables and other variables representing, for example, molecules released from neurons described by differential equations, as well as neuronal ionic currents and outputs of the neurons. We did not highlight the components, such as V_{m}, as neuronal unless we especially meant that they are, for example, either pre- or postsynaptic components. Amounts modeled in concentrations are given inside square brackets. We did not separate I_{syn} here into EE, EI, IE, and II connections. Note that synaptic conductance can be written as $g_{\mathrm{syn}} = \bar{g}_{\mathrm{syn}} s_{\mathrm{syn}}$, where synaptic gating variable s_{syn} can be, for example, s_{EE}. $I_{\mathrm{astro}} = 2.11\mathcal{H}(\ln(\Delta\mathrm{Ca}))\ln(\Delta\mathrm{Ca})$, where $\mathcal{H}$ is the heaviside function and $\Delta\mathrm{Ca} = [\mathrm{Ca}^{2+}]_{\mathrm{ast}} - 196.69(\mathrm{nM})$ (Nadkarni & Jung, 2003).

**Study**	**Inputs**	**Variables**	**Ionic currents**	**Outputs**
Abed et al. (2020)	$I_{\mathrm{ast}} \to V_{\mathrm{m,post}}$ ($\mathrm{Glu}_{\mathrm{ext}}$), $S \to V_{\mathrm{m,post}}$	u, V_{m}	None	NT, V_{m}
Aleksin et al. (2017)	$[\mathrm{Ca}^{2+}]_{\mathrm{ast}} \to p_{\mathrm{syn,rel}}$, $I_{\mathrm{appl}} \to V_{\mathrm{m}}$, $I_{\mathrm{phasic}} \to V_{\mathrm{m}}$, $I_{\mathrm{syn}} \to V_{\mathrm{m,post}}$, $I_{\mathrm{tonic}} \to V_{\mathrm{m}}$	g_{tonic}, h_{Na}, h_{phasic}, m_{Na}, m_{phasic}, n_{K}, [NT], s_{EE}, s_{EI}, s_{IE}, s_{II}, V_{m}, W_{syn}	I_{K}, I_{Na}, I_{leak}	I_{syn}, [NT]
Allegrini et al. (2009)	$I_{\mathrm{astro}} \to V_{\mathrm{m,post}}$, $S \to V_{\mathrm{m,post}}$	u, V_{m}	None	V_{m}
Amiri et al. (2012a)	$I_{\mathrm{ast}} \to V_{\mathrm{m,PY/IN}}$, $I_{\mathrm{const}} \to V_{\mathrm{m}}$, $I_{\mathrm{noise}} \to V_{\mathrm{m}}$, $I_{\mathrm{syn}} \to V_{\mathrm{m,post}}$ (NT)	I_{slow}, s_{E}, s_{I}, V_{m}, $W \approx n_{\mathrm{K}}$	I_{Ca}, I_{KDR}, I_{leak}, I_{slow}	I_{syn}, NT
Amiri et al. (2012b)	$G_{\mathrm{m}} \to C_{\mathrm{gain}}$, $I_{\mathrm{GWnoise}} \to V_{\mathrm{m}}$, $I_{\mathrm{syn,AMPAR/GABAAR/GABABR}} \to V_{\mathrm{m,post}}$	F, s_{AMPAR}, s_{GABAAR}, s_{GABABR}, V_{m}	I_{CaT}, I_{K}, I_{Na}, I_{leak}	F_{PY}, $I_{\mathrm{syn,AMPAR}}$, $I_{\mathrm{syn,GABAAR}}$, $I_{\mathrm{syn,GABABR}}$
Amiri et al. (2012c)	$G_{\mathrm{m}} \to C_{\mathrm{gain}}$, $I_{\mathrm{GWnoise}} \to V_{\mathrm{m}}$, $I_{\mathrm{syn,AMPAR/GABAAR/GABABR}} \to V_{\mathrm{m,post}}$	F, s_{AMPAR}, s_{GABAAR}, s_{GABABR}, V_{m}	I_{CaT}, I_{K}, I_{Na}, I_{leak}	F_{PY}, $I_{\mathrm{syn,AMPAR}}$, $I_{\mathrm{syn,GABAAR}}$, $I_{\mathrm{syn,GABABR}}$
Amiri et al. (2013a)	$I_{\mathrm{ast}} \to V_{\mathrm{m,PY/IN}}$, $I_{\mathrm{const}} \to V_{\mathrm{m}}$, $I_{\mathrm{noise}} \to V_{\mathrm{m}}$, $I_{\mathrm{syn}} \to V_{\mathrm{m,post}}$ (NT)	I_{slow}, s_{E}, s_{I}, V_{m}, $W \approx n_{\mathrm{K}}$	I_{Ca}, I_{KDR}, I_{leak}, I_{slow}	I_{syn}, NT
Chan et al. (2017)	$I_{\mathrm{ast}} \to V_{\mathrm{m,E/I}}$, $I_{\mathrm{syn}} \to V_{\mathrm{m,post}}$, $I_{\mathrm{Unoise}} \to V_{\mathrm{m}}$	s_{EE}, s_{EI}, s_{IE}, s_{II}, V_{m}, $W \approx n_{\mathrm{K}}$, q_{sAHP}	I_{K}, I_{Na}, I_{sAHP}, I_{leak}	I_{syn}, V_{m}
Gordleeva et al. (2019)	$I_{\mathrm{const}} \to V_{\mathrm{m}}$, $I_{\mathrm{Poisson}} \to V_{\mathrm{m}}$, $I_{\mathrm{syn,NMDAR}} \to V_{\mathrm{m,post}}$ ($\mathrm{D\text{-}serine}_{\mathrm{ext}}$, $\mathrm{Glu}_{\mathrm{ext}}$, $\mathrm{Glu}_{\mathrm{syn}}$)	$\mathrm{Glu}_{\mathrm{syn}}$, h_{Na}, $I_{\mathrm{syn,NMDAR}}$, m_{Na}, n_{K}, V_{m}	I_{K}, I_{Na}, I_{leak}	$\mathrm{Glu}_{\mathrm{syn}}$, $I_{\mathrm{syn,NMDAR}}$
Haghiri et al. (2016)	$I_{\mathrm{appl}} \to V_{\mathrm{m,pre}}$, $I_{\mathrm{ast}} \to V_{\mathrm{m,post}}$, $I_{\mathrm{syn}} \to V_{\mathrm{m,post}}$	u, V_{m}, z	None	I_{syn}, u, z
Haghiri et al. (2017)	$I_{\mathrm{appl}} \to V_{\mathrm{m,pre}}$, $I_{\mathrm{ast,ATP/Glu}} \to V_{\mathrm{m,post}}$, $I_{\mathrm{syn}} \to V_{\mathrm{m,post}}$	u, V_{m}, z	None	I_{syn}, u, z
Haghiri & Ahmadi (2020)	$I_{\mathrm{appl}} \to V_{\mathrm{m,pre}}$, $I_{\mathrm{ast}} \to V_{\mathrm{m,post}}$, $I_{\mathrm{syn}} \to V_{\mathrm{m,post}}$	u, V_{m}, z	None	I_{syn}, u, z
Hayati et al. (2016)	$I_{\mathrm{appl}} \to V_{\mathrm{m,pre}}$, $I_{\mathrm{ast}} \to V_{\mathrm{m,post}}$, $I_{\mathrm{syn}} \to V_{\mathrm{m,post}}$	V_{m}, w, z	None	I_{syn}, w, z
Kanakov et al. (2019)	$[\mathrm{Ca}^{2+}]_{\mathrm{ast}} \to g_{\mathrm{syn}}$, $I_{\mathrm{const}} \to V_{\mathrm{m}}$, $I_{\mathrm{Poisson}} \to V_{\mathrm{m}}$, $I_{\mathrm{syn}} \to V_{\mathrm{m,post}}$	h_{Na}, m_{Na}, n_{K}, V_{m}	I_{K}, I_{Na}, I_{leak}	I_{syn}
Lenk et al. (2020)	$[\mathrm{Ca}^{2+}]_{\mathrm{ast}} \to s_{\mathrm{Rpre}} \to p_{\mathrm{syn,rel}}$, $I_{\mathrm{syn}} \to F_{\mathrm{post}}$ (NT), $I_{\mathrm{Tnoise}} \to F$, $S_{\mathrm{ast}} \to F_{\mathrm{post}}$	p_{spike}, s_{Rpre}, u_{syn}, x_{syn}	None	NT
Li et al. (2016)	$I_{\mathrm{ast,ATP}} \to V_{\mathrm{m,PY}}$ ($[\mathrm{ATP}]_{\mathrm{ext}}$), $I_{\mathrm{ast,Glu}} \to V_{\mathrm{m,IN}}$ ($[\mathrm{Glu}]_{\mathrm{ext}}$), $I_{\mathrm{const}} \to V_{\mathrm{m}}$, $I_{\mathrm{syn}} \to V_{\mathrm{m,post}}$ (NT)	h_{Na}, m_{Na}, n_{K}, s_{IN}, s_{PY}, V_{m}	I_{K}, I_{Na}, I_{leak}	I_{syn}, NT
Li et al. (2020)	$[\mathrm{GABA}]_{\mathrm{ext}} \to s_{\mathrm{GABABRpre}} \to p_{\mathrm{syn,rel}}$, $[\mathrm{Glu}]_{\mathrm{ext}} \to s_{\mathrm{mGluRpre}} \to p_{\mathrm{syn,rel}}$, $I_{\mathrm{ast,AMPAR/NMDAR}} \to V_{\mathrm{m,post}}$ ($[\mathrm{Glu}]_{\mathrm{ext}}$), $I_{\mathrm{const}} \to V_{\mathrm{m}}$, $I_{\mathrm{syn,AMPAR/NMDAR}} \to V_{\mathrm{m,post}}$ ($[\mathrm{Glu}]_{\mathrm{syn}}$), $I_{\mathrm{syn,GABAAR}} \to V_{\mathrm{m,post}}$ ($[\mathrm{GABA}]_{\mathrm{syn}}$)	$[\mathrm{GABA}]_{\mathrm{syn}}$, $[\mathrm{Glu}]_{\mathrm{syn}}$, $s_{\mathrm{AMPARpost}}$ (separately for $[\mathrm{Glu}]_{\mathrm{ext}}$ and $[\mathrm{Glu}]_{\mathrm{syn}}$), $s_{\mathrm{GABAARpost}}$, $s_{\mathrm{GABABRpre}}$, s_{mGluRpre}, $s_{\mathrm{NMDARpost}}$ (separately for $[\mathrm{Glu}]_{\mathrm{ext}}$ and $[\mathrm{Glu}]_{\mathrm{syn}}$), u_{syn}, V_{m}, x_{syn}	I_{leak}	$[\mathrm{GABA}]_{\mathrm{syn}}$, $[\mathrm{Glu}]_{\mathrm{syn}}$, $I_{\mathrm{syn,AMPAR}}$, $I_{\mathrm{syn,GABAAR}}$, $I_{\mathrm{syn,NMDAR}}$

Continued on next page

Table S2: Characteristics of modeled neurons in the neuron-astrocyte network models – Continued

Study	Inputs	Variables	Ionic currents	Outputs
Liu & Li (2013a)	$I_{\mathrm{astro}} \rightarrow V_{\mathrm{m}}$, $I_{\mathrm{Gnoise}} \rightarrow V_{\mathrm{m,pre}}$, $I_{\mathrm{GWnoise}} \rightarrow V_{\mathrm{m}}$, $I_{\mathrm{syn}} \rightarrow V_{\mathrm{m,post}}$	s_{E}, s_{I}, V_{m}	None	$[\mathrm{Glu}]_{\mathrm{syn}}$, I_{syn}
Liu & Li (2013b)	$I_{\mathrm{appl}} \rightarrow V_{\mathrm{m}}$, $I_{\mathrm{astro}} \rightarrow V_{\mathrm{m}}$, $I_{\mathrm{GWnoise}} \rightarrow V_{\mathrm{m}}$, $I_{\mathrm{syn}} \rightarrow V_{\mathrm{m,post}}$	h_{Na}, m_{Na}, n_{K}, s_{E}, s_{I}, V_{m}	I_{K}, I_{Na}, I_{leak}	I_{syn}, V_{m}
Liu et al. (2016)	$[\text{2-AG}]_{\mathrm{post}} \rightarrow$ DSE $\rightarrow p_{\mathrm{syn,rel}}$, $[\mathrm{Glu}]_{\mathrm{ext}} \rightarrow$ e-SP $\rightarrow p_{\mathrm{syn,rel}}$, $I_{\mathrm{Poisson}} \rightarrow p_{\mathrm{syn,rel}} \rightarrow I_{\mathrm{syn}} \rightarrow V_{\mathrm{m,post}}$	$[\text{2-AG}]_{\mathrm{post}}$, e-SP, $V_{\mathrm{m,post}}$	None	$[\text{2-AG}]_{\mathrm{post}}$, I_{syn}
Makovkin et al. (2020)	$[\mathrm{Ca}^{2+}]_{\mathrm{ast}} \rightarrow g_{\mathrm{syn}}$, $I_{\mathrm{const}} \rightarrow V_{\mathrm{m}}$, $I_{\mathrm{syn}} \rightarrow V_{\mathrm{m,post}}$	h_{Na}, m_{Na}, n_{K}, NT, V_{m}	I_{K}, I_{Na}, I_{leak}	I_{syn}, NT
Mesiti et al. (2015)	$[\mathrm{Ca}^{2+}]_{\mathrm{ast}} \rightarrow [\mathrm{Ca}^{2+}]_{\mathrm{pre}}$, $[\mathrm{Ca}^{2+}]_{\mathrm{ast}} \rightarrow g_{\mathrm{syn,AMPAR/NMDAR}}$, $I_{\mathrm{ast,AMPAR/NMDAR}} \rightarrow V_{\mathrm{m,postdend}}$, $I_{\mathrm{astro}} \rightarrow V_{\mathrm{m,presoma}}$, $I_{\mathrm{const}} \rightarrow V_{\mathrm{m,dend/soma}}$, $I_{\mathrm{syn,AMPAR/NMDAR}} \rightarrow V_{\mathrm{m,postdend}}$	c_{KCa}, $[\mathrm{Ca}^{2+}]_{\mathrm{pre}}$, h_{Na}, n_{KDR}, q_{sAHP}, $s_{\mathrm{AMPARpost}}$ (separately for synaptic and astrocytic activation), s_{Ca}, $s_{\mathrm{NMDARpost}}$ (separately for synaptic and astrocytic activation), $V_{\mathrm{m,dend}}$, $V_{\mathrm{m,soma}}$	I_{Ca}, I_{KCa}, I_{KDR}, I_{Na}, I_{sAHP}, $I_{\mathrm{leak,dend}}$, $I_{\mathrm{leak,soma}}$, I_{coupling} (In addition for Ca^{2+} in pre N: degradation, production by $[\mathrm{Ca}^{2+}]_{\mathrm{ast}}$; in post N: production by F_{pre} and $V_{\mathrm{m,soma}}$)	$I_{\mathrm{syn,AMPAR}}$, $I_{\mathrm{syn,NMDAR}}$, $V_{\mathrm{m,soma}}$
Naeem et al. (2015)	$[\text{2-AG}]_{\mathrm{post}} \rightarrow$ DSE $\rightarrow p_{\mathrm{syn,rel}}$, $[\mathrm{Glu}]_{\mathrm{ext}} \rightarrow$ e-SP $\rightarrow p_{\mathrm{syn,rel}}$, $I_{\mathrm{Poisson}} \rightarrow p_{\mathrm{syn,rel}} \rightarrow I_{\mathrm{syn}} \rightarrow V_{\mathrm{m,post}}$	$[\text{2-AG}]_{\mathrm{post}}$, e-SP, $V_{\mathrm{m,post}}$	None	$[\text{2-AG}]_{\mathrm{post}}$, I_{syn}
Nazari & Faez (2019)	$I_{\mathrm{ast}} \rightarrow x_{\mathrm{AMPAR/GABAR}} \rightarrow I_{\mathrm{syn,AMPAR/GABAR}} \rightarrow V_{\mathrm{m,PY/IN}}$, $I_{\mathrm{Poisson}} \rightarrow V_{\mathrm{m}}$, $I_{\mathrm{syn,AMPAR/GABAR}} \rightarrow V_{\mathrm{m,post}}$	$I_{\mathrm{syn,AMPAR}}$, $I_{\mathrm{syn,GABAR}}$, u, V_{m}, x_{AMPAR}, x_{GABAR}	None	$I_{\mathrm{syn,AMPAR}}$, $I_{\mathrm{syn,GABAR}}$, NT
Nazari et al. (2020)	$I_{\mathrm{ast}} \rightarrow x_{\mathrm{AMPAR/GABAR}} \rightarrow I_{\mathrm{syn,AMPAR/GABAR}} \rightarrow V_{\mathrm{m,PY/IN}}$, $I_{\mathrm{Poisson}} \rightarrow V_{\mathrm{m}}$, $I_{\mathrm{syn,AMPAR/GABAR}} \rightarrow V_{\mathrm{m,post}}$	$I_{\mathrm{syn,AMPAR}}$, $I_{\mathrm{syn,GABAR}}$, V_{m}, x_{AMPAR}, x_{GABAR}	None	$I_{\mathrm{syn,AMPAR}}$, $I_{\mathrm{syn,GABAR}}$, NT
Postnov et al. (2009)	$I_{\mathrm{appl}} \rightarrow w_{\mathrm{pre}}$, $I_{\mathrm{ast,ATP/Glu}} \rightarrow w_{\mathrm{post}}$, $I_{\mathrm{const}} \rightarrow w$, $I_{\mathrm{syn}} \rightarrow w_{\mathrm{post}}$	V_{m}, w, z	None	I_{syn}, w, z
Soleimani et al. (2015)	$I_{\mathrm{ast}} \rightarrow X$, $I_{\mathrm{ast}} \rightarrow Y$, $I_{\mathrm{syn}} \rightarrow X_{\mathrm{post}}$, $I_{\mathrm{syn}} \rightarrow Y_{\mathrm{post}}$	X, Y	None	I_{syn}, Z
Stimberg et al. (2019)	[GT] $\rightarrow s_{\mathrm{Rpre}} \rightarrow p_{\mathrm{syn,rel}}$, $I_{\mathrm{const}} \rightarrow V_{\mathrm{m}}$, $I_{\mathrm{syn}} \rightarrow V_{\mathrm{m,post}}$	[NT], s_{E}, s_{I}, s_{Rpre}, u_{syn}, V_{m}, x_{syn}	I_{leak}	I_{syn}, [NT]
Tang et al. (2017)	$I_{\mathrm{astro}} \rightarrow V_{\mathrm{m}}$, $I_{\mathrm{Unoise}} \rightarrow V_{\mathrm{m}}$, $S \rightarrow V_{\mathrm{m,post}}$	u, V_{m}	None	V_{m}
Yang & Yeo (2015)	$[\mathrm{ATP}]_{\mathrm{ext}} \rightarrow \mathrm{NMDAR}_{\mathrm{post}}$, $[\mathrm{Glu}]_{\mathrm{ext}} \rightarrow \mathrm{NMDAR}_{\mathrm{post}}$, $[\mathrm{Glu}]_{\mathrm{syn}}$	n/a	n/a	$[\mathrm{Glu}]_{\mathrm{syn}}$
Yao et al. (2018)	$I_{\mathrm{appl}} \rightarrow V_{\mathrm{m}}$, $I_{\mathrm{syn,K/NaNMDAR}} \rightarrow V_{\mathrm{m,post}}$ ($[\mathrm{Glu}]_{\mathrm{ext}}$), $[\mathrm{K}^{+}]_{\mathrm{ext}}$, $[\mathrm{Na}^{+}]_{\mathrm{ext}}$	$[\mathrm{Glu}]_{\mathrm{ext}}$, h_{KA}, h_{NaP}, h_{NaT}, $[\mathrm{K}^{+}]$, m_{NaP}, m_{NaT}, n_{KA}, n_{KDR}, $[\mathrm{Na}^{+}]$, s_{NMDAR}, V_{m}	I_{KA}, I_{KDR}, I_{Kleak}, I_{Kpump}, I_{NaP}, I_{NaT}, I_{Naleak}, I_{Napump}, I_{leak}, $I_{\mathrm{syn,KNMDAR}}$, $I_{\mathrm{syn,NaNMDAR}}$	$[\mathrm{Glu}]_{\mathrm{ext}}$, $[\mathrm{K}^{+}]_{\mathrm{ext}}$, $[\mathrm{Na}^{+}]_{\mathrm{ext}}$, $I_{\mathrm{syn,KNMDAR}}$, $I_{\mathrm{syn,NaNMDAR}}$
Yu et al. (2020)	$I_{\mathrm{ast}} \rightarrow V_{\mathrm{m,PY/IN}}$, $I_{\mathrm{const}} \rightarrow V_{\mathrm{m}}$, $I_{\mathrm{noise}} \rightarrow V_{\mathrm{m}}$, $I_{\mathrm{syn}} \rightarrow V_{\mathrm{m,post}}$ (NT)	I_{slow}, s_{E}, s_{I}, V_{m}, $W \approx n_{\mathrm{K}}$	I_{Ca}, I_{KDR}, I_{leak}, I_{slow}	I_{syn}, NT

Table S3: Characteristics of modeled astrocytes in the neuron-astrocyte network models. This table shows the inputs to the astrocytes, astrocytic variables and other variables representing, for example, molecules released from astrocytes described by differential equations, as well as astrocytic Ca^{2+} mechanisms related to cytosolic Ca^{2+}, astrocytic IP_3 mechanisms, diffusion (Diff.) of astrocytic variables either in the cytosol or ER, and outputs of the astrocytes. Amounts modeled in concentrations are given inside square brackets. $I_{astro} = 2.11\mathcal{H}(\ln(\Delta Ca))\ln(\Delta Ca)$, where $\mathcal{H}$ is the heaviside function and $\Delta Ca = [Ca^{2+}] - 196.69(nM)$ (Nadkarni & Jung, 2003). In the models by Mesiti et al. (2015) and Li et al. (2020), $I_{ast,AMPAR} = \bar{g}_{AMPAR} s_{AMPAR}(V_{m,post} - V_{AMPARpost})$ and $I_{ast,NMDAR} = \bar{g}_{NMDAR} B_{Mg} s_{NMDAR}(V_{m,post} - V_{NMDARpost})$.

**Study**	**Inputs**	**Variables**	**Ca^{2+} mechanisms**	**IP_3 mechanisms**	**Diff.**	**Outputs**
Abed et al. (2020)	IP_3, NT $\rightarrow IP_3$	Ca^{2+}, IP_3	CICR, efflux	Degradation, GJ, production by NT	None	$I_{ast} = c\mathrm{Glu}_{ext}$, IP_3
Aleksin et al. (2017)	$[Ca^{2+}]$, [NT] $\rightarrow [IP_3]$	$[Ca^{2+}]$, h, $[IP_3]$	CICR, GJ, leak from ER into cyt, SERCA	Degradation, production by NT	None	$[Ca^{2+}]$
Allegrini et al. (2009)	$[Ca^{2+}]$, $[IP_3]$, $V_{m,pre} \rightarrow [IP_3]$	$[Ca^{2+}]$, $[IP_3]$, R_{in}	CICR, efflux via pump, GJ, SERCA	Degradation, GJ, production by $V_{m,pre}$	D_{cyt}: $[Ca^{2+}]$, $[IP_3]$	$[Ca^{2+}]$, I_{astro}, $[IP_3]$
Amiri et al. (2012a)	$[IP_3]$, NT $\rightarrow [IP_3]$	$[Ca^{2+}]$, f, h, $[IP_3]$	CICR, leak from ER into cyt, SERCA	Degradation, GJ, production by NT	None	$I_{ast} = cf$, $[IP_3]$
Amiri et al. (2012b)	$F_{PY} \rightarrow S_m$, S_m	Ca^{2+}, Ca^{2+}_{ER}, G_m, $S_m = IP_3$	CICR, efflux, influx, IP_3-dependent influx, leak from ER into cyt, SERCA	Degradation, GJ, production by F_{PY}	None	G_m, S_m
Amiri et al. (2012c)	$F_{PY} \rightarrow S_m$, S_m	Ca^{2+}, Ca^{2+}_{ER}, G_m, $S_m = IP_3$	CICR, efflux, influx, IP_3-dependent influx, leak from ER into cyt, SERCA	Degradation, GJ, production by F_{PY}	None	G_m, S_m
Amiri et al. (2013a)	$[IP_3]$, NT $\rightarrow [IP_3]$	$[Ca^{2+}]$, f, h, $[IP_3]$	CICR, leak from ER into cyt, SERCA	Degradation, GJ, production by NT	None	$I_{ast} = cf$, $[IP_3]$
Chan et al. (2017)	$[IP_3]$, $V_{m,N} \rightarrow [IP_3]$	$[Ca^{2+}]$, f, h, $[IP_3]$	CICR, leak from ER into cyt, SERCA	Degradation, GJ, production by $V_{m,N}$ (PLCβ), production by Ca^{2+} (PLCδ)	None	$I_{ast} = cf$, $[IP_3]$
Gordleeva et al. (2019)	$[Ca^{2+}]$, $\mathrm{Glu}_{syn} \rightarrow [IP_3]$, $[IP_3]$	$[Ca^{2+}]$, $[Ca^{2+}]_{ER}$, D-serine$_{ext}$, Glu_{ext}, h, $[IP_3]$	CICR, GJ, leak from ER into cyt, SERCA	Degradation by IP_3-3K, degradation by IP-5P, GJ, production by Glu (PLCβ), production by Ca^{2+} (PLCδ)	D_{cyt}: $[Ca^{2+}]$, $[IP_3]$; D_{ER}: $[Ca^{2+}]$	$[Ca^{2+}]$, D-serine$_{ext}$, Glu_{ext}, $[IP_3]$
Haghiri et al. (2016)	$u_{post} \rightarrow Ca^{2+}$, $z \rightarrow S_m$	Ca^{2+}, Ca^{2+}_{ER}, G_m, $S_m = IP_3$	CICR, efflux, influx, IP_3-dependent influx, leak from ER into cyt, SERCA, $V_{m,post}$-dependent influx	Degradation, production by z	None	$I_{ast} = cG_m$, $I_{syn} = (k - cG_m)(z - z_0)$
Haghiri et al. (2017)	$u_{post} \rightarrow Ca^{2+}$, $z \rightarrow S_m$	Ca^{2+}, Ca^{2+}_{ER}, $G_a = \mathrm{ATP}_{ext}$, $G_m = \mathrm{Glu}_{ext}$, $S_m = IP_3$	CICR, efflux, influx, IP_3-dependent influx, leak from ER into cyt, SERCA, $V_{m,post}$-dependent influx	Degradation, production by z	None	$I_{ast,ATP} = cG_a$, $I_{ast,Glu} = cG_m$, $I_{syn} = (k - cG_m)(z - z_0)$
Haghiri & Ahmadi (2020)	$u_{post} \rightarrow Ca^{2+}$, $z \rightarrow S_m$	Ca^{2+}, Ca^{2+}_{ER}, $G_m = \mathrm{Glu}_{ext}$, $S_m = IP_3$	CICR, efflux, influx, IP_3-dependent influx, leak from ER into cyt, SERCA, $V_{m,post}$-dependent influx	Degradation, production by z	None	$I_{ast} = cG_m$, $I_{syn} = (k - cG_m)(z - z_0)$
Hayati et al. (2016)	$w_{post} \rightarrow Ca^{2+}$, $z \rightarrow S_m$	Ca^{2+}, G_m, $S_m = IP_3$	Efflux, influx, IP_3-dependent influx, $V_{m,post}$-dependent influx	Degradation, production by z	None	$I_{ast} = cG_m$, $I_{syn} = (k - cG_m)(z - z_0)$
Kanakov et al. (2019)	$[Ca^{2+}]$, $[IP_3]$	$[Ca^{2+}]$, h, $[IP_3]$	CCE, CICR, efflux, GJ, leak from ER into cyt, leak from ext into cyt, SERCA	Degradation, GJ, production by Ca^{2+} (PLCδ)	None	$[Ca^{2+}]$, $[IP_3]$

Continued on next page

Table S3: Characteristics of modeled astrocytes in the neuron-astrocyte network models – Continued

Study	Inputs	Variables	Ca^{2+} mechanisms	IP_3 mechanisms	Diff.	Outputs
Lenk et al. (2020)	$[IP_3]$, NT $\rightarrow [IP_3]$	$[Ca^{2+}]$, $[IP_3]$	Accumulation rate	Degradation, GJ, production by NT	None	$[Ca^{2+}]$, $[IP_3]$, S_{ast}
Li et al. (2016)	$[IP_3]$, NT $\rightarrow [IP_3]$	$[ATP]_{ext}$, $[Ca^{2+}]$, $[Glu]_{ext}$, h, $[IP_3]$	CICR, leak from ER into cyt, SERCA	Degradation, GJ, production by NT	None	$I_{ast,ATP} = c[ATP]_{ext}$, $I_{ast,Glu} = c[Glu]_{ext}$, $[IP_3]$
Li et al. (2020)	$[GABA]_{ext} \rightarrow [IP_3]$, $[Glu]_{syn} \rightarrow [IP_3]$, $[IP_3]$	$[Ca^{2+}]$, $[Glu]_{ext}$, h, $[IP_3]$, x_{ext}	CICR, leak from ER into cyt, SERCA	Degradation, GJ, production by GABA, production by Glu	None	$[Glu]_{ext}$, $I_{ast,AMPAR}$, $I_{ast,NMDAR}$, $[IP_3]$
Liu & Li (2013a)	$[Ca^{2+}]$, $[Glu]_{syn} \rightarrow [IP_3]$, $[IP_3]$	$[Ca^{2+}]$, $[IP_3]$, R_{ac}	CICR, efflux via pump, GJ, leak from ER into cyt, SERCA	Degradation, GJ, production by Ca^{2+} (PLCδ), production by Glu	D_{cyt}: $[Ca^{2+}]$, $[IP_3]$	$[Ca^{2+}]$, I_{astro}, $[IP_3]$
Liu & Li (2013b)	$[Ca^{2+}]$, $[IP_3]$, $V_{m,pre} \rightarrow [IP_3]$	$[Ca^{2+}]$, $[IP_3]$, R_{ac}	CICR, efflux via pump, GJ, leak from ER into cyt, SERCA	Degradation, GJ, production by Ca^{2+} (PLCδ), production by $V_{m,pre}$	D_{cyt}: $[Ca^{2+}]$, $[IP_3]$	$[Ca^{2+}]$, I_{astro}, $[IP_3]$
Liu et al. (2016)	$[2\text{-}AG]_{post} \rightarrow [IP_3]$, $[IP_3]$	$[Ca^{2+}]$, $[Glu]_{ext}$, h, $[IP_3]$	CICR, leak from ER into cyt, SERCA	Degradation, GJ, production by 2-AG	None	$[Glu]_{ext}$, $[IP_3]$
Makovkin et al. (2020)	$[Ca^{2+}]$, $[IP_3]$, NT $\rightarrow [IP_3]$	$[Ca^{2+}]$, h, $[IP_3]$	CCE, CICR, efflux, GJ, leak from ER into cyt, leak from ext into cyt, SERCA	Degradation, GJ, production by Ca^{2+} (PLCδ), production by NT	None	$[Ca^{2+}]$, $[IP_3]$
Mesiti et al. (2015)	$[Ca^{2+}]$, $[IP_3]$, $V_{m,presoma} \rightarrow [IP_3]$	$[Ca^{2+}]$, $[Ca^{2+}]_{ER}$, $[IP_3]$	CICR, efflux, influx, IP_3-dependent influx, leak from ER into cyt, SERCA	Degradation, GJ, production by $V_{m,presoma}$	D_{cyt}: $[Ca^{2+}]$, $[IP_3]$	$[Ca^{2+}]$, $I_{ast,AMPAR}$, $I_{ast,NMDAR}$, I_{astro}, $[IP_3]$
Naeem et al. (2015)	$[2\text{-}AG]_{post} \rightarrow [IP_3]$, $[IP_3]$	$[Ca^{2+}]$, $[Glu]_{ext}$, h, $[IP_3]$	CICR, leak from ER into cyt, SERCA	Degradation, GJ, production by 2-AG	None	$[Glu]_{ext}$, $[IP_3]$
Nazari & Faez (2019)	$[IP_3]$, NT $\rightarrow [IP_3]$	$[Ca^{2+}]$, h, $[IP_3]$	CICR, leak from ER into cyt, SERCA	Degradation, GJ, production by NT	None	$I_{ast} = c[Ca^{2+}]$, $[IP_3]$
Nazari et al. (2020)	$[IP_3]$, NT $\rightarrow [IP_3]$	$[Ca^{2+}]$, h, $[IP_3]$	CICR, leak from ER into cyt, SERCA	Degradation, GJ, production by NT	None	$I_{ast} = c[Ca^{2+}]$, $[IP_3]$
Postnov et al. (2009)	Ca^{2+}, G_a, G_m, S_m, $w_{post} \rightarrow Ca^{2+}$, $z \rightarrow S_m$	Ca^{2+}, Ca^{2+}_{ER}, $G_a = ATP_{ext}$, $G_m = Glu_{ext}$, $S_m = IP_3$	CICR, efflux, GJ, influx, IP_3-dependent influx, leak from ER into cyt, SERCA, $V_{m,post}$-dependent influx	Degradation, GJ, production by z	D_{cyt}: $[Ca^{2+}]$, $[IP_3]$	Ca^{2+}, $I_{ast,ATP} = cG_a$, $I_{ast,Glu} = cG_m$, $I_{syn} = (k - cG_m)(z - z_0)$, S_m
Soleimani et al. (2015)	$Z \rightarrow S_m$	Ca^{2+}, $S_m = IP_3$	Efflux, influx, IP_3-dependent influx	Degradation, production by Z	None	$I_{ast} = cCa^{2+}$
Stimberg et al. (2019)	$[IP_3]$, $[NT] \rightarrow s_{mGluR} \rightarrow [IP_3]$	$[Ca^{2+}]$, $[GT]$, h, $[IP_3]$, s_{mGluR}, x_{ext}	CICR, leak from ER into cyt, SERCA	Degradation by IP_3-3K, degradation by IP-5P, GJ, production by exogenous flow, production by NT (PLCβ), production by Ca^{2+} (PLCδ)	None	$[GT]$, $[IP_3]$
Tang et al. (2017)	$[IP_3]$, $V_{m,N} \rightarrow [IP_3]$	$[Ca^{2+}]$, h, $[IP_3]$	CICR, leak from ER into cyt, SERCA	Degradation by IP_3-3K, degradation by IP-5P, GJ, production by exogenous flow, production by Ca^{2+} (PLCδ), production by $V_{m,N}$	None	I_{astro}, $[IP_3]$
Yang & Yeo (2015)	$[ATP]_{ext}$, $[Glu]_{ext}$, $[Glu]_{syn} \rightarrow [IP_3]$, $[IP_3]$	$[ATP]_{ext}$, $[Ca^{2+}]$, $[Glu]_{ext}$, h, $[IP_3]$	CICR, leak from ER into cyt, SERCA	GJ, production by Glu	None	$[ATP]_{ext}$, $[Glu]_{ext}$, $[IP_3]$

Continued on next page

Table S3: Characteristics of modeled astrocytes in the neuron-astrocyte network models – Continued

Study	Inputs	Variables	Ca^{2+} mechanisms	IP_3 mechanisms	Diff.	Outputs
Yao et al. (2018)	$[ATP]_{ext}$, $[K^+]_{ext}$, $[Na^+]_{ext}$	χ, $[ATP]_{ext}$, $[Ca^{2+}]$, $[Ca^{2+}]_{ER}$, G, $[Glu]_{ext}$, h, h_{NaP}, h_{NaT}, $[IP_3]$, $[K^+]$, $[K^+]_{ext}$, m_{NaP}, m_{NaT}, n_{KDR}, $[Na^+]$, $[Na^+]_{ext}$, V_m	Buffer, CICR, leak from ER into cyt, leak from ext into cyt, PMCA, SERCA (In addition for K^+ and Na^+: I_{KDR}, I_{Kleak}, I_{Kpump}, I_{NaP}, I_{NaT}, I_{Naleak}, I_{Napump}, I_{leak})	Degradation, production by Ca^{2+}, G protein, and PIP_2	None	$[ATP]_{ext}$, $[Glu]_{ext}$, $[K^+]_{ext}$, $[Na^+]_{ext}$
Yu et al. (2020)	$[IP_3]$, NT $\rightarrow$ $[IP_3]$	$[Ca^{2+}]$, f, h, $[IP_3]$	CICR, leak from ER into cyt, SERCA	Degradation, GJ, production by NT	None	$I_{ast} = c \sum f$, $[IP_3]$

References

Abed, B., Ayyoub, B., Ismail, A. R., & Abdul, N. (2020). Two suggested probabilistic and kinetic models for astrocytic network in spiking neural networks. *International Journal of Advanced Trends in Computer Science and Engineering*, 9(5), 7951–7957. https://doi.org/10.30534/ijatcse/2020/149952020

Aleksin, S. G., Zheng, K., Rusakov, D. A., & Savtchenko, L. P. (2017). ARACHNE: A neural-neuroglial network builder with remotely controlled parallel computing. *PLoS Computational Biology*, 13(3), e1005467. https://doi.org/10.1371/journal.pcbi.1005467

Allegrini, P., Fronzoni, L., & Pirino, D. (2009). The influence of the astrocyte field on neuronal dynamics and synchronization. *Journal of Biological Physics*, 35(4), 413–423. https://doi.org/10.1007/s10867-009-9166-8

Amiri, M., Bahrami, F., & Janahmadi, M. (2011). Functional modeling of astrocytes in epilepsy: a feedback system perspective. *Neural Computing and Applications*, 20(8), 1131–1139. https://doi.org/10.1007/s00521-010-0479-0

Amiri, M., Bahrami, F., & Janahmadi, M. (2012a). Functional contributions of astrocytes in synchronization of a neuronal network model. *Journal of Theoretical Biology*, 292, 60–70. https://doi.org/10.1016/j.jtbi.2011.09.013

Amiri, M., Bahrami, F., & Janahmadi, M. (2012b). Modified thalamocortical model: a step towards more understanding of the functional contribution of astrocytes to epilepsy. *Journal of Computational Neuroscience*, 33(2), 285–299. https://doi.org/10.1007/s10827-012-0386-8

Amiri, M., Bahrami, F., & Janahmadi, M. (2012c). On the role of astrocytes in epilepsy: a functional modeling approach. *Neuroscience Research*, 72(2), 172–180. https://doi.org/10.1016/j.neures.2011.11.006

Amiri, M., Hosseinmardi, N., Bahrami, F., & Janahmadi, M. (2013a). Astrocyte-neuron interaction as a mechanism responsible for generation of neural synchrony: a study based on modeling and experiments. *Journal of Computational Neuroscience*, 34(3), 489–504. https://doi.org/10.1007/s10827-012-0432-6

Amiri, M., Montaseri, G., & Bahrami, F. (2013b). A phase plane analysis of neuron-astrocyte interactions. *Neural Networks*, 44, 157–165. https://doi.org/10.1016/j.neunet.2013.03.018

Atri, A., Amundson, J., Clapham, D., & Sneyd, J. (1993). A single-pool model for intracellular calcium oscillations and waves in the Xenopus laevis oocyte. *Biophysical Journal*, 65(4), 1727–1739. https://doi.org/10.1016/S0006-3495(93)81191-3

Bennett, M. R., Farnell, L., & Gibson, W. G. (2005). A quantitative model of purinergic junctional transmission of calcium waves in astrocyte networks. *Biophysical Journal*, 89(4), 2235–2250. https://doi.org/10.1529/biophysj.105.062968

Bennett, M. R., Farnell, L., & Gibson, W. G. (2008). A quantitative model of cortical spreading depression due to purinergic and gap-junction transmission in astrocyte networks. *Biophysical Journal*, 95(12), 5648–5660. https://doi.org/10.1529/biophysj.108.137190

Chan, S.-C., Mok, S.-Y., Ng, D. W.-K., & Goh, S.-Y. (2017). The role of neuron–glia interactions in the emergence of ultra-slow oscillations. *Biological Cybernetics*, 111, 459–472. https://doi.org/10.1007/s00422-017-0740-z

Dayan, P. & Abbott, L. F. (2001). *Theoretical Neuroscience*. MIT Press.

De Pittà, M. & Brunel, N. (2016). Modulation of synaptic plasticity by glutamatergic gliotransmission: a modeling study. *Neural Plasticity*, 2016, 7607924. https://doi.org/10.1155/2016/7607924

De Pittà, M., Goldberg, M., Volman, V., Berry, H., & Ben-Jacob, E. (2009). Glutamate regulation of calcium and IP_3 oscillating and pulsating dynamics in astrocytes. *Journal of Biological Physics*, 35(4), 383–411. https://doi.org/10.1007/s10867-009-9155-y

De Pittà, M., Volman, V., Berry, H., & Ben-Jacob, E. (2011). A tale of two stories: astrocyte regulation of synaptic depression and facilitation. *PLoS Computational Biology*, 7(12), e1002293. https://doi.org/10.1371/journal.pcbi.1002293

De Pittà, M., Volman, V., Levine, H., Pioggia, G., De Rossi, D., & Ben-Jacob, E. (2008). Coexistence of amplitude and frequency modulations in intracellular calcium dynamics. *Physical Review E*, 77(3), 030903. https://doi.org/10.1103/PhysRevE.77.030903

De Young, G. W. & Keizer, J. (1992). A single-pool inositol 1,4,5-trisphosphate-receptor-based model for agonist-stimulated oscillations in Ca^{2+} concentration. *Proceedings of the National Academy of Sciences of the United States of America*, 89(20), 9895–9899. https://doi.org/10.1073/pnas.89.20.9895

Destexhe, A., Mainen, Z. F., & Sejnowski, T. J. (1994). Synthesis of models for excitable membranes, synaptic transmission and neuromodulation using a common kinetic formalism. *Journal of Computational Neuroscience*, 1(3), 195–230. https://doi.org/10.1007/BF00961734

Destexhe, A., Mainen, Z. F., & Sejnowski, T. J. (1998). Kinetic models of synaptic transmission. *Methods in Neuronal Modeling*, 1–25. MIT Press.

Dupont, G. & Goldbeter, A. (1993). One-pool model for Ca^{2+} oscillations involving Ca^{2+} and inositol 1,4,5-trisphosphate as co-agonists for Ca^{2+} release. *Cell Calcium*, 14(4), 311–322. https://doi.org/10.1016/0143-4160(93)90052-8

Esir, P. M., Gordleeva, S. Y., Simonov, A. Y., Pisarchik, A. N., & Kazantsev, V. B. (2018). Conduction delays can enhance formation of up and down states in spiking neuronal networks. *Physical Review E*, 98(5), 052401. https://doi.org/10.1103/PhysRevE.98.052401

Fink, C. C., Slepchenko, B., & Loew, L. M. (1999). Determination of time-dependent inositol-1, 4, 5-trisphosphate concentrations during calcium release in a smooth muscle cell. *Biophysical Journal*, 77(1), 617–628. https://doi.org/10.1016/S0006-3495(99)76918-3

FitzHugh, R. (1961). Impulses and physiological states in theoretical models of nerve membrane. *Biophysical Journal*, 1(6), 445–466. https://doi.org/10.1016/S0006-3495(61)86902-6

Gerstner, W. & Kistler, W. M. (2002). *Spiking Neuron Models: Single Neurons, Populations, Plasticity.* Cambridge University Press. https://doi.org/10.1017/CBO9780511815706

Goldberg, M., De Pittà, M., Volman, V., Berry, H., & Ben-Jacob, E. (2010). Nonlinear gap junctions enable long-distance propagation of pulsating calcium waves in astrocyte networks. *PLoS Computational Biology*, 6(8), e1000909. https://doi.org/10.1371/journal.pcbi.1000909

Gordleeva, S. Y., Ermolaeva, A. V., Kastalskiy, I. A., & Kazantsev, V. B. (2019). Astrocyte as spatiotemporal integrating detector of neuronal activity. *Frontiers in Physiology*, 10, 294. https://doi.org/10.3389/fphys.2019.00294

Gordleeva, S. Y., Lebedev, S. A., Rumyantseva, M. A., & Kazantsev, V. B. (2018). Astrocyte as a detector of synchronous events of a neural network. *JETP Letters*, 107(7), 440–445. https://doi.org/10.1134/S0021364018070032

Gordleeva, S. Y., Stasenko, S. V., Semyanov, A. V., Dityatev, A. E., & Kazantsev, V. B. (2012). Bi-directional astrocytic regulation of neuronal activity within a network. *Frontiers in Computational Neuroscience*, 6, 92. https://doi.org/10.3389/fncom.2012.00092

Guo, D. & Li, C. (2011). Signal propagation in feedforward neuronal networks with unreliable synapses. *Journal of Computational Neuroscience*, 30(3), 567–587. https://doi.org/10.1007/s10827-010-0279-7

Haghiri, S. & Ahmadi, A. (2020). An investigation on neuron–astrocyte interaction system: network behavior and synchronization. *Analog Integrated Circuits and Signal Processing*, 108, 569–576. https://doi.org/10.1007/s10470-020-01735-5

Haghiri, S., Ahmadi, A., & Saif, M. (2016). VLSI implementable neuron-astrocyte control mechanism. *Neurocomputing*, 214, 280–296. https://doi.org/10.1016/j.neucom.2016.06.015

Haghiri, S., Ahmadi, A., & Saif, M. (2017). Complete neuron-astrocyte interaction model: digital multiplierless design and networking mechanism. *IEEE Transactions on Biomedical Circuits and Systems*, 11(1), 117–127. https://doi.org/10.1109/TBCAS.2016.2583920

Hayati, M., Nouri, M., Haghiri, S., & Abbott, D. (2016). A digital realization of astrocyte and neural glial interactions. *IEEE Transactions on Biomedical Circuits and Systems*, 10(2), 518–529. https://doi.org/10.1109/TBCAS.2015.2450837

Hodgkin, A. L. & Huxley, A. F. (1952). A quantitative description of membrane current and its application to conduction and excitation in nerve. *Journal of Physiology*, 117(4), 500–544. https://doi.org/10.1113/jphysiol.1952.sp004764

Höfer, T., Venance, L., & Giaume, C. (2002). Control and plasticity of intercellular calcium waves in astrocytes: a modeling approach. *Journal of Neuroscience*, 22(12), 4850–4859. https://doi.org/10.1523/JNEUROSCI.22-12-04850.2002

Izhikevich, E. M. (2003). Simple model of spiking neurons. *IEEE Transactions on Neural Networks*, 14(6), 1569–1572. https://doi.org/10.1109/TNN.2003.820440

Jahr, C. E. & Stevens, C. F. (1990). Voltage dependence of NMDA-activated macroscopic conductances predicted by single-channel kinetics. *Journal of Neuroscience*, 10(9), 3178–3182. https://doi.org/10.1523/JNEUROSCI.10-09-03178.1990

Kager, H., Wadman, W. J., & Somjen, G. G. (2000). Simulated seizures and spreading depression in a neuron model incorporating interstitial space and ion concentrations. *Journal of Neurophysiology*, 84(1), 495–512. https://doi.org/10.1152/jn.2000.84.1.495

Kanakov, O., Gordleeva, S., Ermolaeva, A., Jalan, S., & Zaikin, A. (2019). Astrocyte-induced positive integrated information in neuron-astrocyte ensembles. *Physical Review E*, 99(1), 012418. https://doi.org/10.1103/PhysRevE.99.012418

Kazantsev, V. B. (2009). Spontaneous calcium signals induced by gap junctions in a network model of astrocytes. *Physical Review E*, 79(1), 010901. https://doi.org/10.1103/PhysRevE.79.010901

Kopell, N., Börgers, C., Pervouchine, D., Malerba, P., & Tort, A. (2010). Gamma and theta rhythms in biophysical models of hippocampal circuits. *Hippocampal Microcircuits. Springer Series in Computational Neuroscience, vol 5.*, 423–457. Springer. https://doi.org/10.1007/978-1-4419-0996-1_15

Kopell, N., Ermentrout, G. B., Whittington, M. A., & Traub, R. D. (2000). Gamma rhythms and beta rhythms have different synchronization properties. *Proceedings of the National Academy of Sciences of the United States of America*, 97(4), 1867–1872. https://doi.org/10.1073/pnas.97.4.1867

Lallouette, J., De Pittà, M., Ben-Jacob, E., & Berry, H. (2014). Sparse short-distance connections enhance calcium wave propagation in a 3D model of astrocyte networks. *Frontiers in Computational Neuroscience*, 8, 45. https://doi.org/10.3389/fncom.2014.00045

Larter, R. & Craig, M. G. (2005). Glutamate-induced glutamate release: a proposed mechanism for calcium bursting in astrocytes. *Chaos*, 15(4), 047511. https://doi.org/10.1063/1.2102467

Latham, P. E., Richmond, B. J., Nelson, P. G., & Nirenberg, S. (2000). Intrinsic dynamics in neuronal networks. I. Theory. *Journal of Neurophysiology*, 83(2), 808–827. https://doi.org/10.1152/jn.2000.83.2.808

Lemon, G., Gibson, W. G., & Bennett, M. R. (2003). Metabotropic receptor activation, desensitization and sequestration-I: Modelling calcium and inositol 1,4,5-trisphosphate dynamics following receptor activation. *Journal of Theoretical Biology*, 223(1), 93–111. https://doi.org/10.1016/S0022-5193(03)00079-1

Lenk, K., Priwitzer, B., Ylä-Outinen, L., Tietz, L. H. B., Narkilahti, S., & Hyttinen, J. A. K. (2016). Simulation of developing human neuronal cell networks. *BioMedical Engineering OnLine*, 15, 105. https://doi.org/10.1186/s12938-016-0226-6

Lenk, K., Satuvuori, E., Lallouette, J., Ladrón-de Guevara, A., Berry, H., & Hyttinen, J. A. K. (2020). A computational model of interactions between neuronal and astrocytic networks: The role of astrocytes in the stability of the neuronal firing rate. *Frontiers in Computational Neuroscience*, 13, 92. https://doi.org/10.3389/fncom.2019.00092

Li, J.-J., Du, M.-M., Wang, R., Lei, J.-Z., & Wu, Y. (2016). Astrocytic gliotransmitter: diffusion dynamics and induction of information processing on tripartite synapses. *International Journal of Bifurcation and Chaos*, 26(8), 1650138. https://doi.org/10.1142/S0218127416501388

Li, J. J., Xie, Y., Yu, Y. G., Du, M. M., Wang, R., & Wu, Y. (2017). A neglected GABAergic astrocyte: calcium dynamics and involvement in seizure activity. *Science China Technological Sciences*, 60(7), 1003–1010. https://doi.org/10.1007/s11431-016-9056-2

Li, L., Zhou, J., Sun, H., Liu, J., Wang, H., Liu, X., & Wang, C. (2020). A computational model to investigate GABA-activated astrocyte modulation of neuronal excitation. *Computational and Mathematical Methods in Medicine*, 2020, 8750167. https://doi.org/10.1155/2020/8750167

Li, Y.-X. & Rinzel, J. (1994). Equations for $InsP_3$ receptor-mediated $[Ca^{2+}]_i$ oscillations derived from a detailed kinetic model: a Hodgkin-Huxley like formalism. *Journal of Theoretical Biology*, 166(4), 461–473. https://doi.org/10.1006/jtbi.1994.1041

Liu, J., Harkin, J., Maguire, L. P., McDaid, L. J., Wade, J. J., & Martin, G. (2016). Scalable networks-on-chip interconnected architecture for astrocyte-neuron networks. *IEEE Transactions on Circuits and Systems I: Regular Papers*, 63(12), 2290–2303. https://doi.org/10.1109/TCSI.2016.2615051

Liu, Y. & Li, C. (2013a). Firing rate propagation through neuronal–astrocytic network. *IEEE Transactions on Neural Networks and Learning Systems*, 24(5), 789–799. https://doi.org/10.1109/TNNLS.2013.2245678

Liu, Y. & Li, C. (2013b). Stochastic resonance in feedforward-loop neuronal network motifs in astrocyte field. *Journal of Theoretical Biology*, 335, 265–275. https://doi.org/10.1016/j.jtbi.2013.07.007

Ma, J., Huang, L., Tang, J., Ying, H.-P., & Jin, W.-Y. (2012). Spiral wave death, breakup induced by ion channel poisoning on regular Hodgkin–Huxley neuronal networks. *Communications in Nonlinear Science and Numerical Simulation*, 17(11), 4281–4293. https://doi.org/10.1016/j.cnsns.2012.03.009

Mainen, Z. F., Joerges, J., Huguenard, J. R., & Sejnowski, T. J. (1995). A model of spike initiation in neocortical pyramidal neurons. *Neuron*, 15(6), 1427–1439. https://doi.org/10.1016/0896-6273(95)90020-9

Mainen, Z. F. & Sejnowski, T. J. (1996). Influence of dendritic structure on firing pattern in model neocortical neurons. *Nature*, 382(6589), 363–366. https://doi.org/10.1038/382363a0

Makovkin, S. Y., Shkerin, I. V., Gordleeva, S. Y., & Ivanchenko, M. V. (2020). Astrocyte-induced intermittent synchronization of neurons in a minimal network. *Chaos, Solitons & Fractals*, 138, 109951. https://doi.org/10.1016/j.chaos.2020.109951

Mazzoni, A., Panzeri, S., Logothetis, N. K., & Brunel, N. (2008). Encoding of naturalistic stimuli by local field potential spectra in networks of excitatory and inhibitory neurons. *PLoS Computational Biology*, 4(12), e1000239. https://doi.org/10.1371/journal.pcbi.1000239

Mesiti, F., Floor, P. A., & Balasingham, I. (2015). Astrocyte to neuron communication channels with applications. *IEEE Transactions on Molecular, Biological and Multi-Scale Communications*, 1(2), 164–175. https://doi.org/10.1109/TMBMC.2015.2501743

Morris, C. & Lecar, H. (1981). Voltage oscillations in the barnacle giant muscle fiber. *Biophysical Journal*, 35(1), 193–213. https://doi.org/10.1016/S0006-3495(81)84782-0

Nadkarni, S. & Jung, P. (2003). Spontaneous oscillations of dressed neurons: a new mechanism for epilepsy? *Physical Review Letters*, 91(26), 268101. https://doi.org/10.1103/PhysRevLett.91.268101

Nadkarni, S. & Jung, P. (2005). Synaptic inhibition and pathologic hyperexcitability through enhanced neuron-astrocyte interaction: a modeling study. *Journal of Integrative Neuroscience*, 4(2), 207–226. https://doi.org/10.1142/S0219635205000811

Nadkarni, S. & Jung, P. (2007). Modeling synaptic transmission of the tripartite synapse. *Physical Biology*, 4(1), 1–9. https://doi.org/10.1088/1478-3975/4/1/001

Naeem, M., McDaid, L. J., Harkin, J., Wade, J. J., & Marsland, J. (2015). On the role of astroglial syncytia in self-repairing spiking neural networks. *IEEE Transactions on Neural Networks and Learning Systems*, 26(10), 2370–2380. https://doi.org/10.1109/TNNLS.2014.2382334

Nazari, S., Amiri, M., Faez, K., & Van Hulle, M. M. (2020). Information transmitted from bioinspired neuron–astrocyte network improves cortical spiking network's pattern recognition performance. *IEEE Transactions on Neural Networks and Learning Systems*, 31(2), 464–474. https://doi.org/10.1109/TNNLS.2019.2905003

Nazari, S. & Faez, K. (2019). Empowering the impaired astrocytes in the tripartite synapses to improve accuracy of pattern recognition. *Soft Computing*, 23(17), 8307–8319. https://doi.org/10.1007/s00500-018-03671-z

Olufsen, M. S., Whittington, M. A., Camperi, M., & Kopell, N. (2003). New roles for the gamma rhythm: population tuning and preprocessing for the beta rhythm. *Journal of Computational Neuroscience*, 14(1), 33–54. https://doi.org/10.1023/A:1021124317706

Pinsky, P. F. & Rinzel, J. (1994). Intrinsic and network rhythmogenesis in a reduced Traub model for CA3 neurons. *Journal of Computational Neuroscience*, 1(1), 39–60. https://doi.org/10.1007/BF00962717

Postnov, D. E., Koreshkov, R. N., Brazhe, N. A., Brazhe, A. R., & Sosnovtseva, O. V. (2009). Dynamical patterns of calcium signaling in a functional model of neuron-astrocyte networks. *Journal of Biological Physics*, 35(4), 425–445. https://doi.org/10.1007/s10867-009-9156-x

Postnov, D. E., Ryazanova, L. S., & Sosnovtseva, O. V. (2007). Functional modeling of neural-glial interaction. *Biosystems*, 89(1), 84–91. https://doi.org/10.1016/j.biosystems.2006.04.012

Prescott, S. A., Ratté, S., De Koninck, Y., & Sejnowski, T. J. (2008). Pyramidal neurons switch from integrators in vitro to resonators under in vivo-like conditions. *Journal of Neurophysiology*, 100(6), 3030–3042. https://doi.org/10.1152/jn.90634.2008

Reddy, D. V. R., Sen, A., & Johnston, G. L. (2000). Dynamics of a limit cycle oscillator under time delayed linear and nonlinear feedbacks. *Physica D*, 144(3-4), 335–357. https://doi.org/10.1016/S0167-2789(00)00086-5

Sahlender, D. A., Savtchouk, I., & Volterra, A. (2014). What do we know about gliotransmitter release from astrocytes? *Philosophical Transactions of the Royal Society B*, 369(1654), 20130592. https://doi.org/10.1098/rstb.2013.0592

Shuai, J.-W. & Jung, P. (2002). Stochastic properties of Ca^{2+} release of inositol 1, 4, 5-trisphosphate receptor clusters. *Biophysical Journal*, 83(1), 87–97. https://doi.org/10.1016/S0006-3495(02)75151-5

Silchenko, A. N. & Tass, P. A. (2008). Computational modeling of paroxysmal depolarization shifts in neurons induced by the glutamate release from astrocytes. *Biological Cybernetics*, 98(1), 61–74. https://doi.org/10.1007/s00422-007-0196-7

Sneyd, J., Charles, A. C., & Sanderson, M. J. (1994). A model for the propagation of intercellular calcium waves. *American Journal of Physiology-Cell Physiology*, 266(1), C293–C302. https://doi.org/10.1152/ajpcell.1994.266.1.C293

Sneyd, J., Wetton, B. T. R., Charles, A. C., & Sanderson, M. J. (1995). Intercellular calcium waves mediated by diffusion of inositol trisphosphate: a two-dimensional model. *American Journal of Physiology-Cell Physiology*, 268(6), C1537–C1545. https://doi.org/10.1152/ajpcell.1995.268.6.C1537

Soleimani, H., Bavandpour, M., Ahmadi, A., & Abbott, D. (2015). Digital implementation of a biological astrocyte model and its application. *IEEE Transactions on Neural Networks and Learning Systems*, 26(1), 127–139. https://doi.org/10.1109/TNNLS.2014.2311839

Somjen, G. G., Kager, H., & Wadman, W. J. (2008). Computer simulations of neuron-glia interactions mediated by ion flux. *Journal of Computational Neuroscience*, 25(2), 349–365. https://doi.org/10.1007/s10827-008-0083-9

Stimberg, M., Goodman, D. F. M., Brette, R., & De Pittà, M. (2019). Modeling neuron–glia interactions with the Brian 2 simulator. *Computational Glioscience*, 471–505. Springer. https://doi.org/10.1007/978-3-030-00817-8_18

Suffczynski, P., Kalitzin, S., & Lopes Da Silva, F. H. (2004). Dynamics of non-convulsive epileptic phenomena modeled by a bistable neuronal network. *Neuroscience*, 126(2), 467–484. https://doi.org/10.1016/j.neuroscience.2004.03.014

Tang, J., Zhang, J., Ma, J., Zhang, G. Y., & Yang, X. Q. (2017). Astrocyte calcium wave induces seizure-like behavior in neuron network. *Science China Technological Sciences*, 60(7), 1011–1018. https://doi.org/10.1007/s11431-016-0293-9

Terman, D., Rubin, J. E., Yew, A. C., & Wilson, C. J. (2002). Activity patterns in a model for the subthalamopallidal network of the basal ganglia. *Journal of Neuroscience*, 22(7), 2963–2976. https://doi.org/10.1523/JNEUROSCI.22-07-02963.2002

Tsodyks, M., Pawelzik, K., & Markram, H. (1998). Neural networks with dynamic synapses. *Neural Computation*, 10(4), 821–835. https://doi.org/10.1162/089976698300017502

Ullah, G., Jung, P., & Cornell-Bell, A. H. (2006). Anti-phase calcium oscillations in astrocytes via inositol (1,4,5)-trisphosphate regeneration. *Cell Calcium*, 39(3), 197–208. https://doi.org/10.1016/j.ceca.2005.10.009

Volman, V., Ben-Jacob, E., & Levine, H. (2007). The astrocyte as a gatekeeper of synaptic information transfer. *Neural Computation*, 19(2), 303–326. https://doi.org/10.1162/neco.2007.19.2.303

Wade, J., McDaid, L., Harkin, J., Crunelli, V., & Kelso, S. (2012). Self-repair in a bidirectionally coupled astrocyte-neuron (AN) system based on retrograde signaling. *Frontiers in Computational Neuroscience*, 6, 76. https://doi.org/10.3389/fncom.2012.00076

Wallach, G., Lallouette, J., Herzog, N., De Pittà, M., Jacob, E. B., Berry, H., & Hanein, Y. (2014). Glutamate mediated astrocytic filtering of neuronal activity. *PLoS Computational Biology*, 10(12), e1003964. https://doi.org/10.1371/journal.pcbi.1003964

Yang, Y. & Yeo, C. K. (2015). Conceptual network model from sensory neurons to astrocytes of the human nervous system. *IEEE Transactions on Biomedical Engineering*, 62(7), 1843–1852. https://doi.org/10.1109/TBME.2015.2405549

Yao, W., Huang, H., & Miura, R. M. (2011). A continuum neuronal model for the instigation and propagation of cortical spreading depression. *Bulletin of Mathematical Biology*, 73(11), 2773–2790. https://doi.org/10.1007/s11538-011-9647-3

Yao, W., Huang, H., & Miura, R. M. (2018). Role of astrocyte in cortical spreading depression: a quantitative model of neuron-astrocyte network. *Communications in Computational Physics*, 23(2), 440–458. https://doi.org/10.4208/cicp.OA-2016-0262

Yu, Y., Yuan, Z., Fan, Y., Li, J., & Wu, Y. (2020). Dynamic transitions in neuronal network firing sustained by abnormal astrocyte feedback. *Neural Plasticity*, 2020, 8864246. https://doi.org/10.1155/2020/8864246
